# Proteome Remodeling of the Eye Lens at 50 Years Identified With Data-Independent Acquisition

**DOI:** 10.1016/j.mcpro.2022.100453

**Published:** 2022-12-05

**Authors:** Lee S. Cantrell, Romell B. Gletten, Kevin L. Schey

**Affiliations:** 1 Vanderbilt University Mass Spectrometry Research Center, Nashville, Tennessee, USA; 2 Vanderbilt University Chemical and Physical Biology Program, Nashville, Tennessee, USA; 3 Vanderbilt University Department of Biochemistry, Nashville, Tennessee, USA

**Keywords:** lens, data-independent acquisition, long-lived proteins, aging, deamidation, AQP0/1/5, aquaporin 0, 1, and 5, ARNC, age-related nuclear cataract, DDA, data-dependent acquisition, DIA, data-independent acquisition, DIA-NN, DIA-neural network, EAAT, excitatory amino acid transporter, 4F2, cell-surface antigen heavy chain, FDR, false discovery rate, GO, Gene Ontology, MCS, microcirculatory system, MRP, multidrug resistance protein, MS, mass spectrometry, NaDC3, sodium-dependent dicarboxylate transporter 3, Na/K, sodium/potassium, OAT3, organic anion transporter 3, PC1, principal component axis 1, PCA, principal component analysis, PSEA, Protein Set Enrichment Analysis, PTM, post-translational modification, SLC, solute carrier protein, SVCT2, sodium-coupled vitamin C transporter 2, TEAB, triethylamine bicarbonate, TMM, trimmed mean of M-values, UPS, ubiquitin proteasome system

## Abstract

The eye lens is responsible for focusing and transmitting light to the retina. The lens does this in the absence of organelles, yet maintains transparency for at least 5 decades before onset of age-related nuclear cataract (ARNC). It is hypothesized that oxidative stress contributes significantly to ARNC formation. It is in addition hypothesized that transparency is maintained by a microcirculation system that delivers antioxidants to the lens nucleus and exports small molecule waste. Common data-dependent acquisition methods are hindered by dynamic range of lens protein expression and provide limited context to age-related changes in the lens. In this study, we utilized data-independent acquisition mass spectrometry to analyze the urea-insoluble membrane protein fractions of 16 human lenses subdivided into three spatially distinct lens regions to characterize age-related changes, particularly concerning the lens microcirculation system and oxidative stress response. In this pilot cohort, we measured 4788 distinct protein groups, 46,681 peptides, and 7592 deamidated sequences, more than in any previous human lens data-dependent acquisition approach. Principally, we demonstrate that a significant proteome remodeling event occurs at approximately 50 years of age, resulting in metabolic preference for anaerobic glycolysis established with organelle degradation, decreased abundance of protein networks involved in calcium-dependent cell–cell contacts while retaining networks related to oxidative stress response. Furthermore, we identified multiple antioxidant transporter proteins not previously detected in the human lens and describe their spatiotemporal and age-related abundance changes. Finally, we demonstrate that aquaporin-5, among other proteins, is modified with age by post-translational modifications including deamidation and truncation. We suggest that the continued accumulation of each of these age-related outcomes in proteome remodeling contribute to decreased fiber cell permeability and result in ARNC formation.

## Highlights

• The human lens proteome undergoes remodeling with age, appearing at 50 years.
• Gap junctions and aquaporins are truncated or otherwise modified with age.
• SLC24A2 was identified as a calcium transporter significantly modified with age.
• Measurement offers insights toward lens microcirculation and oxidative homeostasis.

## Graphical Abstract

**Figure:**
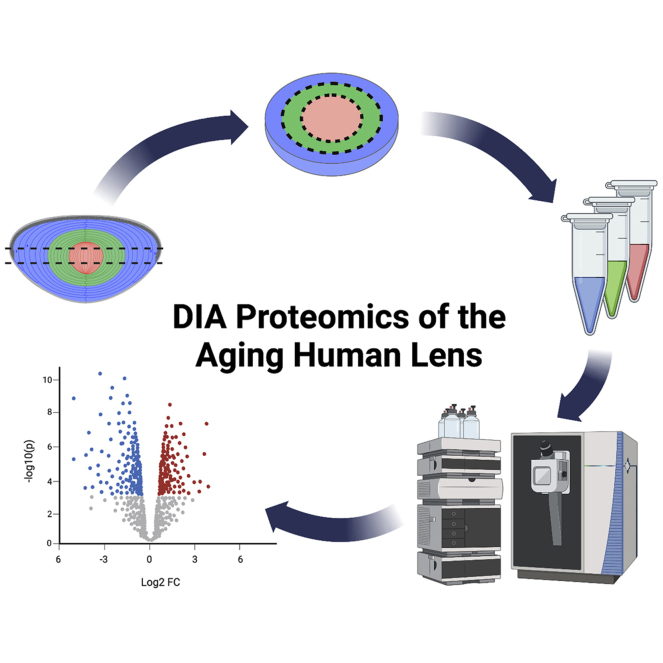

## In Brief

The lens microcirculation system is essential for maintenance of lens transparency. To evaluate maintenance of the microcirculatory system, we measured three developmentally distinct fiber cell populations within 16 human lenses of increasing age. Using data-independent acquisition, we identified a proteome remodeling event that occurs at approximately 50 years of age. Age-modified proteins included gap junctions, a calcium transporter, aquaporin-5, and oxidative stress response–related proteins. Remodeling may result in calcium-activated protease activity and disruption of the microcirculation system.

**Fig. 1:**
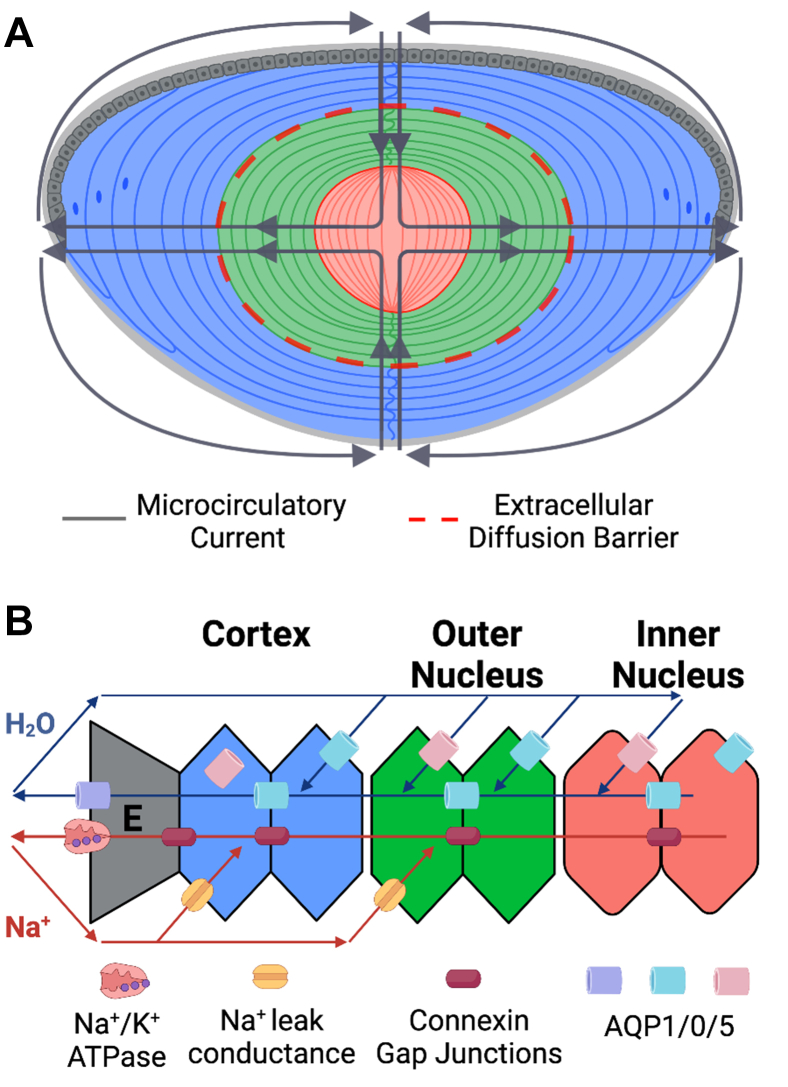
***Cartoons* of the lens and microcirculatory system (MCS).***A*, *cartoon* of the lens with fiber cells divided into cortex (*blue*), outer nucleus (*green*), and inner nucleus (*red*) with the net convection of the MCS displayed. The approximate positioning of the extracellular diffusion barrier is noted within the inner nucleus. *B*, *cartoon* of the electromotive potential establishment and net current in the cross section of cells in the MCS. Sodium/potassium ATPases at the epithelium (E) transport sodium from the lens, with reuptake enabled by sodium leak conductance channels in fiber cells. Sodium transport is enabled by aquaporin-0 and aquaporin-5 in fiber cells and aquaporin-1 in epithelial cells. The current established by microcirculation allows small-molecule metabolites to transport intercellularly through connexin gap junctions at the cross section of fiber cells. Figure adapted from Schey *et al*. (12).

The ocular lens is a transparent tissue lacking vasculature and is responsible for light transmission to the retina for visual perception (1). The lens originates from primary fiber cells that differentiate from epithelial cells in utero. Throughout life, concentric growth rings of secondary fiber cells are added to the lens, differentiating at the lens equator from an anterior monolayer of epithelial cells to elongated fiber cells that extend toward the anterior and posterior poles of the lens (Fig. 1*A*). Unlike most cell types, lens fiber cells are not degraded throughout life but experience organelle degradation and enter a senescent-like state shortly after elongation as part of cellular maturation (2). Thus, proteins in the center of the lens are effectively as old as the subject, and proteins are spatially organized, as are fiber cells, in concentric growth rings.

Lens protein oxidation, particularly in the lens inner nucleus, is hypothesized to contribute to formation of age-related nuclear cataract (ARNC) (3, 4). ARNC is the most common form of blindness, and pharmacological options for delaying onset, prevention, or reversal are lacking. In a young lens, oxidative stress response is primarily mediated by GSH-requisite proteins (5, 6, 7, 8, 9, 10). In the absence of vasculature, small molecules including water and GSH must be delivered to the inner nucleus of the lens through the extracellular space of the lens sutures, established at the anterior and posterior poles of the lens between the tips of fiber cells (8).

The delivery of antioxidants and efflux of waste products is hypothesized to be established by the lens microcirculatory system (MCS) (11). In the MCS, small molecules are delivered through the lens sutures to the inner nucleus, taken up by fiber cells, and are exported through the equator by a concerted network of intercellular junctions (connexins) and water channels (12). Exported molecules are then convected around the lens exterior toward the anterior and posterior poles before re-entering the lens. Molecular convection is established by an electromotive potential of sodium/potassium (Na/K) ATPases transporting sodium out of the lens at the equator, whereas concurrent export of potassium is mediated by potassium channels on the lens epithelium (13, 14). The resulting potential, coupled to aquaporin (AQP) water transport and gap junction intercellular contacts, leads to a net current of metabolite convection into the lens at the anterior and posterior poles (15, 16) (Fig. 1*B*).

In the young lens, and especially in young cortical fiber cells, MCS functionality is sufficient to transport water and metabolites through the lens (17, 18). However, convection of small molecules to the lens nucleus and through progressively aged lenses is inhibited by a barrier to diffusion (6, 17). This barrier is formed and becomes kinetically significant by 40 to 60 years of age (17). After barrier formation, small molecules may enter the lens through the sutures but do not proceed through the restricted extracellular space at the same rate as in the young lens. Thus, as the lens ages and GSH is not delivered to the lens nucleus, proteins incur oxidative damage at an accelerated rate relative to the young lens (3, 4, 6, 18). Protein byproducts of oxidative damage may ultimately result in protein misfolding, crosslinking, aggregation, and light scattering ARNC (4, 19).

Measurement of the lens proteome has previously been achieved by measurement of whole lens lysates, imaging mass spectrometry (MS), and spatially targeted approaches employing manual separation or laser capture microdissection prior to sample analysis (20, 21, 22, 23, 24, 25, 26). A limitation to all lens proteomics experiments is the high abundance of crystallin proteins, reported to constitute up to 90% of the lens proteome (27). Although age-related modifications to lens crystallins, such as abundance change, deamidation, and truncation, have been identified, a substantial gap in our knowledge exists in understanding how the MCS changes at the molecular level as a function of age. In this study, we use data-independent acquisition (DIA) MS coupled to a membrane protein enrichment strategy to measure the membrane proteome of human lenses at different ages. To quantitatively measure spatiotemporal changes, we have divided the lens into three regions with distinct biological functions: the inner nucleus, which contains primary fiber cells formed in utero alongside the oldest secondary fiber cells of the lens; the outer nucleus, where fiber cells are fully matured and reside on the interior side of the diffusion barrier (Fig. 1*A*); and the cortex, which is composed of the youngest mature secondary fiber cells, differentiating secondary fiber cells with organelles still intact and a monolayer of epithelial cells. Proteome measurement in each of these regions is critical for understanding how age and barrier establishment perturb protein machinery responsible for MCS establishment and delivery of antioxidants to the inner nucleus.

## Experimental Procedures

### Experimental Design and Statistical Rationale

Sixteen human lenses from age 15 to 74 years were analyzed with sample selection guided by known age-related lens physiology. Exclusion criteria included several cataract comorbidities, such as diabetes mellitus, nicotine use, and non–age-related cataract. In addition, no cataract lenses were used in this study. These studies were conducted in accordance with the ethical standards of the institutional research committee and with the 1964 Helsinki declaration and its later amendments. Each of the 16 lenses was divided into three regions (cortex, outer nucleus, and inner nucleus) to yield a total of 48 samples in the dataset. For sample quality control, 0.75 μg MassPREP protein standard was added to each approximately 75 μg lysate to monitor digest efficiency between replicates.

Samples were analyzed with DIA without prefractionation, with MS1 spectra interspersed every 30 to 31 scans. Each of the 48 samples analyzed in the study were used to generate a spectral library in DIA-neural network (DIA-NN) (version 1.8.0) (28) with an initial *in silico* library predicted by DIA-NN against a UniProt SwissProt canonical human fasta database with nine MassPREP spike in proteins added (UP000005640, downloaded October 12, 2021; 20,402 entries). From this initial library, a subset of experimentally measured spectra were used to create an experiment-specific library. After generation of the experimental spectral library, samples were reanalyzed, and proteins were quantified. No retention time standards were used; instead, endogenous peptide retention times were used for alignment in DIA-NN. Each sample was prepared and analyzed in an order determined by a random number generator, and a blank gradient was run between samples to minimize carry over and quantitative bias. Trimmed mean of M-values (TMM) normalization was employed for sample normalization. Sample groups were defined by exploratory data analysis that revealed clustering of lenses before and after 50 years of age. Within the young lens cohort, nine lenses were analyzed (aged 15, 18, 22, 34, 34, 41, 44, 46, and 49 years), and seven lenses were measured in the old lens cohort (aged 53, 57, 63, 64, 65, 68, and 74 years). Sample size of this pilot cohort was set at 16 to demonstrate changes that occur in human lenses on a protein network level and protein abundance changes that occur over a gradient of human age. Statistical significance of change was assessed with two-sample *t* tests and Protein Set Enrichment Analysis (PSEA)-Quant operated in “labeled” analysis mode (29).

### Urea-Insoluble Protein Isolation

The severity of cataract present in each eye was visually evaluated prior to tissue processing to confirm that advanced cataractous lenses were not analyzed. Mild yellowing of the lens was allowed, but each measured lens retained transparency. In addition to sample evaluation, deidentified ophthalmic medical history was reviewed to confirm that no cataract had been diagnosed. Samples were prepared as previously described (30). Briefly, lenses were mounted with the equatorial axis parallel to a cryostat chuck before removal of the anterior and posterior poles of the lens yielding a 1.0 mm thick equatorial lens section. Concentric biopsy centerpunches were taken at 4.5 and 7 mm diameter to yield inner nucleus (0–4.5 mm), outer nucleus (4.5–7.0 mm), and cortex (7.0–9.2 mm) samples. Samples were then enriched for membrane proteins by a previously optimized method (30). Briefly, tissue was hand homogenized in buffer containing 25 mM Tris (pH 8), 5 mM EDTA, 1 mM DTT, 150 mM NaCl, and 1 mM PMSF. After homogenization, samples were centrifuged at 100,000*g* for 30 min, and the supernatant was discarded. Pellets were washed twice with the aforementioned homogenization buffer followed by washes with 3.5 M and 7 M urea added to the homogenization buffer. Centrifugation at 100,000*g* for 30 min was performed to separate the supernatant and pellets for each urea wash. The remaining urea-insoluble pellet was taken up in 50 mM triethylamine bicarbonate (TEAB) with 5% SDS, and protein concentration was measured with a bicinchoninic assay.

Membrane pellets of urea-insoluble sample (75 μg total protein) were suspended in 50 mM TEAB with 5% SDS. Spike-in of 0.75 μg MassPREP protein standard was added before DTT was added to 10 mM before incubation at 56 °C for 1 h to reduce disulfide bonds. Reduced cysteines were alkylated by adding iodoacetamide to 20 mM and incubating in the dark at room temperature for 30 min. Phosphoric acid was added to 2.5% to acidify proteins. Acidified proteins were precipitated onto the S-Trap (ProtiFi) membrane bed with six working volume equivalents of cold 100 mM TEAB in 90% methanol according to ProtiFi protocol. Samples were washed on the S-Trap with 100 mM TEAB in 90% methanol four times with centrifugation between steps to remove salts and detergents. Samples were then digested in the S-Trap with a 1:15 trypsin:protein ratio in 20 μl 50 mM TEAB, pH 7.5 for 2 h at 46 °C. Digested peptides were eluted in four steps of 50 mM TEAB, 0.2% formic acid, 50 mM TEAB, and 50% acetonitrile. Eluted peptides were dried under vacuum centrifugation and taken up in 0.2% formic acid prior to data acquisition.

### Instrumentation and Data Analysis

Peptides were analyzed using a Dionex Ultimate 3000 UHPLC coupled to an Exploris 480 tandem mass spectrometer (Thermo Fisher Scientific). An in-house pulled capillary column was created from 75 μm inner diameter–fused silica capillary packed with 1.9 μm ReproSil-Pur C18 beads (Dr Maisch) to a length of 250 mm. Solvent A was 0.1% aqueous formic acid, and solvent B was 0.1% formic acid in acetonitrile. Approximately 200 ng peptide was loaded and separated at a flow rate of 200 nl/min on a 95 min gradient from 2 to 29% B, followed by a 14 min washing gradient and 35 min blank injection between runs. The exact gradient was determined by linearized separation of the top 50% most intense cortical peptide signals by the Gradient Optimization Analysis Tool (GOAT, version 1.0.1) (31).

For DIA, the Exploris 480 instrument was configured to acquire 61 × 20 *m/z* (390–1010 *m/z*) precursor isolation window DIA spectra (30,000 resolution, automatic gain control target 1e6, maximum ion injection time 55 ms, 27 normalized collision energy) using a staggered window pattern with window placements optimized by Thermo Fisher XCalibur instrument controls. Precursor spectra (385–1015 *m/z*, 60,000 resolution, automatic gain control target 3e6, maximum ion injection time 100 ms) were interspersed after each sequence of 30 to 31 MS/MS spectra of the mass range. Default charge state was set to +3, S-lens radiofrequency level set at 40%, normalized collision energy set at 27, and data were collected in profile mode. Each scan cycle of 63 spectra took approximately 4.4 s.

For analysis of DIA data, RAW files were converted to mzML files in ProteoWizard msConvert (32), with staggered window deconvolution performed to improve precursor specificity to a pseudo 10 *m/z* window width. Processed DIA files were searched in DIA-NN with an Intel Core i7-7700 CPU at 3.60 GHz utilizing eight threads. For all searches, up to one missed trypsin cleavage was allowed on peptides 7 to 30 residues in length with N-terminal M excision and cysteine carbamidomethylation enabled. All fragments between *m/z* 200 and 1800 and in charge states +1 to 4 were considered. An initial spectral library was prepared by DIA-NN with deep learning–based spectra and retention time prediction against a UniProt SwissProt canonical human fasta database (UP000005640, downloaded October 12, 2021; 20,402 entries) with a predicted trypsin/P protease used. In each search, the neural network classifier was run in double pass mode with likely interferences removed, quantitation was performed in robust LC (high accuracy) mode, and crossrun normalization was turned off. Two separate searches were performed, one without variable modification and one with up to one variable deamidation on asparagine or glutamine (+0.984016 Da). An initial search of all files produced a spectral library that was used to search the data a second time (termed match between runs). After a search of files against the experimental spectral library (7504 proteins and 55,875 peptides in the library built without modifications and 5659 proteins and 27,639 peptides in deamidation-enabled library), precursors and protein groups were filtered at 1% false discovery rate (FDR) within DIA-NN.

### Statistical Analysis

Statistical analysis was initiated through custom R scripts on peptides having <1% *q* value and <1% global protein *q* value. Prior to protein assembly, all peptides within the MaxQuant contaminant list were removed (33). Proteins were only assembled on peptides considered proteotypic, and abundances for peptides and proteins were calculated by the diann R package function diann_maxlfq (https://github.com/vdemichev/diann-rpackage). Because lens samples were not of high (>90%) quantitative similarity by Pearson correlation of DIA-NN normalized protein abundances, normalization performed in DIA-NN was rejected for quantitation of peptides and proteins. To minimize the assumption employed by most normalization algorithms that all compared samples are congruent, a subset list of peptides and proteins detected in all samples was selected and TMM normalization was applied (34). TMM applies a linear multiplier for normalization, which was extracted and uniformly applied to all rows of the peptide and protein matrices, including rows where missing values were present. Subsequent comparisons between lenses of different age are indicative of the representative contribution of a protein to the urea-insoluble lens proteome. The distribution of peptides and proteins was qualitatively assessed to ensure that TMM treatment produced similar abundance distributions between samples (supplemental Fig. S1). DIA analysis assumes that all measurable proteins are detected; thus, no missing value imputation was used.

Statistical significance between young and old lenses was calculated with a two-sample *t* test to minimize overfitting of data that had been log2 treated. Significance cutoff was set to a *p* value of 0.01 for hierarchical clustering and volcano plot visualizations. Volcano plot significance was further established at 1.5 log2 fold change. Statistical power of two-sample *t* tests was calculated with RnaSeqSampleSize package in R (35). PSEA-Quant was used to evaluate significance on an ontology level without arbitrary significance cutoffs (29). PSEA-Quant was implemented as recommended by the developers with enrichment between sample groups considered instead of enrichment within a single sample. Default parameters for PSEA-Quant were used, with iterations increased to 1,000,000 to enable empirical *p* value assignment as low as 1/1,000,000.

### Data Visualization and Presentation

All data visualizations were produced in R with either the ggplot2, heatmap.2, GGally, or EnhancedVolcano packages. Principal component analysis (PCA) from the prcomp package was used to separate proteins based on relative protein abundance for all proteins without missing values in the compared dataset (number of proteins used in caption of each PCA figure). PCA processing included scaling of non–log2 transformed data. For hierarchical clustering, a Ward-based approach was used to produce the dendrogram of proteins below the 0.01 *p* value threshold (number of proteins in caption). Subsequent visualization was done with the heatmap.2 package. Equivalent statistics were used to plot the volcano plots with the EnhancedVolcano package.

For PSEA-Quant visualizations, the list of significant protein network ontologies exceeds visually interpretable space, so terms returned from PSEA-Quant search were manually filtered based on FDR and *p* value to 0.1 and 0.01, respectively. The remaining terms were manually reduced to eliminate unspecific or redundant terms from the visualization. A full list of significant ontologies is available in supplemental Tables S2–S7. Finally, selected protein abundance was visualized respective to age and lens region with corresponding linear trend lines calculated. For comparison of deamidation accumulation, the results from the DIA search with one variable deamidation were prepared as described, and the ratio of deamidated peptide accumulation in each sample was calculated as deamidated peptide abundance/(deamidated + undeamidated abundances) to demonstrate the proportional accumulation of deamidation with age. Biological cartoons were drawn at BioRender.com. Protein networks were visualized with STRING (36) and GOView (http://www.webgestalt.org/2017/GOView/).

### Quality Control

Samples were prepared and were ran in random order. A bovine serum albumin standard was run every eight samples to verify integrity of retention time, instrument calibration, and chromatographic similarity. Several samples were reinjected to match total ion current intensity and subsequently control for variability in quantitation of peaks with different peak shapes. The initial injections of these samples were not used in spectral library development or statistical analysis. The MassPREP protein standard was spiked into protein lysates prior to digestion to monitor digestion efficiency by sequence coverage and Pearson correlation profile of peptide abundances.

## Results

### DIA of the Lens Proteome

To measure proteins associated with the membrane and intercellular interactions as key components of the lens MCS, we employed DIA on membrane and insoluble protein fractionated lens lysates from human lenses of different age. Fiber cells of increasing age were measured by separating the lens into three age-indexed regions: the cortex (youngest cells), outer nucleus, and inner nucleus (oldest cells). In total, 16 human lenses were measured (15–74 years old) resulting in 48 total samples. Each region described here approximately corresponds to growth regions previously defined by electron microscopy and MRI analysis of lens fiber cell morphology and physiology (11, 37). To evaluate the prevalence of deamidation, an age-related modification, we performed two distinct DIA data searches. The first was based on a spectral library with no variable modifications, and the second was based on a spectral library with up to one variable deamidation. We used deamidation as a proxy for age-related accumulation of post-translational modifications (PTMs) that are less measurable with unenriched DIA approaches utilizing library-free search. In total, 4788 distinct protein groups and 46,681 distinct peptides, not including modified sequences, were identified at 1% FDR. In addition, 7592 deamidated sequences were identified. In this study, DIA facilitated measurement of 2.5-fold more lens proteins than any previous human lens data-dependent acquisition (DDA) method (30). The resulting data are evaluated as the representative contribution of each protein within the lens membrane or insoluble proteome. Although lens proteins are not synthesized after fiber cell maturation, the accumulation or positive differential representation of proteins suggests a reduced rate of degradation of a protein relative to its constituent proteome or the accumulation of aggregated or otherwise insolubilized cytosolic proteins.

### Determination of Sample Grouping by Age

**Fig. 2:**
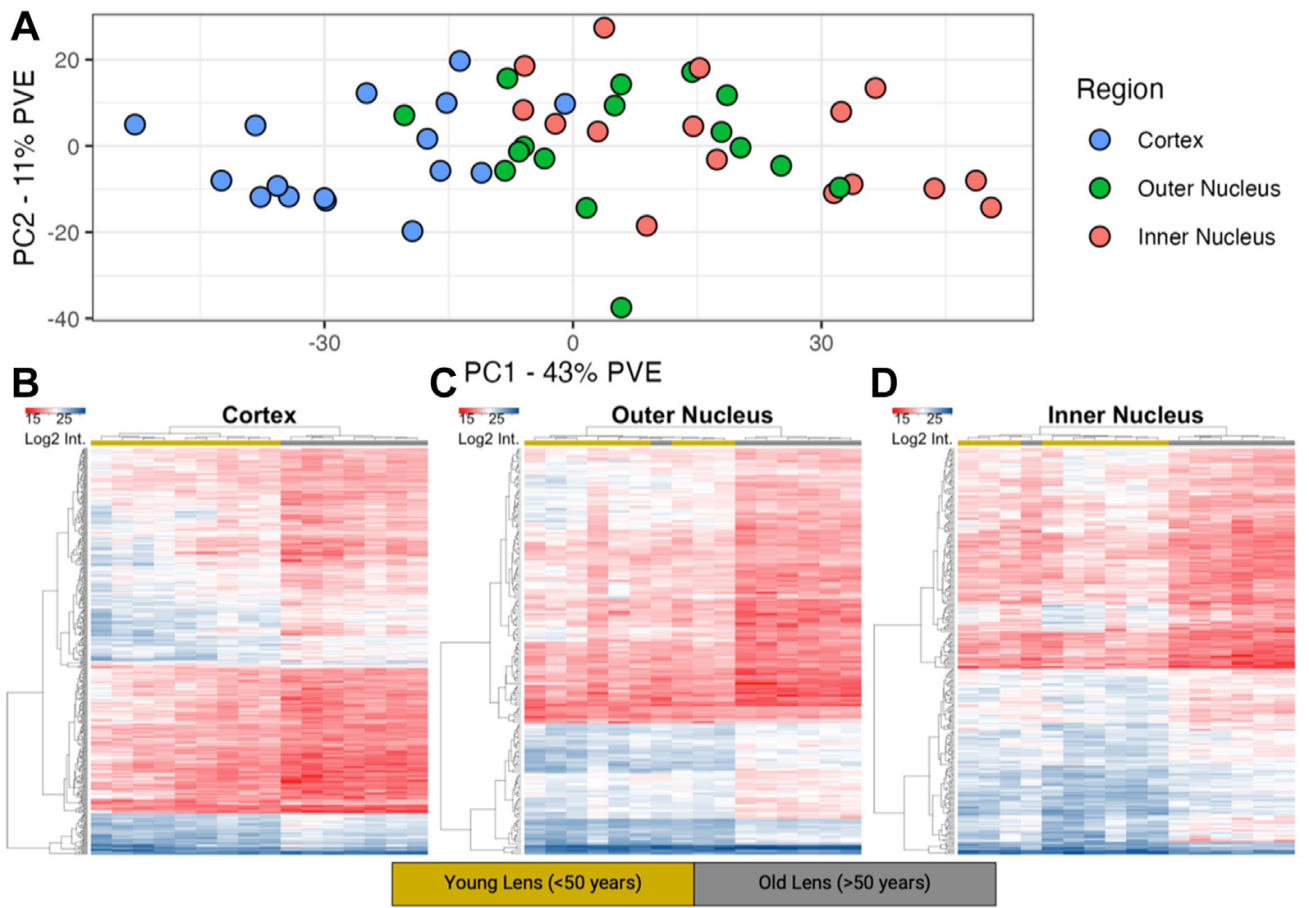
**Determination of age groups in the human lens based on fiber cell positioning and age.***A,* principal component analysis (PCA) on all 48 samples based on only protein groups measured in all samples (n = 884). Each point was colored according to lens region as in Figure 1. *B,* cortex hierarchical clustering demonstrates clustering of proteins differentially present in samples above and below 50 years. Significance was measured by a *t* test with a 0.01 significance cutoff (n = 432). Columns colored by *t*-test sample grouping. Protein group names presented in Supplemental Table S1 if protein log2 fold change between groups exceeds 1.5. *C,* outer nucleus hierarchical clustering presented as in (B) (n = 170). *D,* inner nucleus hierarchical clustering presented as in (B) (n = 278). Full size graphics with subject-age annotated columns are reproduced in Supplemental Figure S3–S5.

A primary goal of this study was to identify key subsets of protein networks that are preserved in the aging lens since they may relate to long-term maintenance of transparency. First, we performed PCA on proteins identified in all samples without imputation (n = 884) (Fig. 2*A*). Relative to conventional cell line model systems (38), few proteins were identified in all samples, which is reflective of long-lived protein degradation, modification, and of human biological variability. Separation along principal component axis 1 (PC1) showed a progressive transition of fiber cells from young cortical fiber cells to old inner nucleus fiber cells with some overlap of middle-aged fiber cell populations (*e.g.*, old cortical, young inner nucleus, and all outer nucleus fiber cells). Cortical lens region separation on PC1 was explained by cytoskeletal elements periaxin, vimentin, and neurofilament medium polypeptide; cadherin junction proteins cadherin-2 and catenin alpha-2; and brain acid–soluble protein 1 (BASP1). Consistent with lens proteome investigations, older nuclear fiber cells were most associated with crystallins γ-A/B/C/D and β-B1; crystallin species that are known to associate with the membrane with age (20, 26, 30, 39). Further supporting the biological integrity of normalization approach employed, intermediate filament proteins were enriched in the young still maturing cortical fiber cells as previously reported (23). Spearman correlation between samples was also performed, demonstrating similarity between samples of the same region and progressive decrease in correlation between samples of increasing age difference (supplemental Fig. S2).

From PCA of all samples, no clear sample separation of subject age emerges. Age grouping was instead defined using hierarchical clustering of proteins with *t* test significance at a 0.01 *p* value between lenses below 50 years (young) and above 50 years (old). This preliminary cutoff was established congruent with the hypothesis of barrier formation at 45 to 50 years old (40). To evaluate consistency of biological changes directly associated with human age, each region of the lens was separately evaluated by hierarchical clustering (Figs. 2, *B*–*D* and S3–S5). As demonstrated in the dendrograms of each lens region, there is an appreciable change in the clustering of lenses before and after 50 years of age with single sample deviations for the 64-year-old inner nucleus and 53-year-old outer nucleus. To our knowledge, no prior dataset of the lens has demonstrated the clear separation of lenses observed at 50 years as shown here. These results suggest that a biological event occurs after approximately 50 years of aging that triggers a proteome remodeling event throughout the lens. As a result of these findings, we considered sample groups in the lens as young or old based on a 50-year cutoff.

### Age-Related Changes in the Lens Cortex

To demonstrate changes at the single-protein and protein-network level in the aging lens cortex, a three-step approach was taken. First, we performed PCA on all protein groups identified in each cortex sample. While the cortical data approximately clustered in Figure 2*A*, young and old lens fibers are separated on PC1 of the subset samples (supplemental Fig. S6). A volcano plot analysis was done to evaluate all proteins that change between young and older lenses (supplemental Fig. S7 and supplemental Table S1), with few proteins measured as increased in the old cortex relative to the young cortex.

**Fig. 3:**
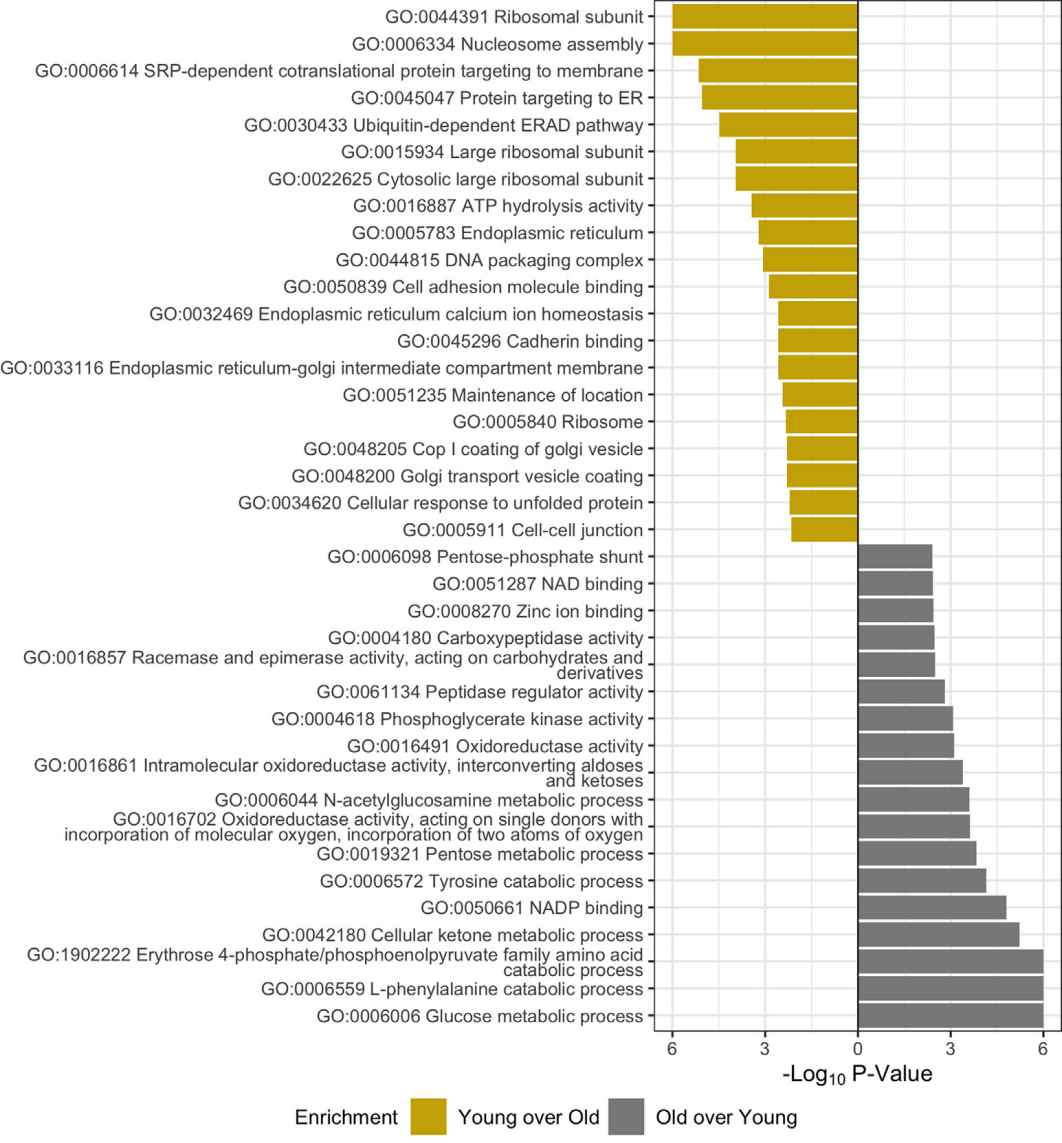
**PSEA-Quant pairwise enrichment calculated between young and old cortex fiber cell regions.** Separate calculations were performed to determine each enrichment. Significant Gene Ontology terms were filtered at 0.01 *p* value and 0.1 false discovery rate. Ontologies in graphics are a minimal subset of all measured, demonstrating nonredundant daughter terms indicative of the complete enrichment set. Full list of enriched terms are included in Supplemental Tables S2 and S3.

PSEA-Quant was employed to identify protein networks that are most changed with age. Unlike *t* tests, PSEA-Quant eliminates arbitrary *p* value cutoffs by establishing enrichment scores as done in the Gene Set Enrichment Algorithm (41). The results file of significant Gene Ontology (GO) terms was filtered consistent with PSEA-Quant developer suggestions (Fig. 3*C*, supplemental Tables S1 and S3) (29). GO network relationship graphs and predicted protein–protein interaction analysis demonstrates relationships of ontology terms and associated proteins (supplemental Figs. S8 and S9). As expected, young lenses with intact organelle machinery still maintain ribosome, endoplasmic reticulum, Golgi, and other classical organelle structures (GO:0044391, GO:0048200, and GO:0005783). While these organelle structures are present in both young and old lenses, the proportion of young and still maturing fiber cells is greater in the young lens than in the old lens. These young fiber cells also demonstrate canonical proteostasis enrichment relative to old fiber cells (GO:0030433 and GO:0032469). Proteins involved in proteostasis include hsp70 BIP, hsp90 endoplasmin, wolframin, and DNAJB2. In the older cortical fiber cells, the results suggest that metabolic remodeling occurs as mitochondria are degraded, transitioning from oxidative phosphorylation to anaerobic glycolysis *via* the hexose monophosphate shunt pathway (GO:0051287 and GO:0005911). It was also shown that cell–cell junctions are enriched in the young fiber cells relative to old and that oxidative stress response machinery is enriched in old fiber cells relative to young. Based on this result, we hypothesize that NADH production machinery is established in cortical fiber cells to metabolically support enzymes responsible for GSH-mediated oxidative stress response in aging fiber cells. Note that in-text graphics of PSEA-Quant results are a subset of all significant GO enrichment terms and were manually filtered by eliminating redundant or unspecific terms.

### Age-Related Changes in the Lens Outer Nucleus

**Fig. 4:**
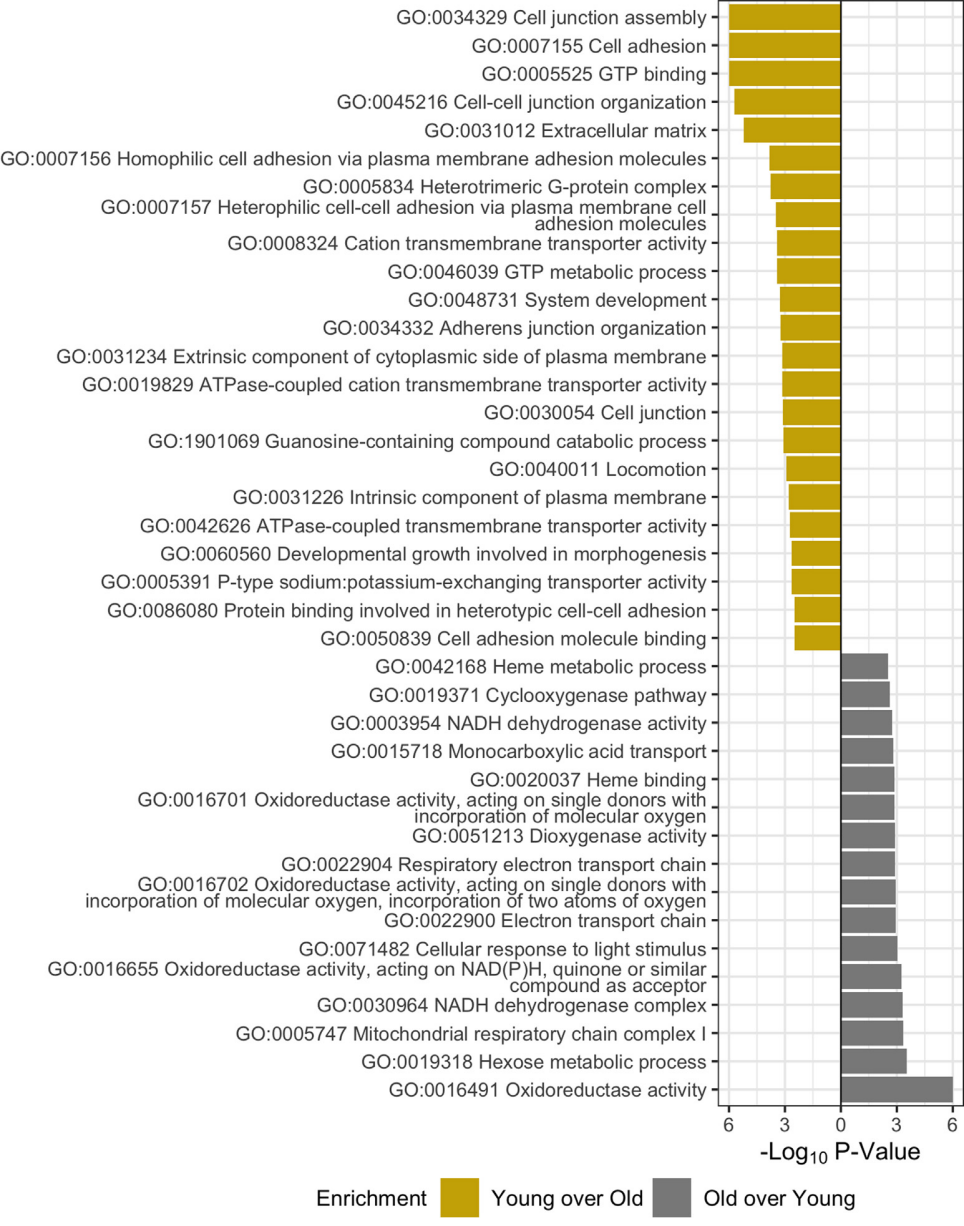
**PSEA-Quant pairwise enrichment calculated between young and old outer nucleus fiber cell regions.** Separate calculations were performed to determine each enrichment. Significant Gene Ontology terms were filtered at 0.01 *p* value and 0.1 false discovery rate. Ontologies in graphics are a minimal subset of all measured, demonstrating nonredundant daughter terms indicative of the complete enrichment set. Full list of enriched terms are included in Supplemental Tables S4 and S5.

In the outer nucleus, PCA (supplemental Fig. S10) demonstrates separation of young lenses from old lenses congruent to Figure 2*C*. However, a single sample from the old cohort (53 years old) did not separate according to the prescribed trend, as also demonstrated in hierarchical clustering (Fig. 2*C*). Two-sample *t* testing of young and old lens outer nuclei did not yield as many significantly changed proteins as in the cortex (supplemental Fig. S11 and supplemental Table S1). However, PSEA-Quant was successfully used to enrich for ontologies differentiated with age, followed by enriched ontology network analyses (Figs. 4, S12 and S13, and supplemental Tables S4 and S5). In the outer nucleus, the prevailing theory is that anaerobic metabolism established in the cortex is less active and that remodeling of the proteome is exclusively attributed to age-related changes caused by intercellular signaling and oxidative homeostasis not to transcriptional control. This is demonstrated by the enrichment of GO terms related to gap junction (GO:0034329) and cell-to-cell contacts (GO:0007156) in young lenses and enriched representation of oxidoreductase (GO:0016491, GO:0016655, and GO:0016701) protein networks in old lenses relative to young lenses. Each of these trends are expected to be a continuation of the aging process observed in the cortex where transcription is eliminated. Specific analysis of proteins related to respiratory electron transport reveals most of these proteins (UniProt identifiers: O96168, O43574, Q86Y39, O95298, Q9P0J0, and O43181) are NADH dehydrogenases. It is expected that if these dehydrogenases are functionally active, they are not contributing to mitochondrial respiratory electron chain function because organelles are degraded in the outer nucleus.

### Age-Related Changes in the Lens Inner Nucleus

The inner nucleus was evaluated as done for the cortex and outer nucleus regions. In PCA (supplemental Fig. S14), young and old lenses separated on PC1 precisely at the 50-year cutoff. In volcano plot analysis (supplemental Fig. S15 and supplemental Table S1), no proteins were significantly represented in the old lenses relative to young lenses. The low number of statistically enriched old inner nucleus proteins relative to young inner nucleus abundances is likely because of accumulation of age-related modifications including deamidation (21, 42, 43, 44). Deamidation is the most abundant nonisobaric irreversible PTM in the lens and randomly occurs on disordered region of client proteins, which is the region most likely to be measured in a membrane proteome measurement (22). Therefore, we examined the extent of deamidation as an explanation for decreased protein group abundance with age. We suggest that if the abundance of the unmodified peptide decreases and the deamidated peptide does not increase to a similar extent, that an alternative PTM is occurring, for example, truncation.

**Fig. 5:**
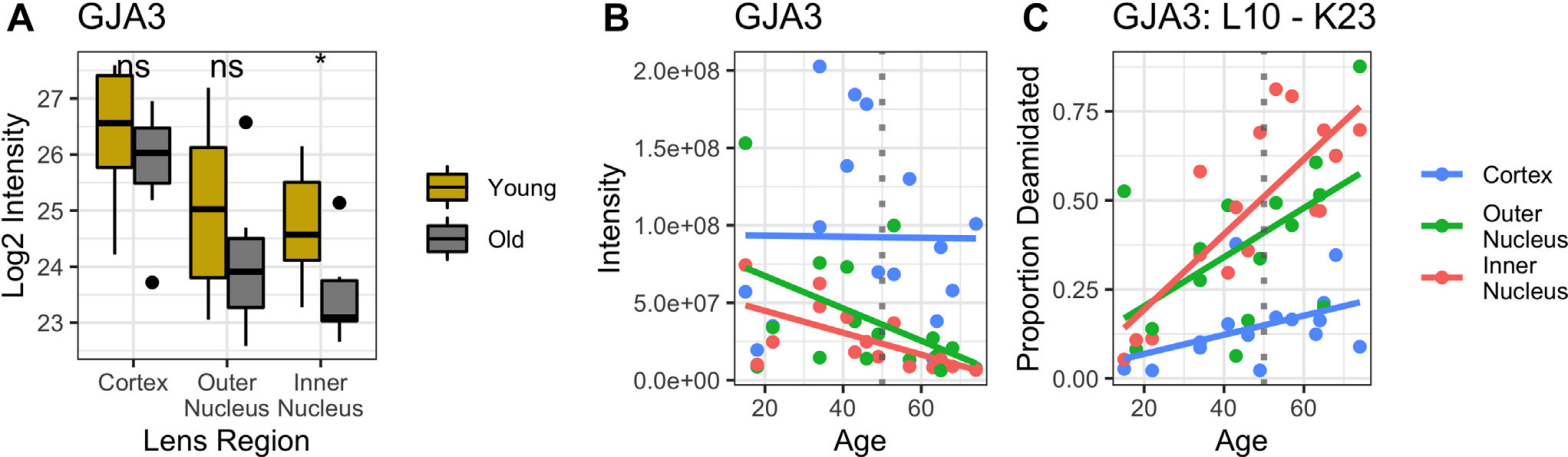
**Representative abundance of connexin 46 (GJA3) in each sample.***A*, protein abundance of GJA3 in a search with no variable modifications and *t* test significance of distributions (∗<0.05, ∗∗<0.01, and ∗∗∗<0.001) between young and old lenses. *B*, individual representative intensity of GJA3 signal in unmodified search, linear trend line plotted to demonstrate trend. *C*, representative deamidation accumulation of GJA3 L10-K23 calculated on single variable deamidation dataset with linear trend line to show accumulation of modification.

To demonstrate the impact of deamidation with age, we plotted the age-related abundance of connexin 46 (GJA3) when no variable modifications were included in the spectral library alongside the abundance of deamidated peptide L10-K23 measured in a separate search where a single deamidation modification was enabled (Fig. 5). Figure 5*A* demonstrates that there is a modest decrease in the abundance of GJA3 in the inner nucleus with age (*p* < 0.05). This may be partially explained by the accumulation of deamidation on the L10-K23 peptide (Fig. 5*C*, proportional deamidation being the measured intensity of deamidated peptide divided by the sum of deamidated and unmodified peptides). When protein abundance change is calculated with deamidated and unmodified peptides combined, the statistical significance of abundance change is not observed (supplemental Fig. S16).

**Fig. 6:**
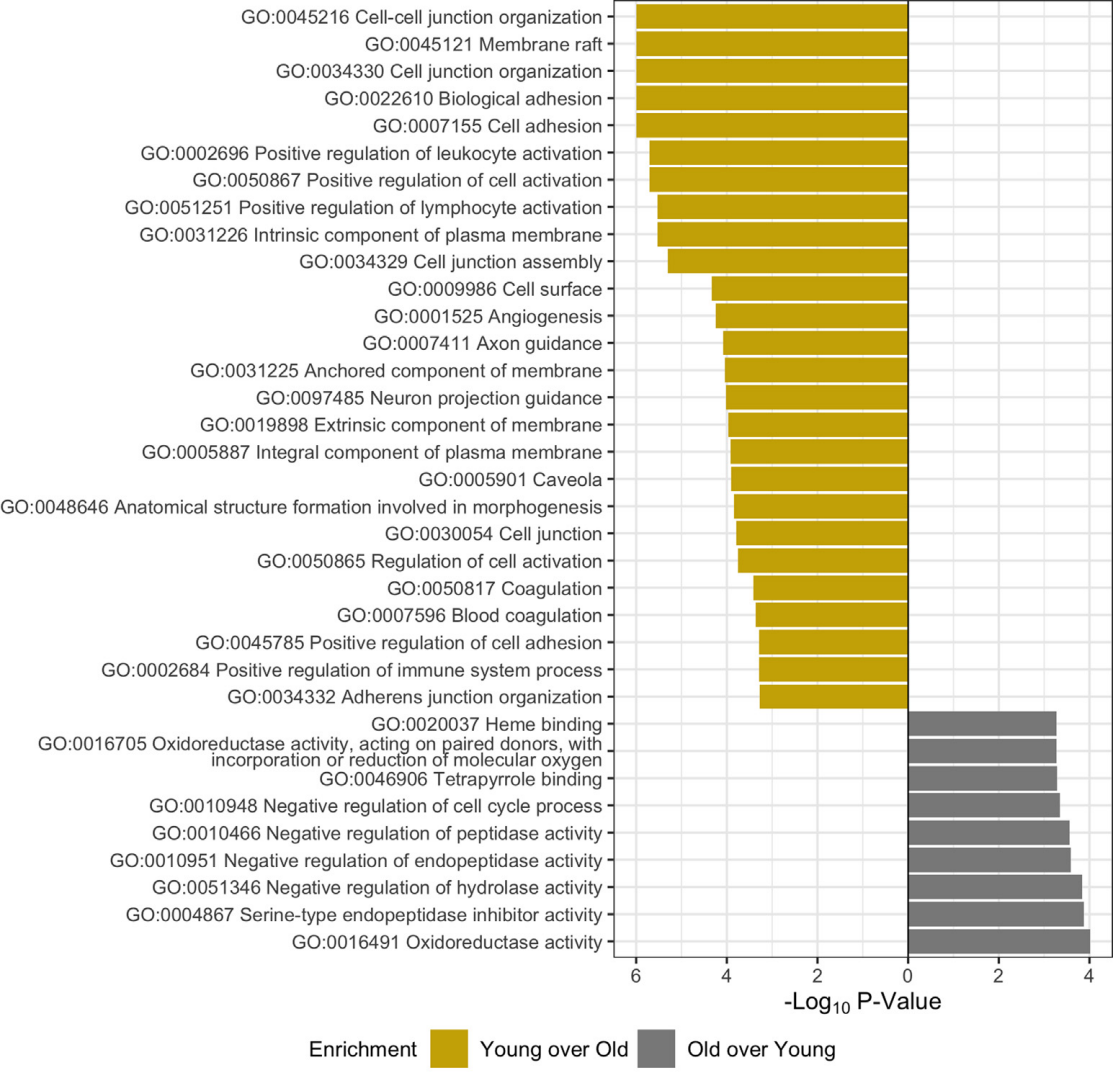
**PSEA-Quant pairwise enrichment calculated between young and old inner nucleus fiber cell regions.** Separate calculations were performed to determine each enrichment. Significant Gene Ontology terms were filtered at 0.01 *p* value and 0.1 false discovery rate. Ontologies in graphics are a minimal subset of all measured, demonstrating nonredundant daughter terms indicative of the complete enrichment set. Full list of enriched terms are included in Supplemental Tables S6 and S7.

Within inner nucleus PSEA-Quant results and network analysis (Fig. 6, supplemental Figs. S17, S18, supplemental Tables S6 and S7), oxidoreductase-related terms (GO:0016491 and GO:0016705) and negative regulation of several protein or peptide truncation ontologies (GO:0004867 and GO:0010948) were among the few ontologies enriched in the old inner nucleus relative to the young inner nucleus. Peptide truncation ontologies were represented by protease inhibitors (UniProt identifiers: P30740, Q92530, P04080, P35237, and P50453). The consistent identification of enriched oxidoreductase activity in each old lens region relative to the young lens supports the hypothesis that a primary function in the aging lens is reduction of oxidative stress, and that this functionality is preserved by sustained protein abundances. Likewise, protein truncation ontology enrichment suggests that the lens proteome attempts to preserve intact protein structure and function. A final observation from the PSEA-Quant results is the continued enrichment of cellular adhesion–related terms in young lenses relative to their old counterparts (GO:0045216, GO:0034330, GO:0007155, and GO:0034329). The inferred depletion of these terms in old lenses demonstrates that age may be responsible for depletion of cellular adhesion and intercellular metabolite transport. Taken together, we propose that while putatively active oxidative stress protein networks are inhibited by the decrease of cellular contacts and metabolite transport in the lens that accumulate with age.

## Discussion

In this study, we have measured more proteins than previously detected in the human lens with DDA, exceeding all prior approaches in total protein groups identified by 2.5-fold (30). As a label-free analysis in samples with putatively high biological variability, it was important to confirm previously described lens biology in the present analysis. Previous studies comparing progressively aged fiber cells have been performed and are consistent with the results presented here and with the MCS hypothesis (20, 23). In addition to considering aging fiber cells, Truscott *et al.* (26) previously evaluated human lens aging with isobaric tags. While those results are less rich than those afforded by DIA, several similarities can readily be made. In the study by Truscott and the presented work, the measured abundance of β-crystallin and γ-crystallin variants was increased in progressively aged lenses (see supplemental data for protein group intensities). Each study showed no significant age-related change in the abundances of α-crystallin A and B subunits, AQP0. Similar distributions of connexin proteins were also observed in each study. Finally, each study demonstrates age-related decline in the abundance of BASP1, paralemmin 1, and vimentin. The single disagreement between these studies is the inner nucleus measurement of GJA3: both studies suggest that the abundance is decreased significantly in older lenses, but we find that this change is insignificant when considering deamidation as a variable modification (supplemental Fig. S16). Deamidation was not considered in the prior study. Taken together, these results suggest that the label-free DIA analysis employed here is sensitive to measurement of known changes in the lens proteome with age.

As presented in the introduction section, oxidative stress response must occur in the inner nucleus, where fiber cells are metabolically less active and are not connected to a vascular transport system. Stress response is then thought to be mediated by the lens MCS delivery of reduced GSH and other metabolites to the inner nucleus through extracellular space along fiber cell sutures and the export of metabolites through intercellular contacts (gap junctions) toward the equator of the lens. As such, this discussion of proteome measurements is primarily focused on the oxidative stress response and the physiology of the MCS before and after 50 years of age.

### Antioxidant Protein Networks

Previous lens proteome studies have identified putative transporters of GSH and its precursors but not in the human lens (8, 10, 45). GSH uptake from extracellular suture space is putatively mediated by organic anion transporter 3 (OAT3) and sodium-dependent dicarboxylate transporter 3 (NaDC3), and efflux is mediated by multidrug resistance proteins 4 and 5 (MRP4/5) and connexins 46 and 50 (GJA3/8). The transport of GSH-precursor amino acids (glutamate, cysteine, and glycine) is also important for *in vivo* synthesis of GSH. These transport proteins include glycine transporters sodium- and chloride-dependent glycine transporters 1 and 2 (GLYT1/2), glutamine/glutamate transporter neutral amino acid transporter B(0) (ASCT2), glutamate and sodium cotransporters excitatory amino acid transporters 1 and 2 (EAAT1/2), and cysteine/glutamate antiporter complex (System X_c_^−^) composed of 4F2 cell-surface antigen heavy chain (4F2 or SLC3A2) and light-chain cysteine/glutamate transporter (XCT or SLC7A11). The functionality of GSH synthesis in the lens is beyond the scope of this study, but we note that the rate-limiting enzyme of GSH synthesis—glutamate cysteine ligase catalytic subunit is measured in all 48 samples, but its abundance is not changed with age. Finally, while GSH is believed to be the antioxidant most responsible for lens homeostasis, ascorbic acid (vitamin C) is also proposed to be transported through the lens as an antioxidant in its oxidized form *via* glucose transporter 1 and its reduced form by sodium-coupled vitamin C transporter 2 (SVCT2) as an alternative route for oxidative stress response.

For each of the proteins described previously, the frequency of identification and description of sample distribution is given in Table 1 (graphical abundances in supplemental Fig. S19). From these data, several key observations were made. First, the most ubiquitous antioxidant transporter measured in the membrane is OAT3, facilitating uptake of intact GSH. Age does not appear to have an effect on the abundance of OAT3 in the membrane. Next, that NaDC3, MRP4, EAAT1, and SVCT2 are only measured in the youngest cortical samples indicates little importance in long-lived proteome homeostasis. System X_c_^−^, a two-protein complex thought to contribute to cysteine accumulation in the lens nucleus for thiol protection, is not measured as intact in these samples as XCT is not measured in any sample. Concurrently, 4F2 is less abundant in the membrane with increasing fiber cell age and subject age. Finally, glucose transporter 1 is measured in all samples, and its abundance does not change with age. The loss of NaDC3, MRB4, EAAT1, and SVCT2 measurement in older fiber cells and the age-related reduction in 4F2 measurement suggest that GSH and vitamin C transport occurs in the lens, but that affinity for transport is reduced with aging. Future experiments should be directed toward specific measurement of missing or otherwise low-abundance antioxidant transporters to further support the spatial regulation and the absence of these transporter proteins.

**Table 1:** Proteins involved in antioxidant transport in the lens, their putative function, detection in the lens cohort, and approximate age-related distributions

Protein	Function	Detection	Comments
OAT3	GSH influx	40/48 samples	Approximate uniform distribution
NaDC3	GSH influx	3/48 samples	Only in young cortex
MRP4	GSH efflux	3/48 samples	Only in young cortex
MRP5	GSH efflux	6/48 samples	Decreasing expression with age; only in middle-aged lenses (34–63 years old)
GLYT1	Glycine transport	3/48 samples	Only in young cortex
GLYT2	Glycine transport	0/48 samples	Not measured
ASCT2	Glutamine/glutamate transport	0/48 samples	Not measured
EAAT1	Glutamate transport	3/48 samples	Only in young cortex
EAAT2	Glutamate transport	10/48 samples	Only in cortex, age-related decrease; not in oldest cortex
4F2/SLC3A2	Cysteine glutamate antiporter	34/48 samples	Most abundant in cortex, age-related decrease; not in old outer/inner nucleus
XCT/SLC7A11	Cysteine glutamate antiporter	0/48 samples	Not measured
GCLC	Rate-limiting enzyme in GSH synthesis	48/48 samples	Indistinct trend, approximately uniform
GLUT1	Dehydroascorbic acid transporter	48/48	Age-related decrease; high abundance in inner nucleus
SVCT2	Vitamin C transport	3/48	Only in young cortex

Abbreviations: GCLC, glutamate cysteine ligase catalytic subunit; GLUT1, glucose transporter 1.

Visualized abundances are represented in supplemental Fig. S6.

Our DIA analysis allowed additional novel observations to be made regarding oxidative stress response in the lens. First, PCA separation of cortical fiber cells showed that kynurenine oxoglutarate transaminase, responsible for formation of kynurenic acid from kynurenine and oxoglutarate, is enriched in older subject donors. Interestingly, kynurenic acid is shown to upregulate the activity of the glutamate cysteine ligase catalytic transcription factor Nrf2, suggesting a role for the transaminase in oxidative stress response in transcriptionally regulated cortical fiber cells (46). We also identified several key cytosolic proteins involved in GSH-mediated oxidative stress response. These include glutathione-*S*-transferase and GSH reductase, which are ubiquitously measured in each region of the lens (supplemental Fig. S20). Each of these proteins show a slight increase in measured intensity at approximately 50 years, but no age-dependent statistical significance is detected. The consistent measure of glutathione-*S*-transferase and GSH reductase in all lenses studied suggests that oxidoreductase pathways may be at least partially functional in older lenses.

### Physiology of the Lens MCS

#### Ion Gradient Establishment

Na/K ATPases along the epithelium are responsible for the establishment of a sodium cation gradient in the lens. It is not immediately apparent that these ATPases are modified in abundance in young or old lenses, suggesting that there is little change in expression with respect to age. This measurement may be artifactual as fiber cells were not separated from the epithelium, where these ATPases are functionally assigned in the MCS. Coupled to Na/K ATPases, a nonselective sodium leak channel is hypothesized to be responsible for the continuous influx of sodium to mature fiber cells. A suggested protein responsible for this nonselective sodium leak channel, NALCN, was not measured in any sample. The functional alternative to NALCN as a leak channel is GJA3, which does not decrease in abundance as a result of the proteome remodeling event. Previous biophysical studies have identified GJA3 as critical in the maintenance of sodium conductivity in the lens (47) but not as a leak channel explicitly.

#### Cell–Cell Junctions

A consistently measured change in PSEA-Quant analysis for each region of the lens was age-related depletion of cell–cell adhesion and junctions (*e.g.*, GO:0005911, GO:0034329, and GO:0045216). Cell–cell junctions of the lens are largely established by connexin gap junctions, actin-network tight junctions, and AQP0 (16, 23, 48). Connexin proteins play a key part in the transport of metabolites in the lens *via* formation of GJA3 and GJA8 gap junctions. For GJA3, abundance change is not observed (Fig. 6), but there are decreases in GJA8 abundance with fiber cell age and proteome remodeling (supplemental Fig. S21). These decreases cannot be explained solely by deamidation, as represented by age-related accumulation of GJA8 G265–K273 proportional deamidation (supplemental Fig. S21), and it instead suggested that a real decrease in protein abundance by truncation occurs. From prior DDA analyses, it is known that connexins are truncated at the N terminus, C terminus, or undergo cleavage in a central cytoplasmic loop (49). Neither gap junction is functionally restricted by truncation of the N or C terminus; however, truncation by cleavage at a midsequence cytoplasmic loop restricts hemichannel formation (49). Evaluating the cytoplasmic loop peptide E110–K139, we show that this peptide decreases similarly to the whole GJA8 protein group in the cortex but is not detected in inner nucleus samples older than 43 years (supplemental Fig. S21*D*). In the outer nucleus, we measured this peptide in lenses up to 53 years old. This demonstrates that GJA8 undergoes progressive decreases in abundance, but in mature fiber cells of older lenses, is likely nonfunctional, decreasing net connexin gap junction permeability.

Nonconnexin junctional proteins decreased with the age-related remodeling in the cell junction, or cytoskeleton-associated structural protein family of terms include vinculin, cell adhesion molecule 1/2/3, lens fiber membrane intrinsic protein 2, and cadherin-2. Many of the proteins associated with cell–cell and cell–junction ontologies are calcium dependent for the maintenance of adhesion. The functional relevance of the decreased abundance of these proteins in older lenses is unclear, but similar cell junction ontology terms to those measured here have been detected in previous analysis of progressively older fiber cell populations (20, 30). Calcium acts as a second messenger, and in addition to calmodulin-dependent inhibition of water permeability through AQP-0, regulates actin cytoskeleton integrity and the activity of the ubiquitin proteasome system (UPS), and calpain proteases (50, 51, 52). Prior measurements of the lens demonstrate that there is a parabolic decrease of calcium concentration in the lens with age, reaching its lowest point around 50 years, and increasing in lenses thereafter, with significantly elevated levels of calcium in cataract lenses (53). The accumulation of GO terms presented here led us to evaluate the effect of aging on selective calcium transporters.

#### Calcium Transport

To evaluate age-related changes in calcium transporters, we first selected the subset of broadly defined ATP-independent solute carrier (SLC) membrane proteins. Of 121 SLC protein groups, only 21 candidates were measured in at least 36 of 48 samples. This allowed filtering for SLC proteins that may be completely absent in old inner nucleus but required that they be measured at young age, when calcium export is putatively functional. From this subset list of 21 SLC proteins, we evaluated if age-related changes in abundance, consistent with the proteome remodeling event at 50 years, occurred. Many SLC proteins undergo statistically significant reduction in representation with age in the cortex, but this is anticipated to be a result of decreased organelle concentration and loss of protein synthesis in old fiber cells relative to young fiber cells. Thus, we elected to only identify candidate SLC proteins where statistical reduction occurred in at least two lens regions, reducing the 21 SLC candidate proteins to two proteins of interest. The first is SLC30A1, a relatively low-abundance zinc transporter not measured in the old inner nucleus and significantly decreased in the outer nucleus. Interestingly, the only other protein that showed consistent age-related decrease in abundance was SLC24A2, a light-activated sodium, potassium, and calcium exchange protein. The abundance profile of SLC24A2 with age (supplemental Fig. S22) demonstrates little change in the cortex, but especially interior to the established diffusion barrier, a remodeling-dependent decrease in abundance occurs. No other members of the sodium, potassium, and calcium exchange protein family were detected.

**Fig. 7:**
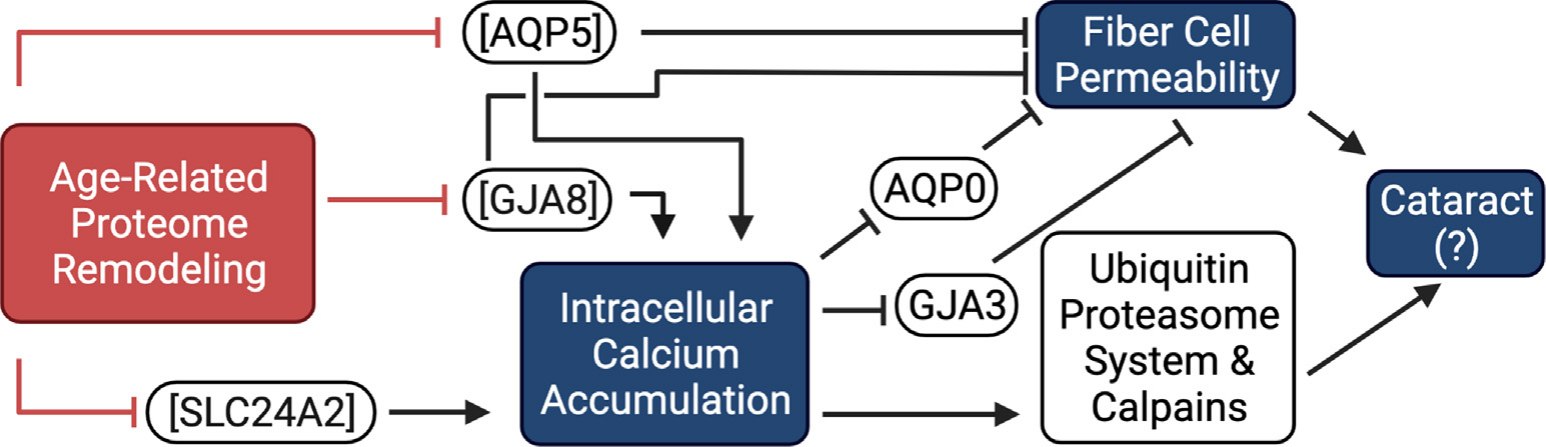
– **Schematic of suggested effect of age-related proteome remodeling.** Remodeling results in decreased abundance of AQP5, decreasing fiber cell permeability and decreases GJA3 and SLC24A2 abundances, resulting in calcium accumulation. Calcium accumulation inhibits GJA8 and AQP0 functionality, further inhibiting fiber cell permeability. Fiber cell permeability inhibition may then result in cataract. Calcium accumulation also results in ubiquitin proteasome and calpain protease activation, which may lead to cataract. Activation, →, and inhibition, ⊥, is indicated in reference to proteome remodeling treatment and not necessarily young lens function. AQP, aquaporin.

It is unclear whether SLC proteins contribute to intracellular transport after the extracellular space between fiber cells is decreased with fiber cell maturation. However, considering biophysical evidence, we suggest that the age-related proteome remodeling event at 50 years of age is responsible for functional restriction of calcium export from lens fiber cells. Thus, if SLC24A2 contributes to calcium export, it is expected that its degradation is a precursor to calcium accumulation and subsequent signaling events. The functional alternative to SLC24A2 for calcium transport is through nonselective connexin gap junction channels. Recent studies show that GJA8 is permeable to calcium (54). In contrast, calcium binding to GJA3 decreases channel permeability, and age-related accumulation of calcium may lead to inhibition of GJA3 function. In the context of GJA3 and GJA8 age-related abundances (Figs. 6 and S21), we suggest that the age-related decrease in GJA8 abundance and function may inhibit net calcium export from the lens resulting in calcium accumulation, which in turn inhibits the permeability of GJA3, for which abundance is not significantly decreased with age. Taken together, it is anticipated that proteome remodeling at 50 years directly contributes to calcium accumulation in the lens and that accumulation may lead to inhibition to MCS as a whole. The physiological significance of this being that a current of metabolic waste in old lenses is less efficient than in young lenses, identifying a potential role of calcium in cataract formation (Fig. 7).

Aside from MCS functionality, prior studies establish the UPS as functional in mature fiber cells, which lack significant metabolic activity (55). Indeed, components of the proteasome are not age degraded (supplemental Fig. S23). Our data suggest that calcium accumulation then results in UPS activation as in other cell types, initiating elevated rates of protein degradation and E3 ligase–conferred substrate specificity (50). As a result, UPS activity may contribute to degradation of oxidative stress response proteins leading to further accumulation of reactive oxygen species and oxidized substrate proteins responsible for ARNC. Finally, it is anticipated that calcium accumulation results in activation of calpain proteases, including calpain-2 catalytic subunit (P17655), which is measured in all 48 samples with no age-related change in abundance (52). In K6W ubiquitin model systems, UPS is inactive, and in the presence of accumulated calcium, calpain-associated fragmentation occurs on critical proteins, including filensin, vimentin, and β-crystallin. These lenses also formed cataract, further supporting a role of calcium accumulation in ARNC formation.

#### AQPs

Previous publications have thoroughly reviewed the importance of AQPs in the lens MCS (12, 21, 56, 57, 58, 59). Briefly, AQPs serve as water channels that facilitate water permeability in fiber cells (48). AQP0 is the most abundant membrane protein in lens fiber cells, and it undergoes truncation of the C terminus as an age-related modification throughout the lens (58). Truncation of AQP0 has been visually mapped with imaging MS (21). AQP-5 is a second less abundant AQP family member measured in lens fiber cells. Though structurally similar to AQP0, AQP5 has approximately 20-fold higher water permeability than AQP0 (60). Unlike AQP0, which does not undergo any significant change in abundance with the proteome remodeling event, AQP5 undergoes a significant decrease in abundance with age in the inner nucleus (supplemental Fig. S24). To evaluate if a significant change to AQP5 abundance is derived from deamidation, we evaluated the accumulation of deamidation on AQP5 S189–R198. The change in proportional deamidation on this peptide is incongruent with the magnitude of protein abundance decline, which suggests that deamidation does not account for the decrease in measured AQP5 abundance. Thus, we speculate that AQP5 is further modified in an age-dependent manner, which may include truncation. The functional implication of AQP5 modification may be reduced export of water from the inner nucleus to the epithelium, resulting in decreased cellular permeability. Because water permeability must first be established in the inner nucleus to transport small molecules toward the epithelium, the decrease in unmodified AQP5 abundance has putative functional consequence on the net current of water and small molecules throughout the lens. The net effect of AQP5- relative to AQP0-mediated water permeability in the inner nucleus has not been established, but AQP5 degradation may result in accumulation of small molecules including calcium and oxidized GSH. Ultimately, this may result in further proteostatic and homeostatic stress, leading to eventual ARNC formation.

## Summary

In summary, we demonstrate that DIA identifies more proteins in the lens than previously possible and delineates age-related changes in defined spatiotemporally distinct regions. Novel to this study, we show that a proteome remodeling event occurs at 50 years of aging and that oxidative stress response networks are retained with age, whereas cell–cell contacts are degraded with age. Expanding on this, we demonstrate the first proteomic identification of multiple GSH and ascorbic acid transport proteins in the human lens. In addition to supporting prior measurements of the aging lens, we identified that calcium transporter SLC24A2 is less abundant after proteome remodeling, leading to its accumulation and potential inhibition of the MCS. Finally, we show that AQP5 is depleted in the inner nucleus of the lens, coincident with proteome remodeling; a finding with important physiological implications for MCS activity changes with age (Fig. 7). Further functional studies are needed to evaluate these hypotheses, especially related to calcium accumulation in the aging lens. In addition, it is desirable to further expand this approach to cataract lenses to clearly delineate changes that occur in ARNC lenses relative to old healthy lenses.

## Data Availability

Raw MS data, deconvoluted mzML files, DIA-NN–created .dia files, DIA-NN reports, and R script saved data are available on ProteomeXchange. These data can be accessed using the project accession number (ProteomeXchange identifier) PXD033722.

## Supplemental data

This article contains supplemental data.

## Conflict of interest

The authors declare no competing interests.
