# Supplementary material for: Proteome Remodeling of the Eye Lens at 50 Years Identified With Data-Independent Acquisition
Source: Mol Cell Proteomics. 2022 Dec 5;22(1):100453. doi: 10.1016/j.mcpro.2022.100453 (PMC9800634; doi:10.1016/j.mcpro.2022.100453)
Supplement: Supplemental figures [file mmc1.docx]

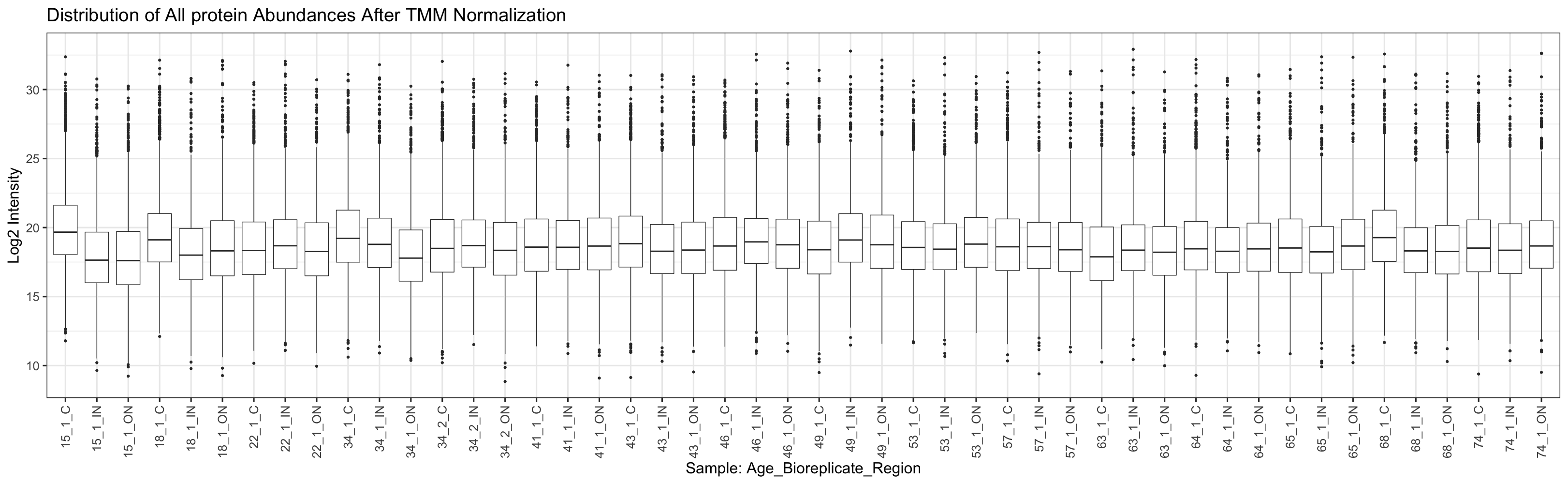


Supplemental Figure S1 - Distribution of protein groups after TMM normalization was performed on all protein groups measured in every sample. Deviation from population distributions is indicative of representative abundance change for protein groups not measured in all samples


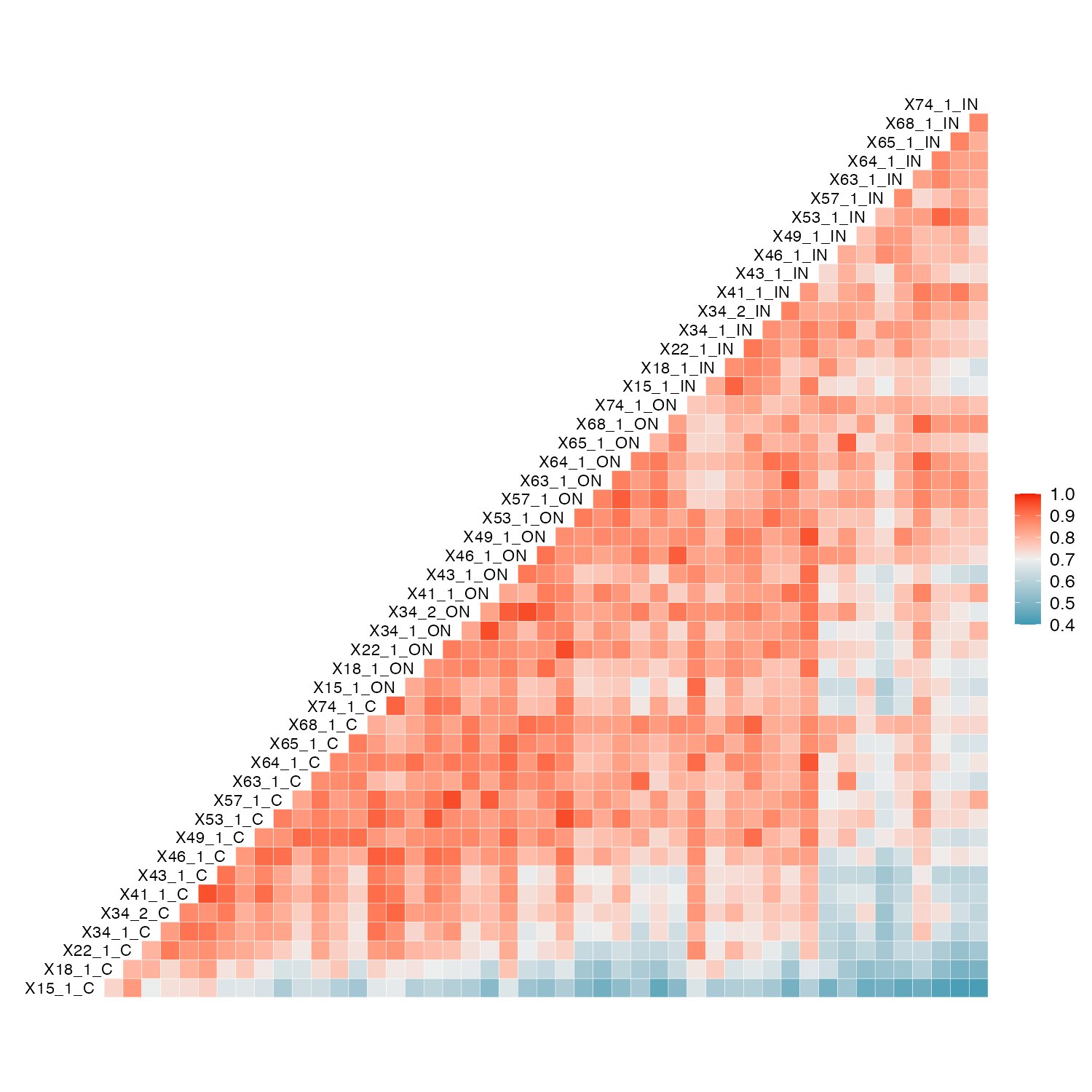


Supplemental Figure S2 – Spearman rank correlation plot of all 48 samples, demonstrating that samples within lens regions are approximately similar and correlation decreases concurrent to increasing difference in age between paired samples. Sample naming scheme is as follows X + subject age + subject age biological replicate + lens region (C = Cortex, ON = Outer Nucleus, IN= Inner Nucleus). Samples are grouped by fiber cell population and ordered by subject age.


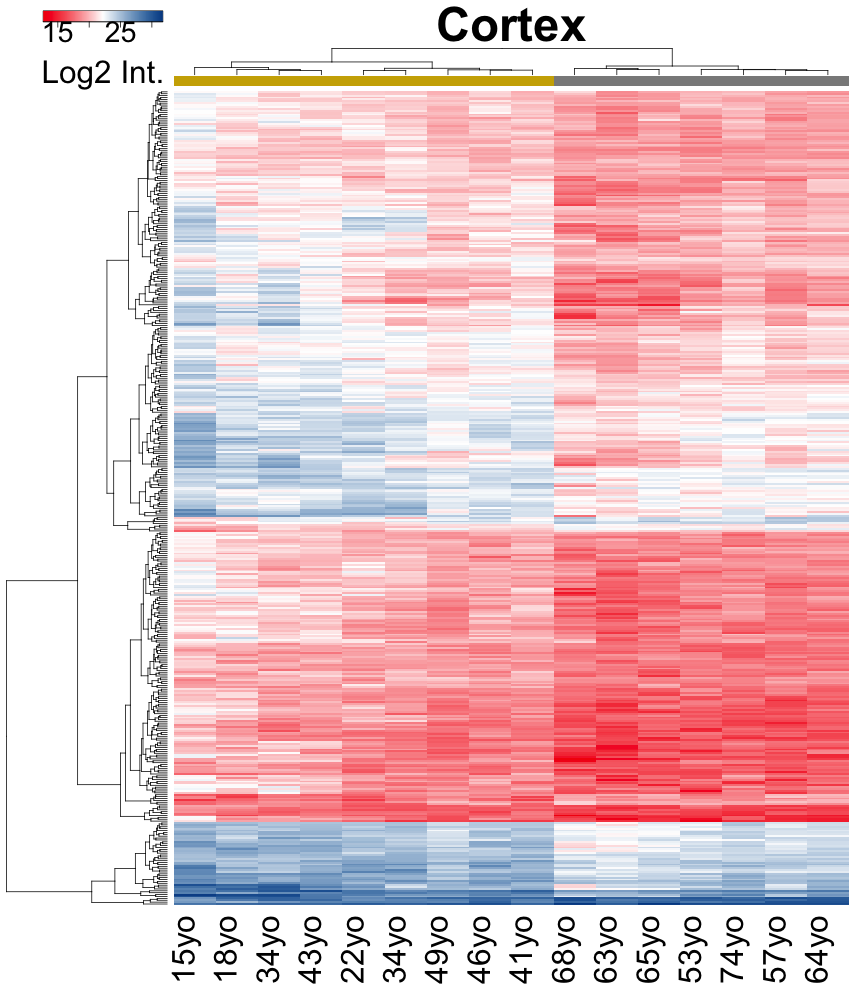


Supplemental Figure S3 - Full size graphic of hierarchical clustering plot visualized in Figure 2B with column subject age annotation. Gold column annotation for young samples (<50 years old), Silver column annotation for old samples (>50 years old)


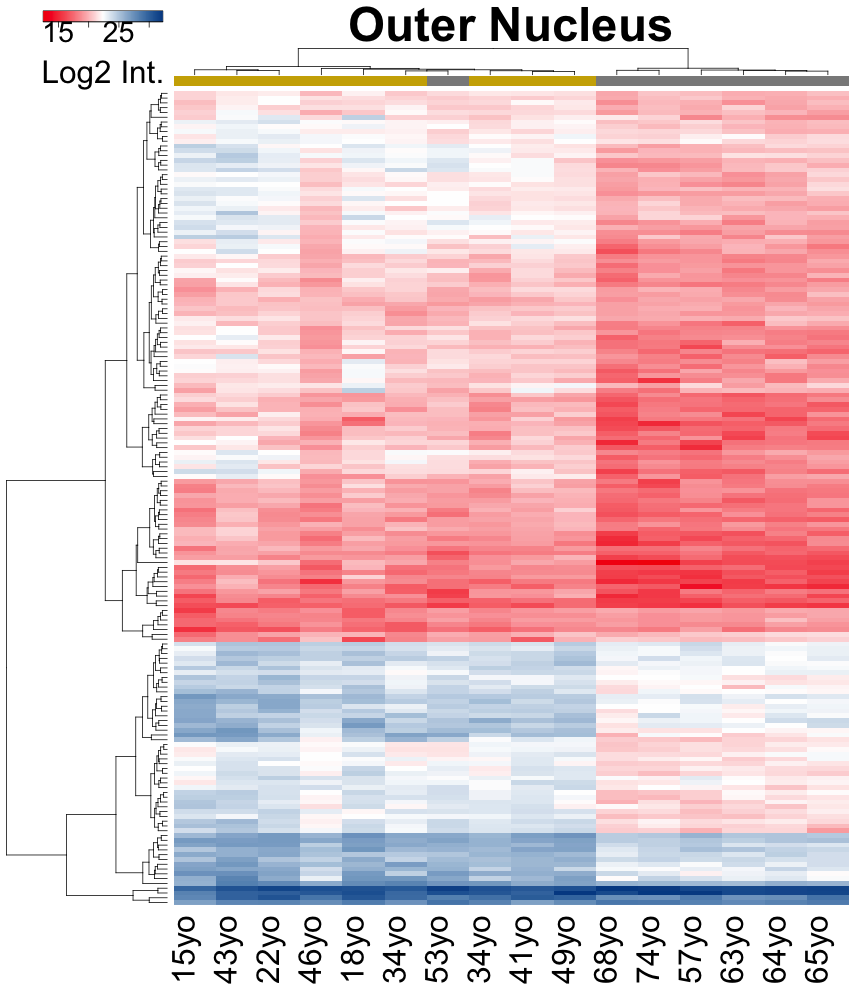


Supplemental Figure S4 - Full size graphic of hierarchical clustering plot visualized in Figure 2C with column subject age annotation. Gold column annotation for young samples (<50 years old), Silver column annotation for old samples (>50 years old)


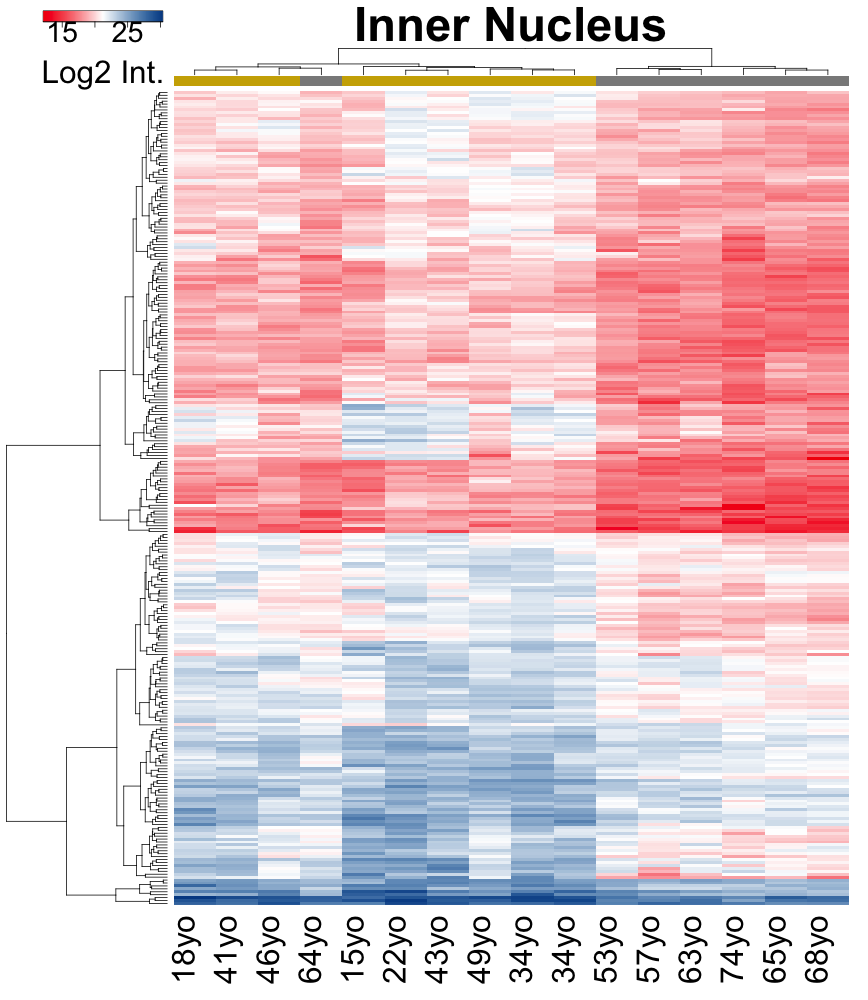


Supplemental Figure S5 - Full size graphic of hierarchical clustering plot visualized in Figure 2D with column subject age annotation. Gold column annotation for young samples (<50 years old), Silver column annotation for old samples (>50 years old)


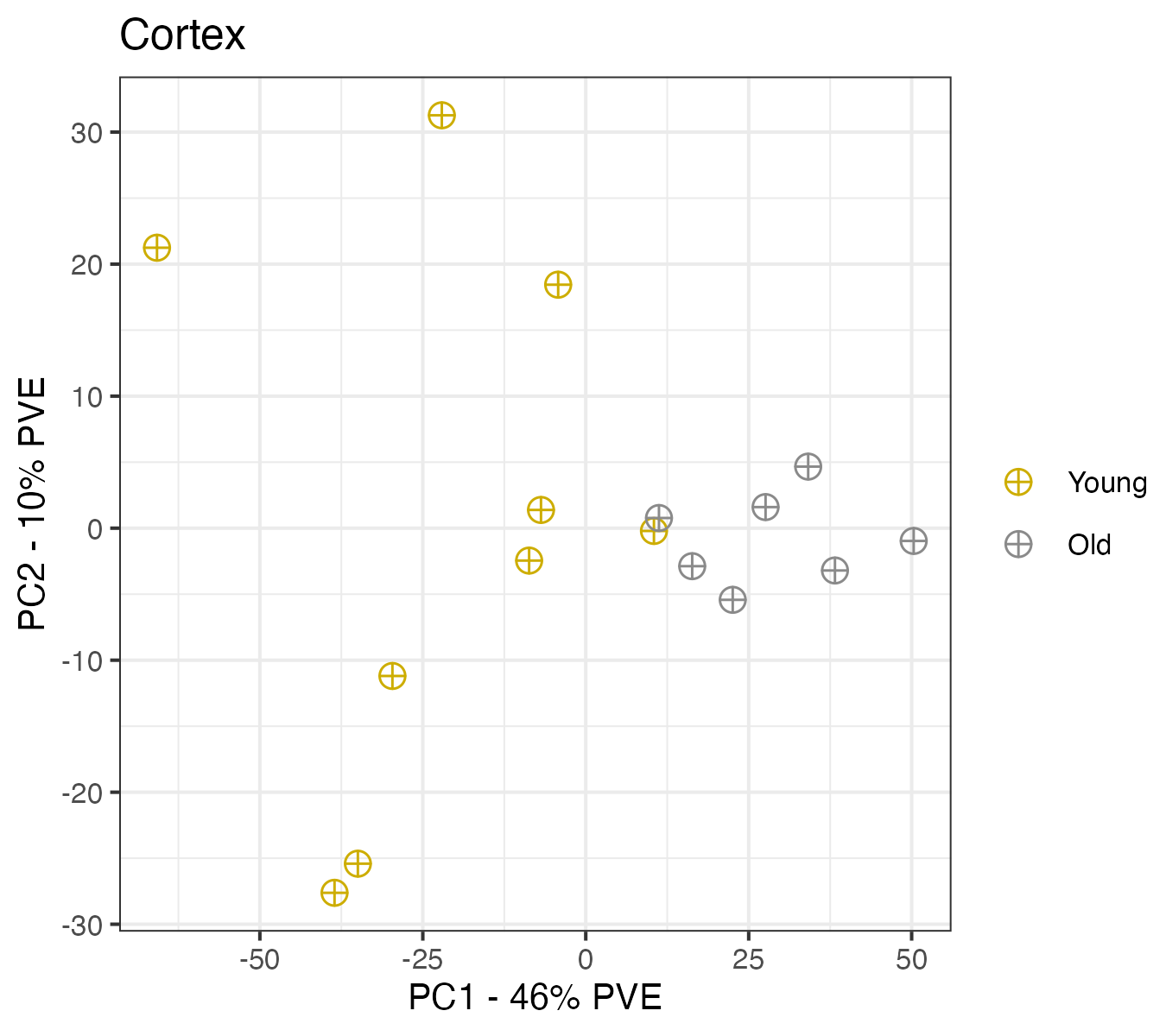


Supplemental Figure S6 - PCA plot colored by age group demonstrates separation of young and old fiber cell populations on PC1 based on protein groups identified in all 16 samples (n=1,429). Proteins that significantly contribute to the negative (younger) loading on PC1 include epoxide hydrolase, fibrillin-1, phosphate carrier protein, cytochrome c oxidase subunit 2, and tubulin beta-4A chain. Loadings that most significantly contribute to the positive (older) loading on PC1 include γB-crystallin, WD repeat-containing protein 25, 4-hydroxyphenylpyruvate dioxygenase, and glutamate synthesis enzyme kynurenine oxoglutarate transaminase.


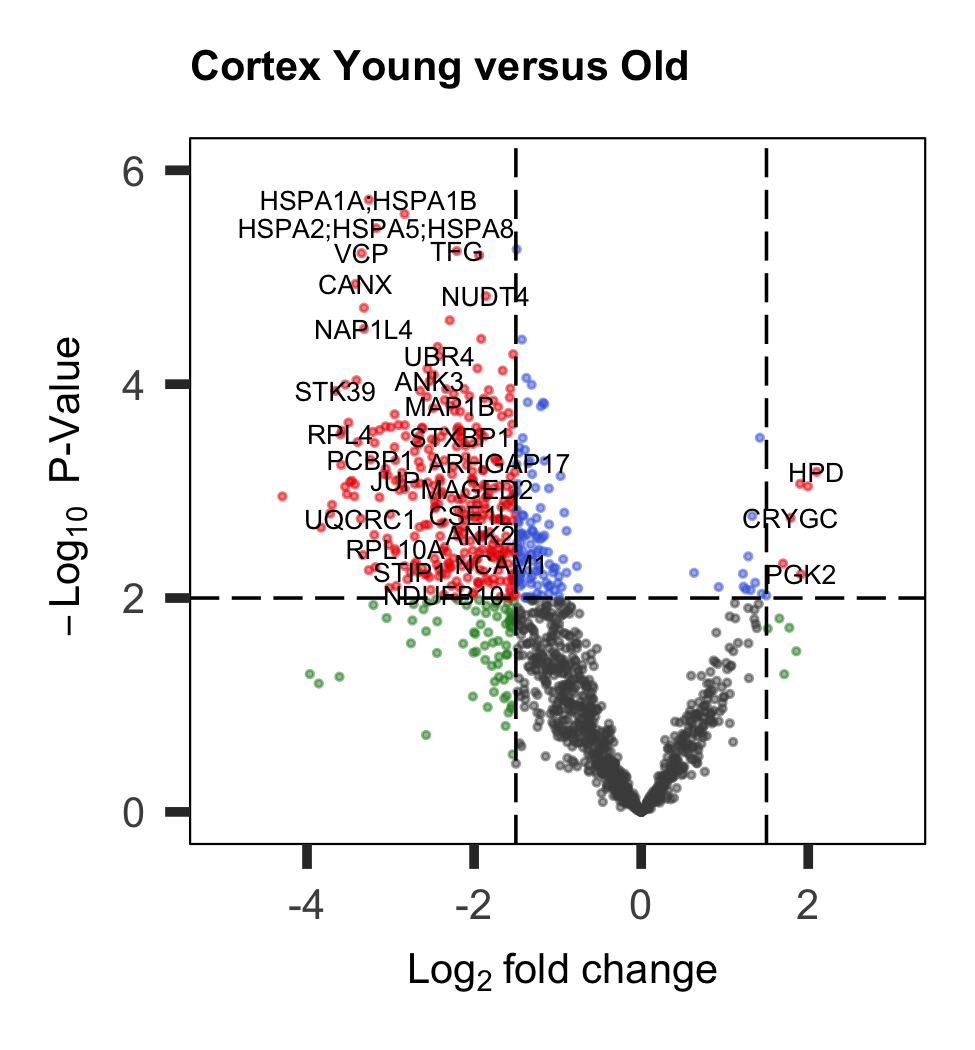


Supplemental Figure S7 - Volcano plot of preferentially retained or degraded proteins with significance cutoffs of 0.01 unmoderated p-value and 1.5 log2 fold change. UniProt identifiers converted to gene names. The statistical power of this test is estimated to be approximately 0.65 with a sample size of 7, the size of the old lens sample group, and at 5% FDR.


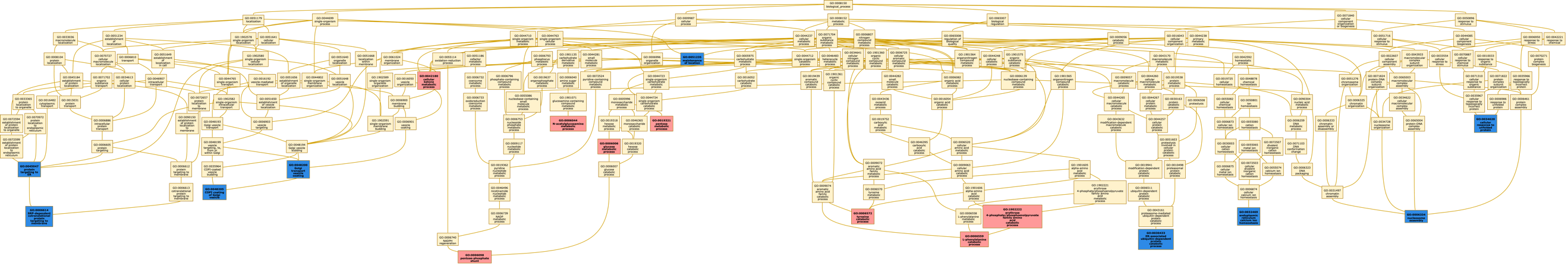


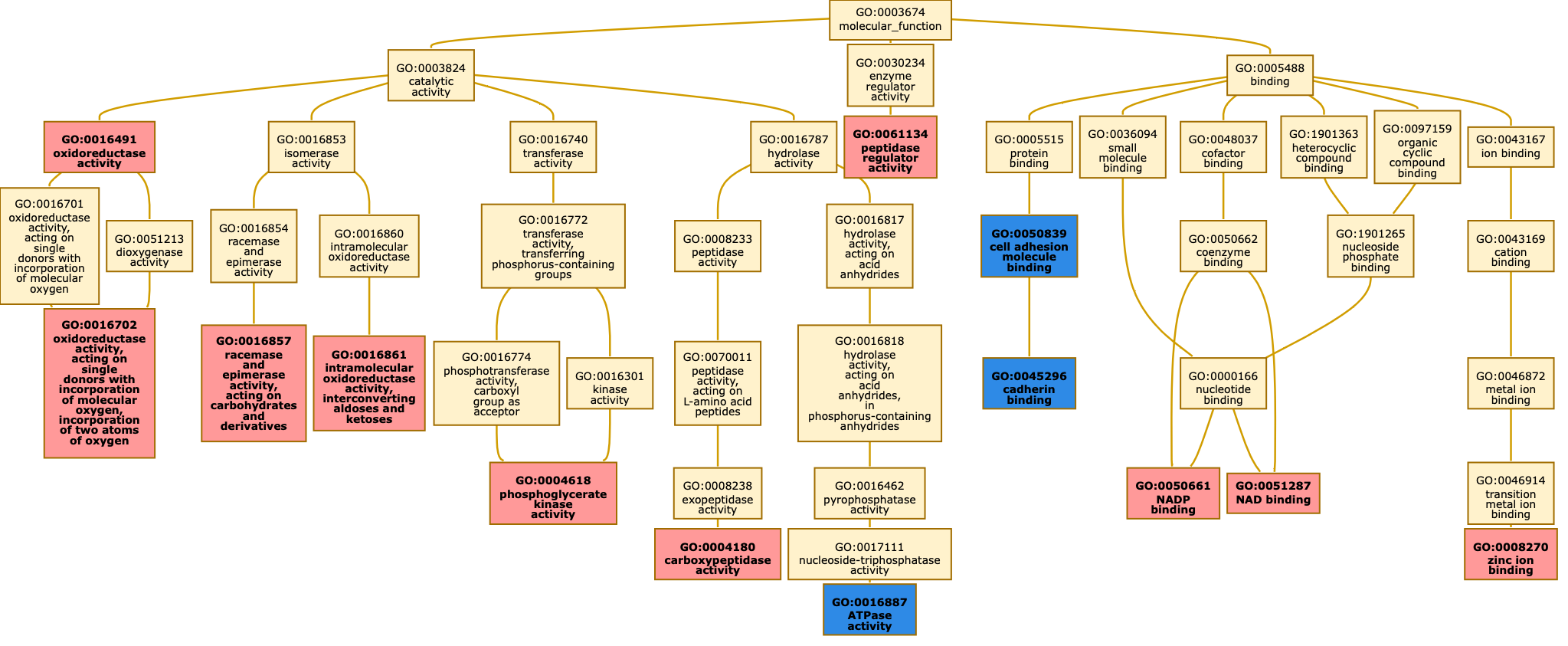

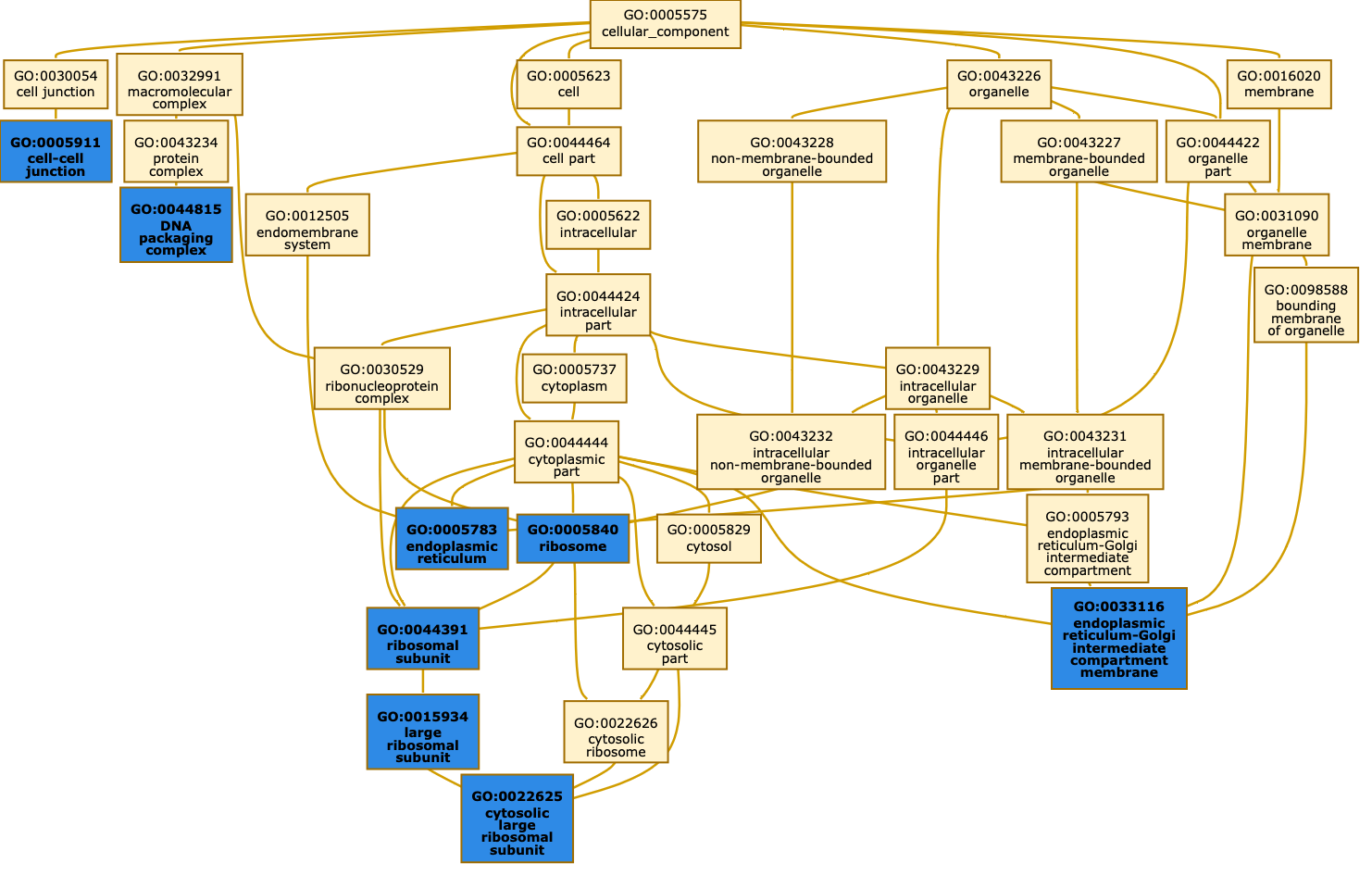


Supplemental Figure S8 - GO network relationship directed acyclic graphs of cortex ontology enrichment sets. Top) GO Biological Product, Middle) GO Molecular Function, Bottom) GO Cellular Component. Young lens terms are colored in blue and old lens enrichment terms in red.


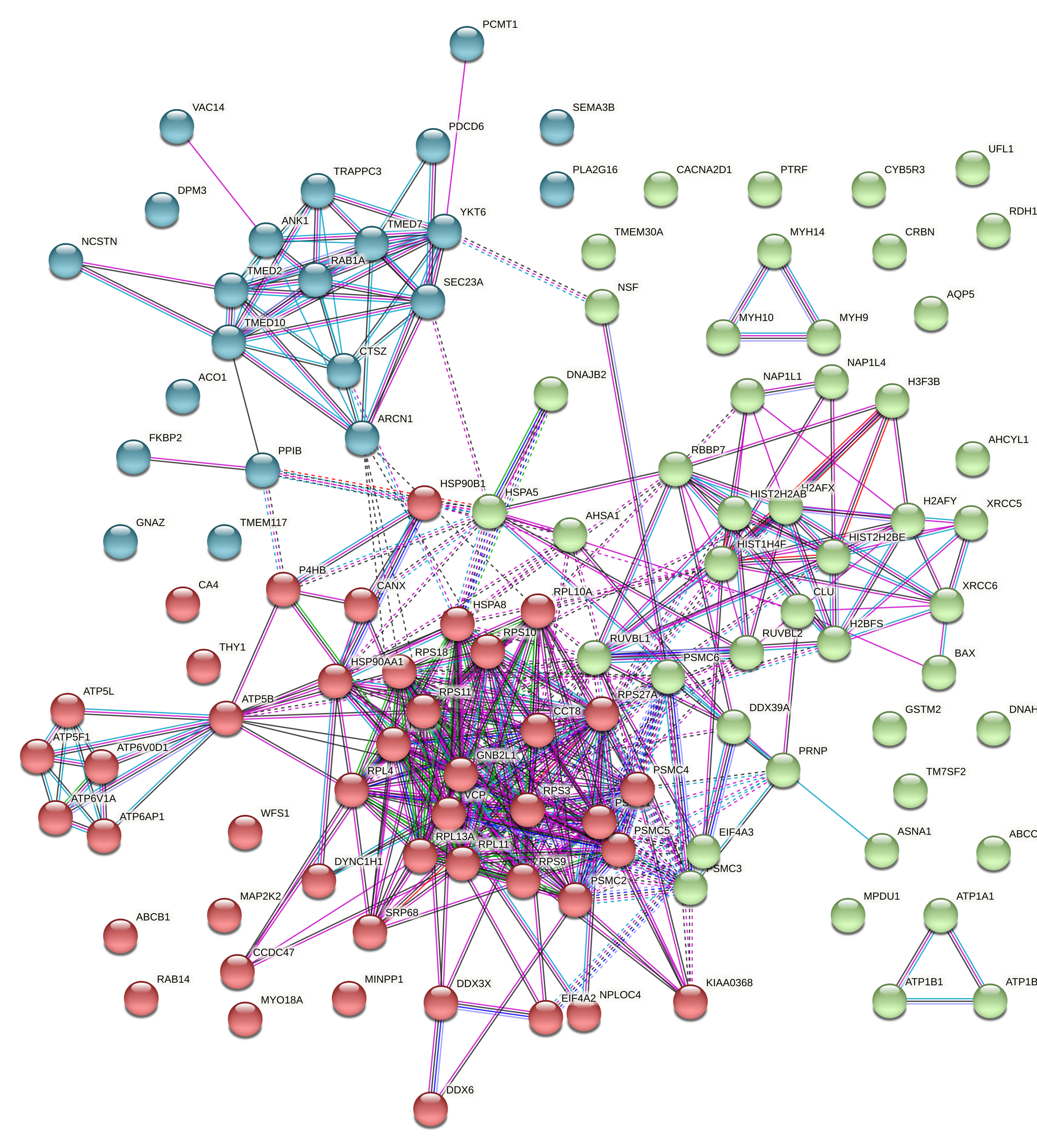


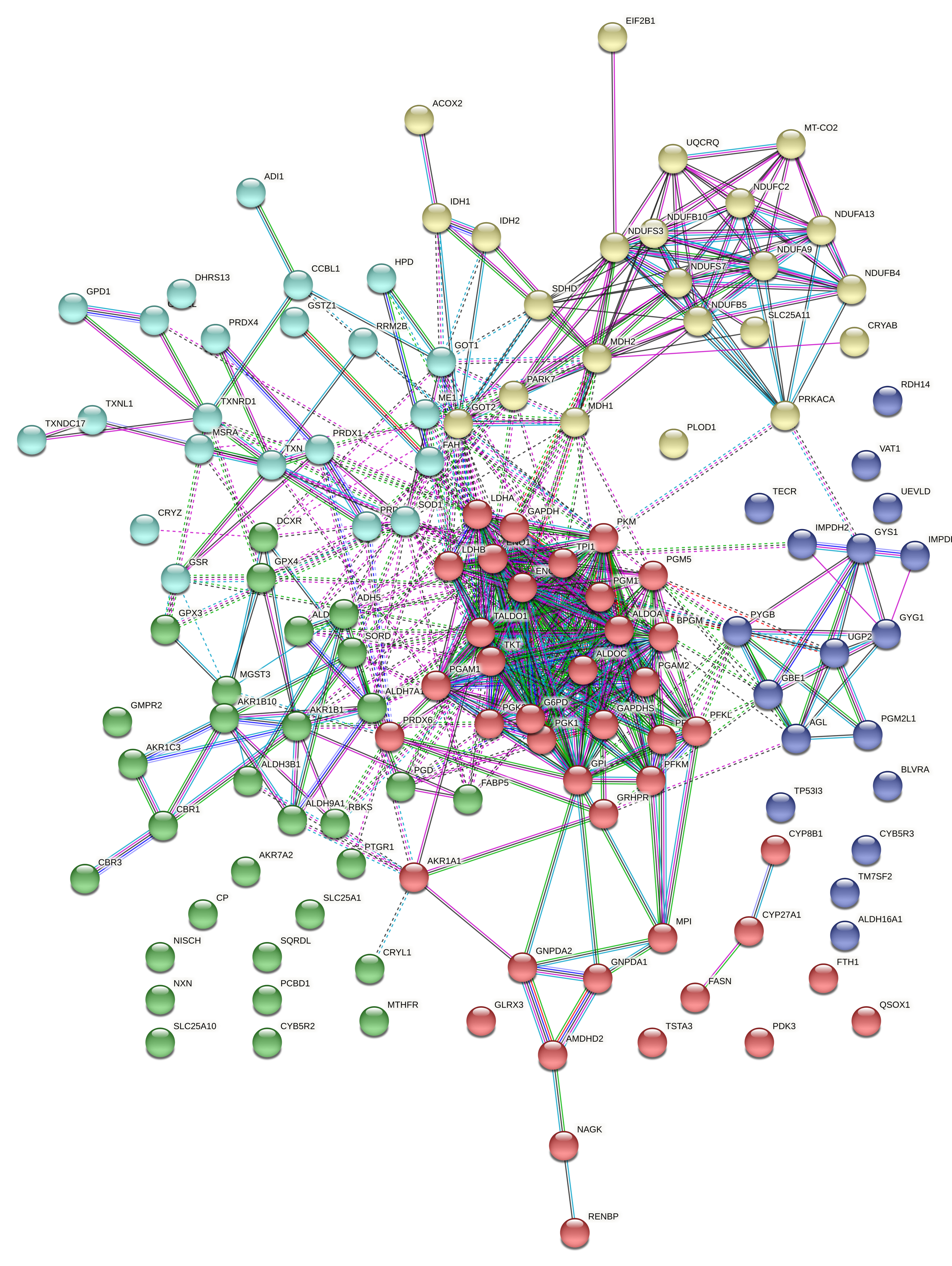


Supplemental Figure S9 - STRING Protein-Protein Interaction prediction network. Top) Proteins associated with young over old enriched ontologies in Figure 3, Bottom) Proteins associated with old over young enriched ontologies in Figure 3. Colored by K-Nearest Neighbors.


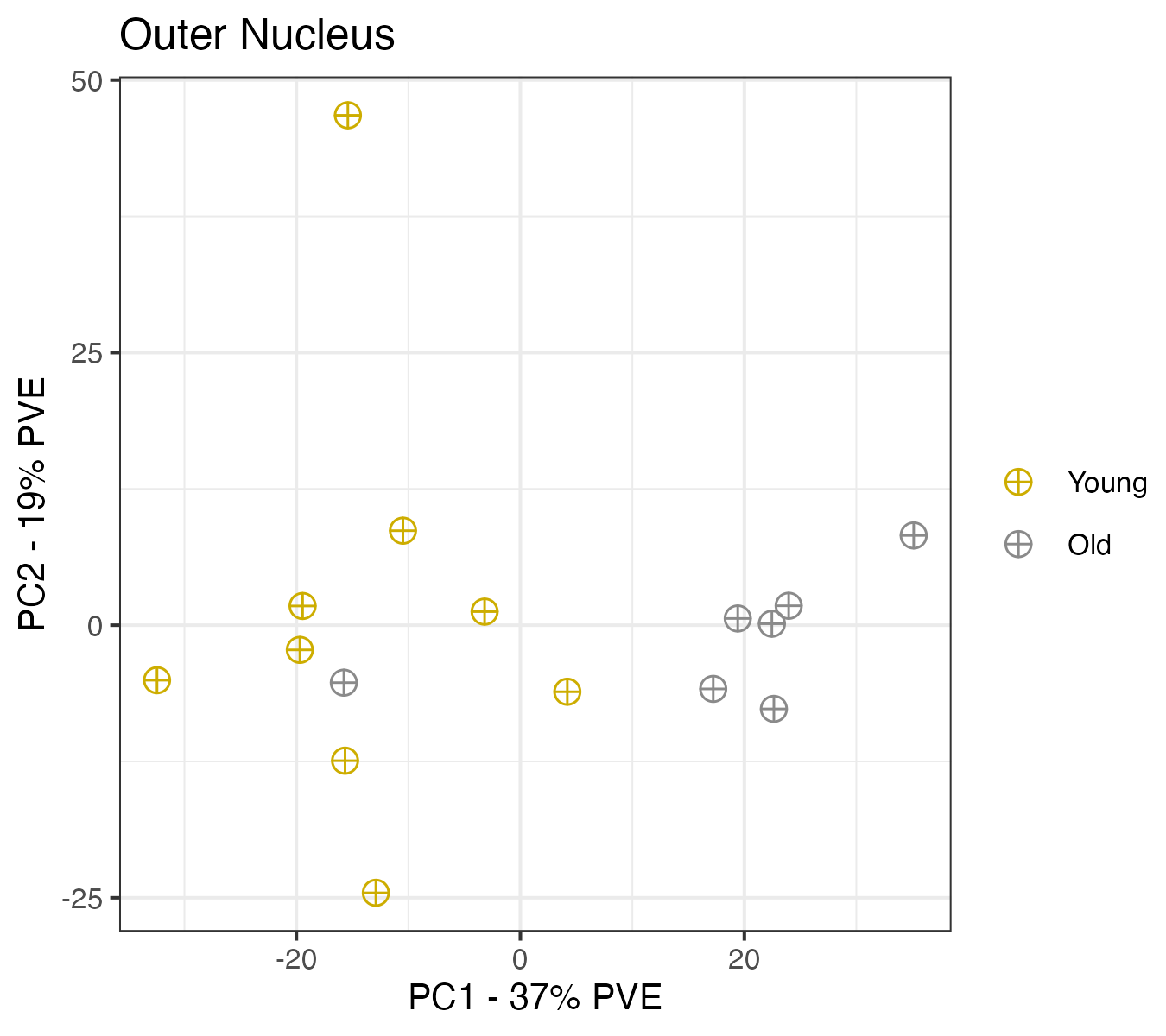


Supplemental Figure S10 - PCA plot colored by age group demonstrates separation of young and old fiber cell populations on PC1 based on protein groups identified in all 16 samples (n=1,114). Cell adhesion molecule 3, guanine nucleotide binding protein, insulin-like growth factor-binding protein 7, and BASP1 are protein groups associated with negative, young PC1 protein loadings. Protein phosphatase-1 regulatory subunit 15a, hspB3, βB1-crystallin and WD repeat-containing protein 25 are associated with positive, old PC1 protein loadings. No clear trends in biology emerge from these results aside from consistent young-lens loading annotation of BASP1 and old-lens βB1-crystallin and WD repeat-containing protein 25.


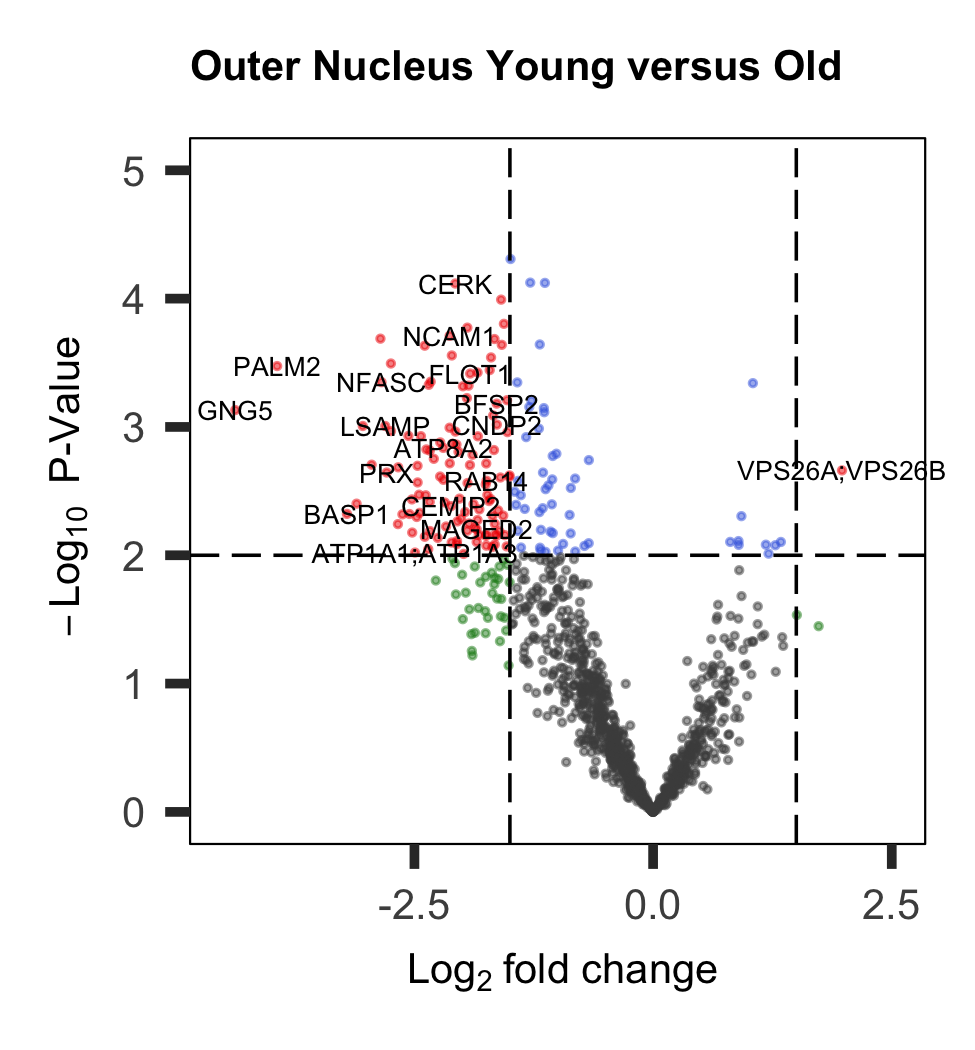


Supplemental Figure S11 - Volcano plot of Outer Nucleus preferentially retained or degraded proteins with significance cutoffs of 0.01 unmoderated p-value and 1.5 log2 fold change. UniProt identifiers converted to gene names. The statistical power of this test is estimated to be approximately 0.6 with a sample size of 7, the size of the old lens sample group, and at 5% FDR.


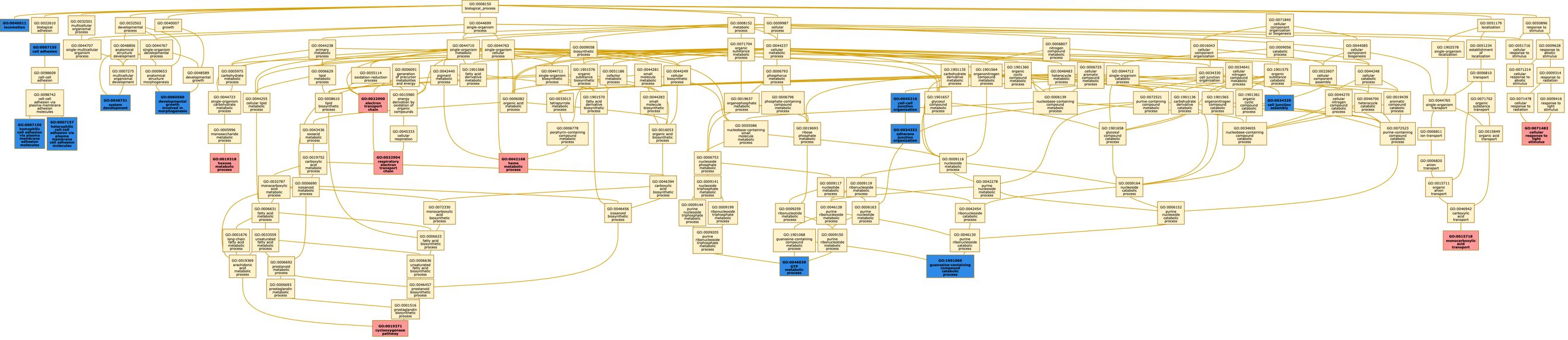


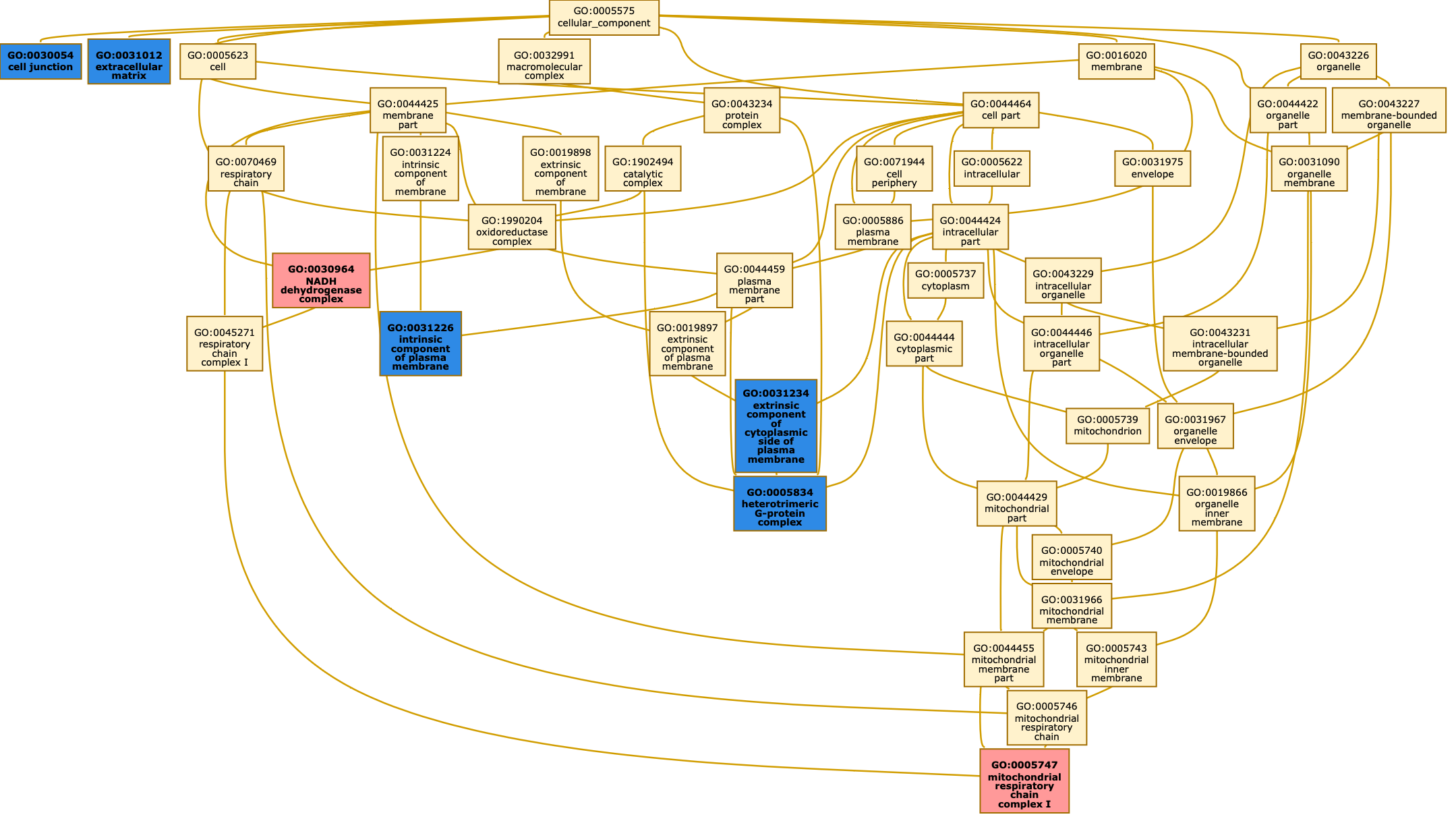

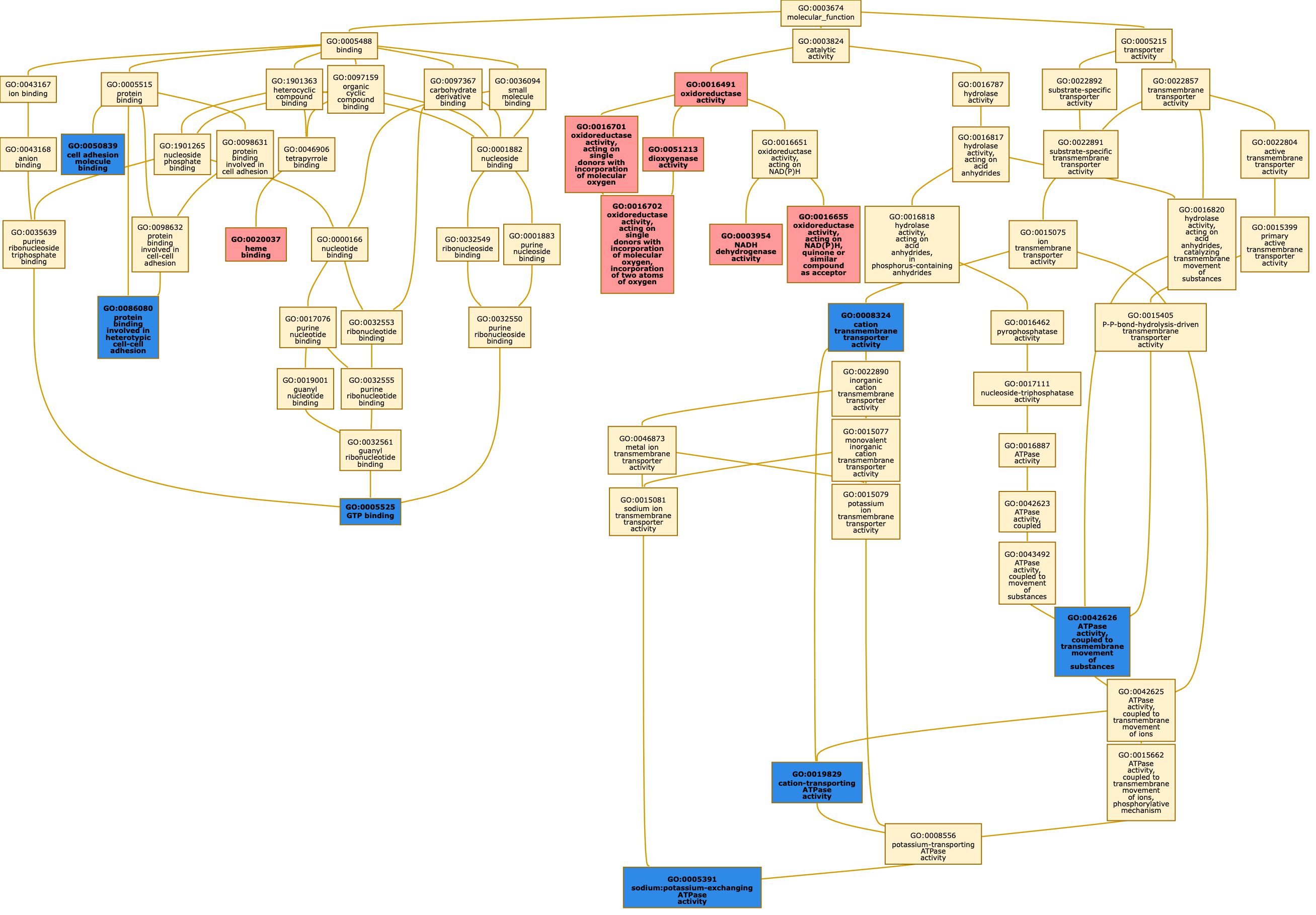


Supplemental Figure S12 - GO network relationship directed acyclic graphs of Outer Nucleus ontology enrichment sets. Top) GO Biological Product, Middle) GO Molecular Function, Bottom) GO Cellular Component. Young lens terms are colored in blue and old lens enrichment terms in red.


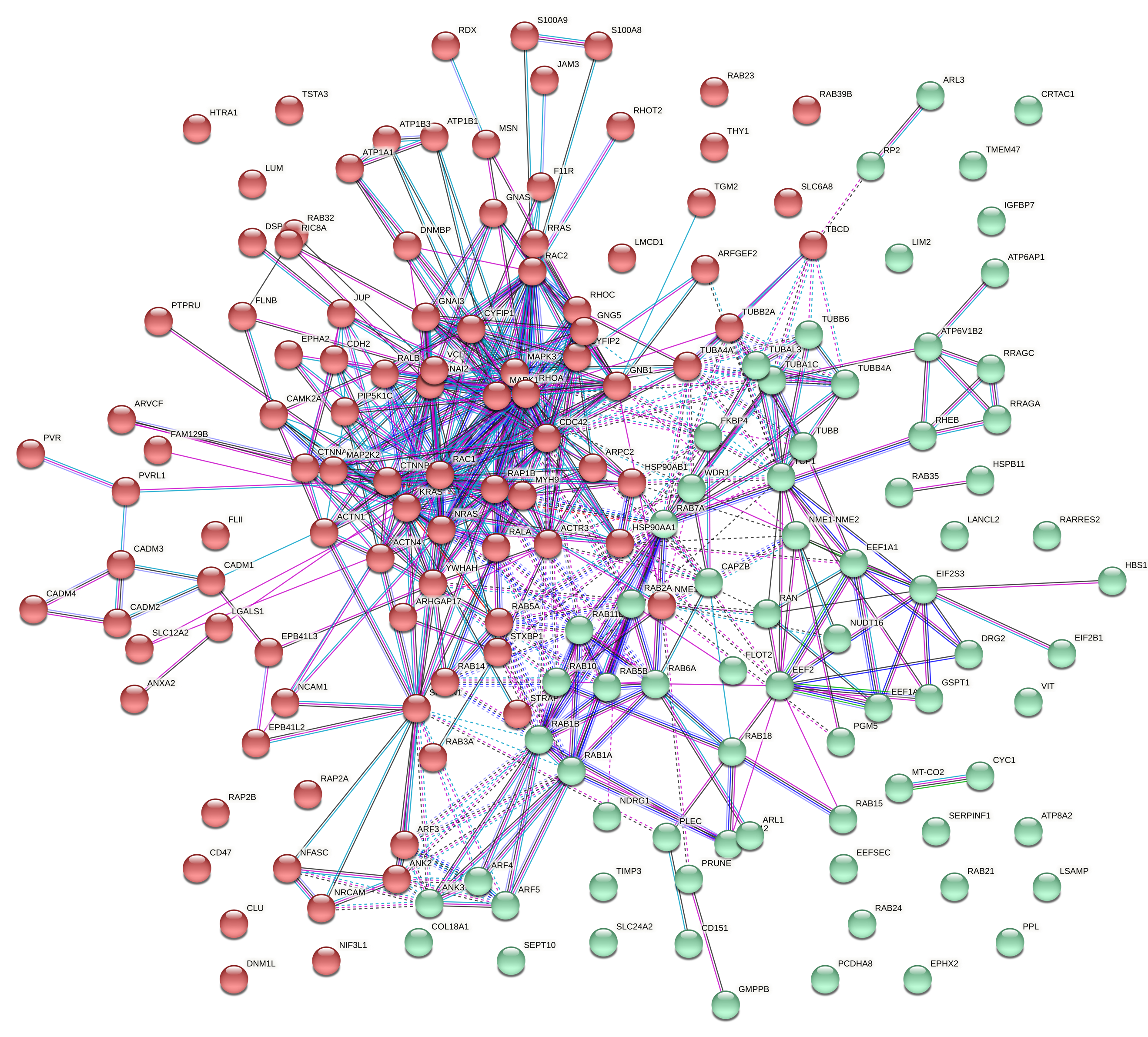


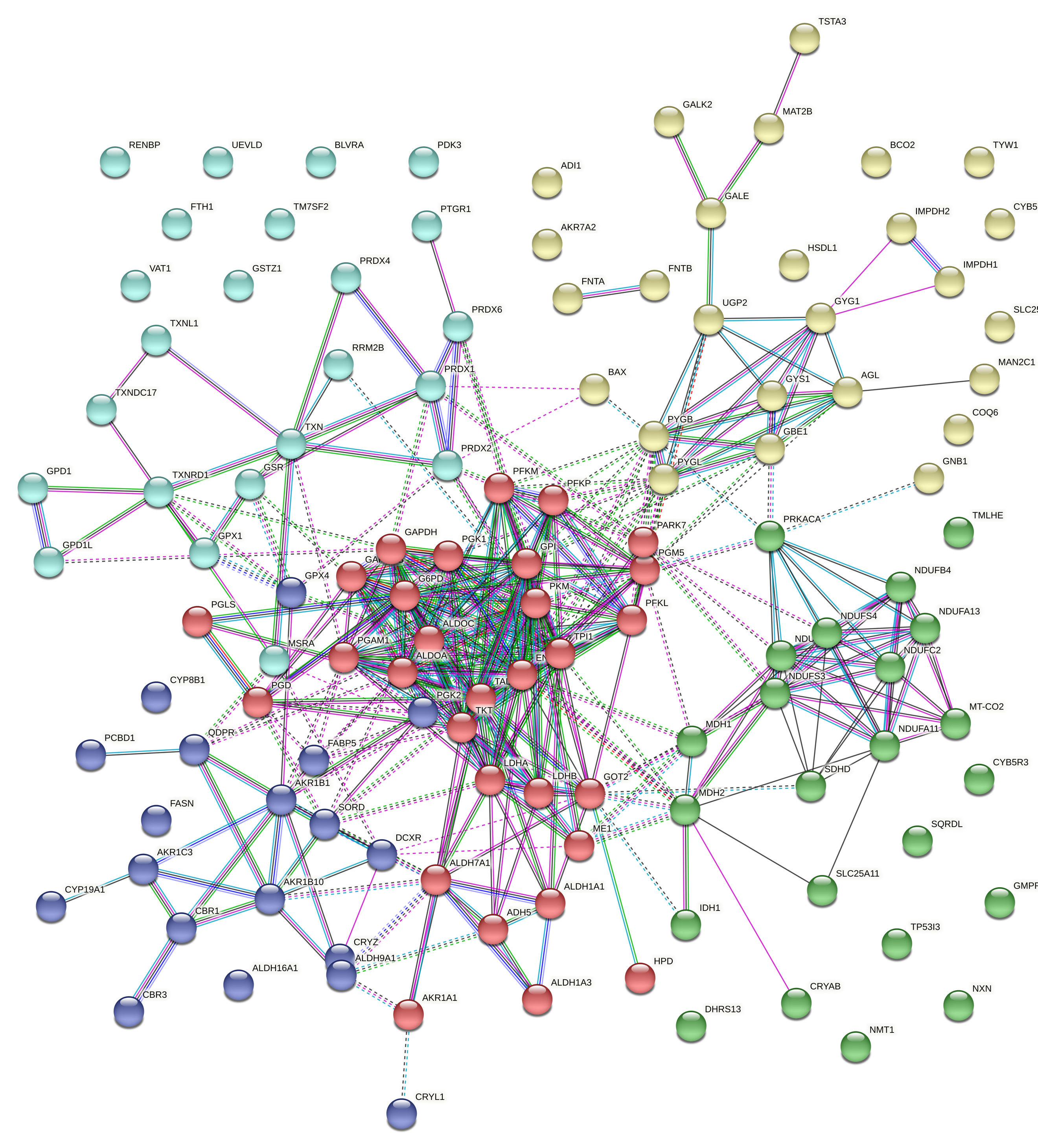


Supplemental Figure S13 - STRING Protein-Protein Interaction prediction network. Top) Proteins associated with young over old enriched ontologies in Figure 3, Bottom) Proteins associated with old over young enriched ontologies in Figure 4. Colored by K-Nearest Neighbors.


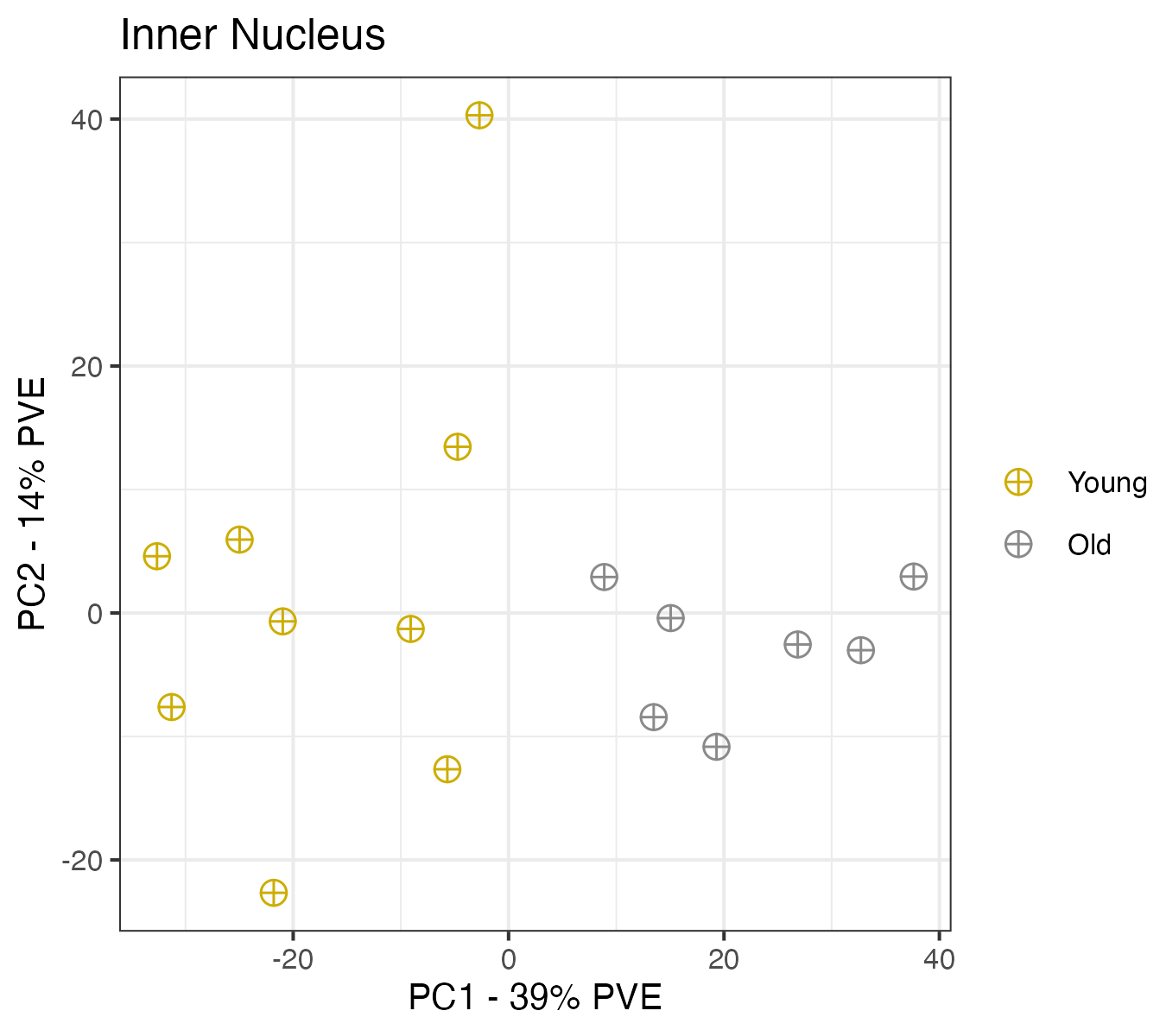


Supplemental Figure S14 - PCA plot colored by age group demonstrates separation of young and old fiber cell populations on PC1 based on protein groups identified in all 16 samples (n=969 PC1 protein loadings with the largest negative value include cell adhesion molecule 3, insulin-like growth factor binding protein 7, A-kinase anchor protein 2-related, and BASP1. Positive PC1 protein loadings included AP-1 complex subunit sigma 1a, protein phosphatase 1 regulatory subunit 15a, hspB3, βB1-crystallin, and vacuolar protein sorting-associated protein 26A.


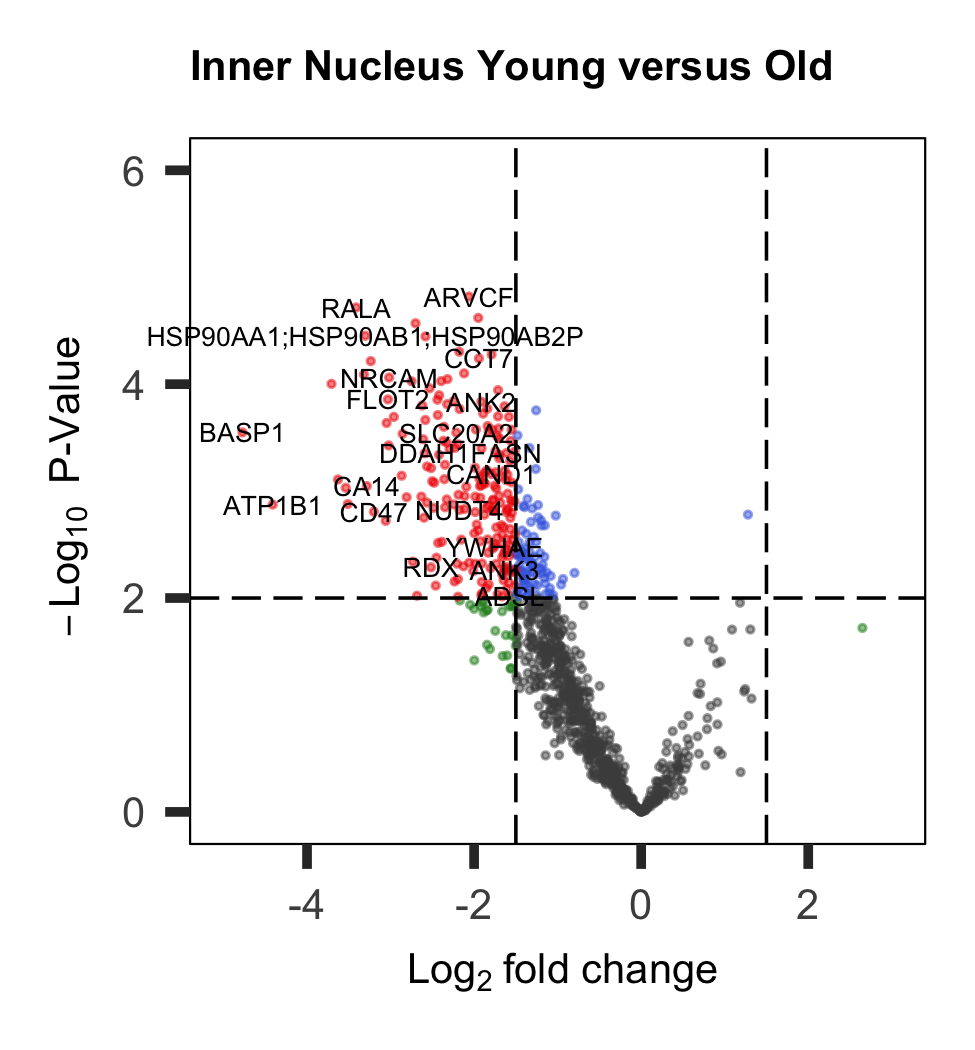


Supplemental Figure S15 - Volcano plot of Inner Nucleus preferentially retained or degraded proteins with significance cutoffs of 0.01 unmoderated p-value and 1.5 log2 fold change. UniProt identifiers converted to gene names. The statistical power of this test is estimated to be approximately 0.6 with a sample size of 7, the size of the old lens sample group, and at 5% FDR.


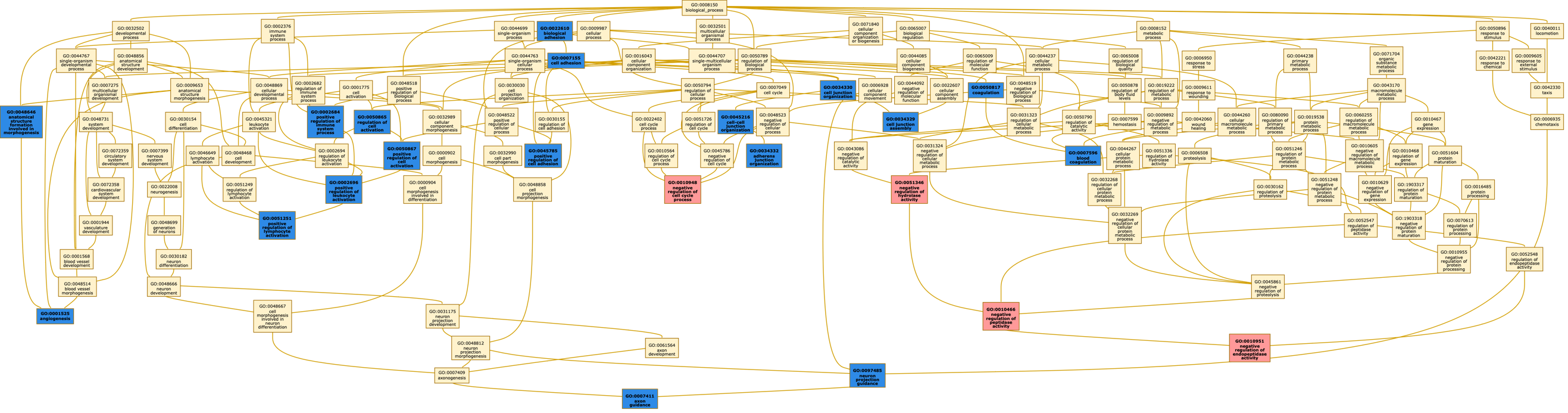

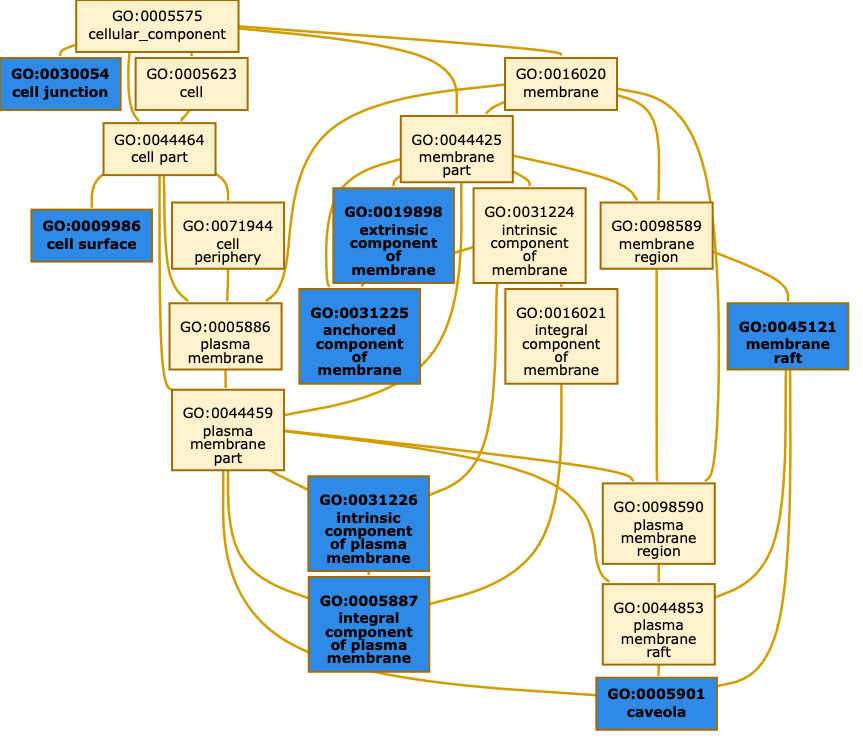

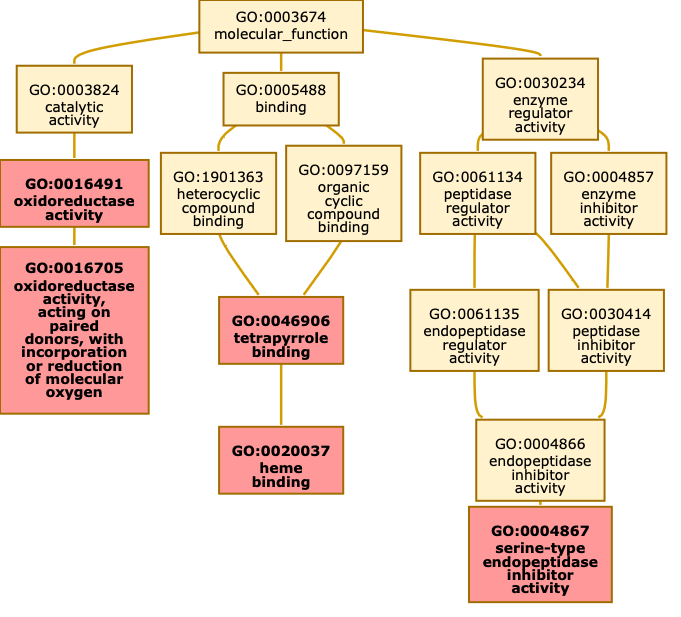


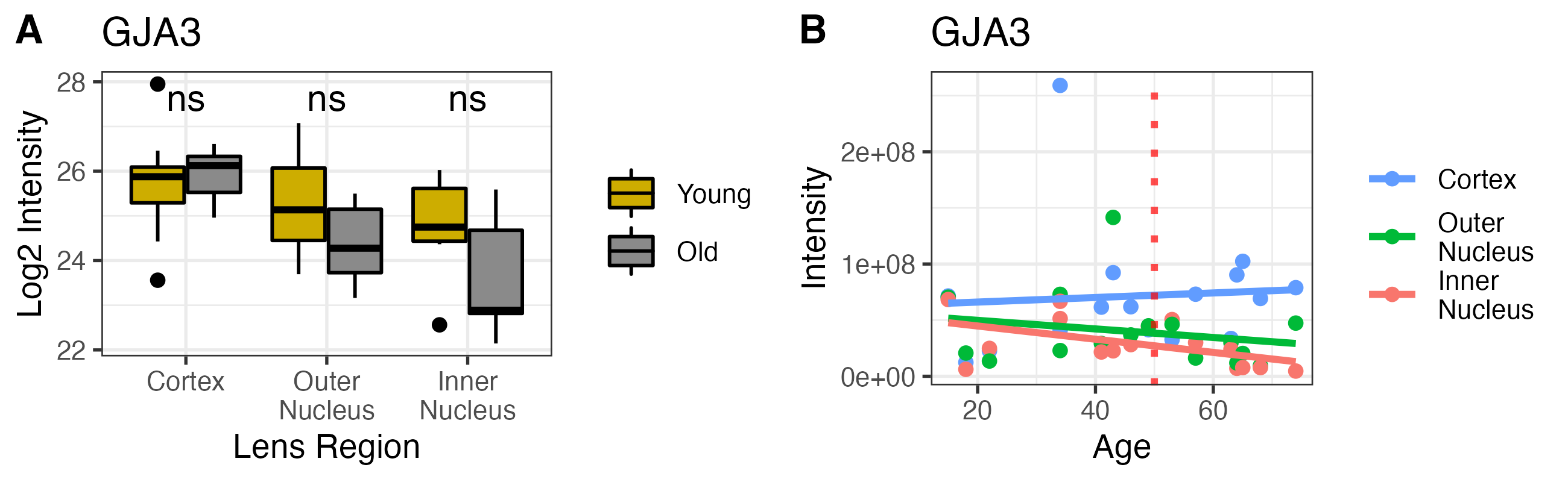


Supplemental Figure S16 - Abundance of Connexin 46 (GJA3) when deamidated residues are considered towards the total abundance of the protein group. When GJA3 is unmodified (Figure 6), a significant change is measured by two-sample t-test. A) When deamidation is included, no significance is established; B) There is a qualitative decline in GJA3 abundance in the outer and inner nucleus with age, but it is not consistent with the proteome remodeling event. T-test significance cutoffs were set at * = <0.05, ** = <0.01, *** = <0.001, **** =<0.0001

Supplemental Figure S17- GO network relationship directed acyclic graphs of Inner Nucleus ontology enrichment sets. Top) GO Biological Product, Middle) GO Molecular Function, Bottom) GO Cellular Component. Young lens terms are colored in blue and old lens enrichment terms in red.


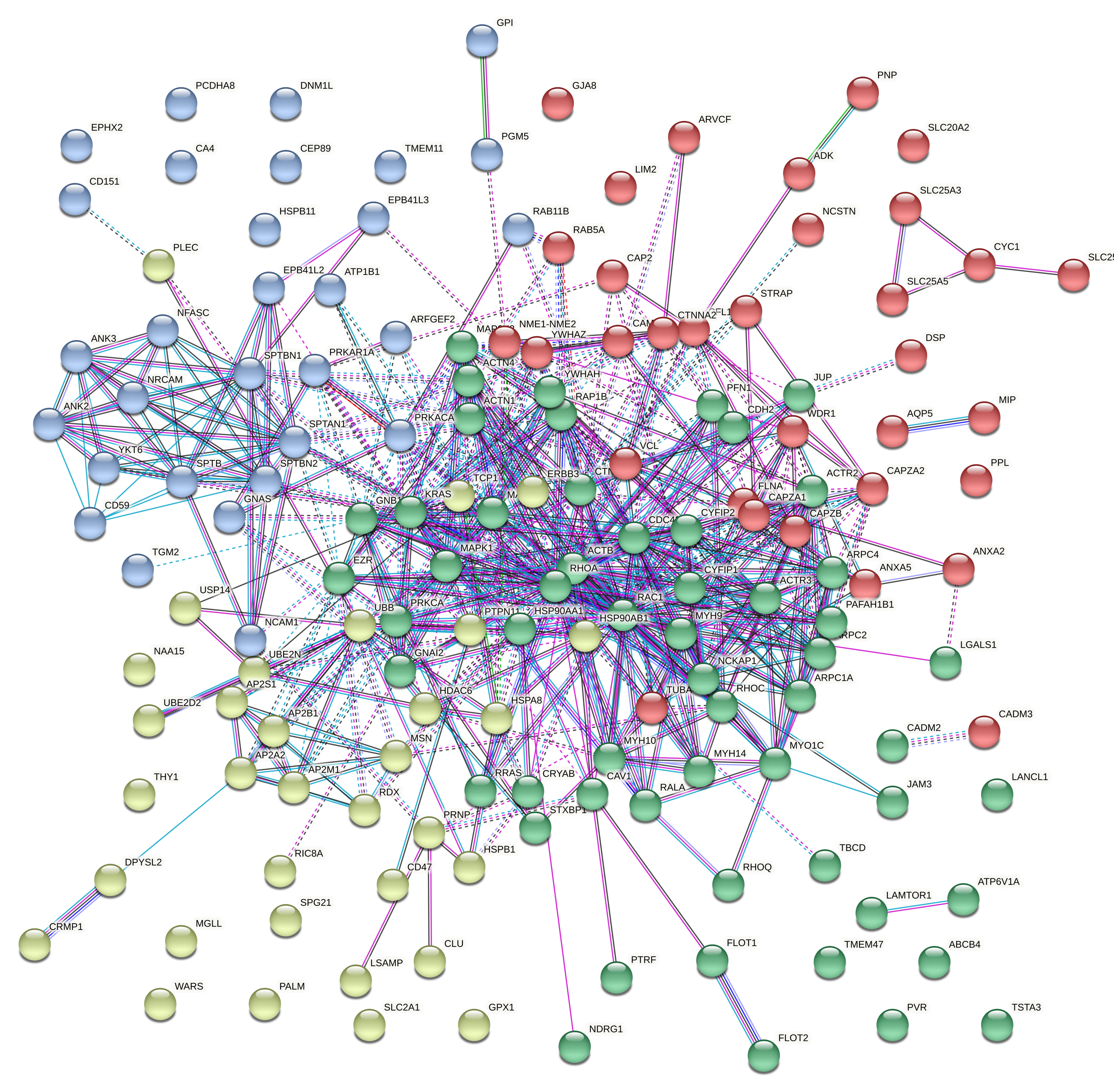


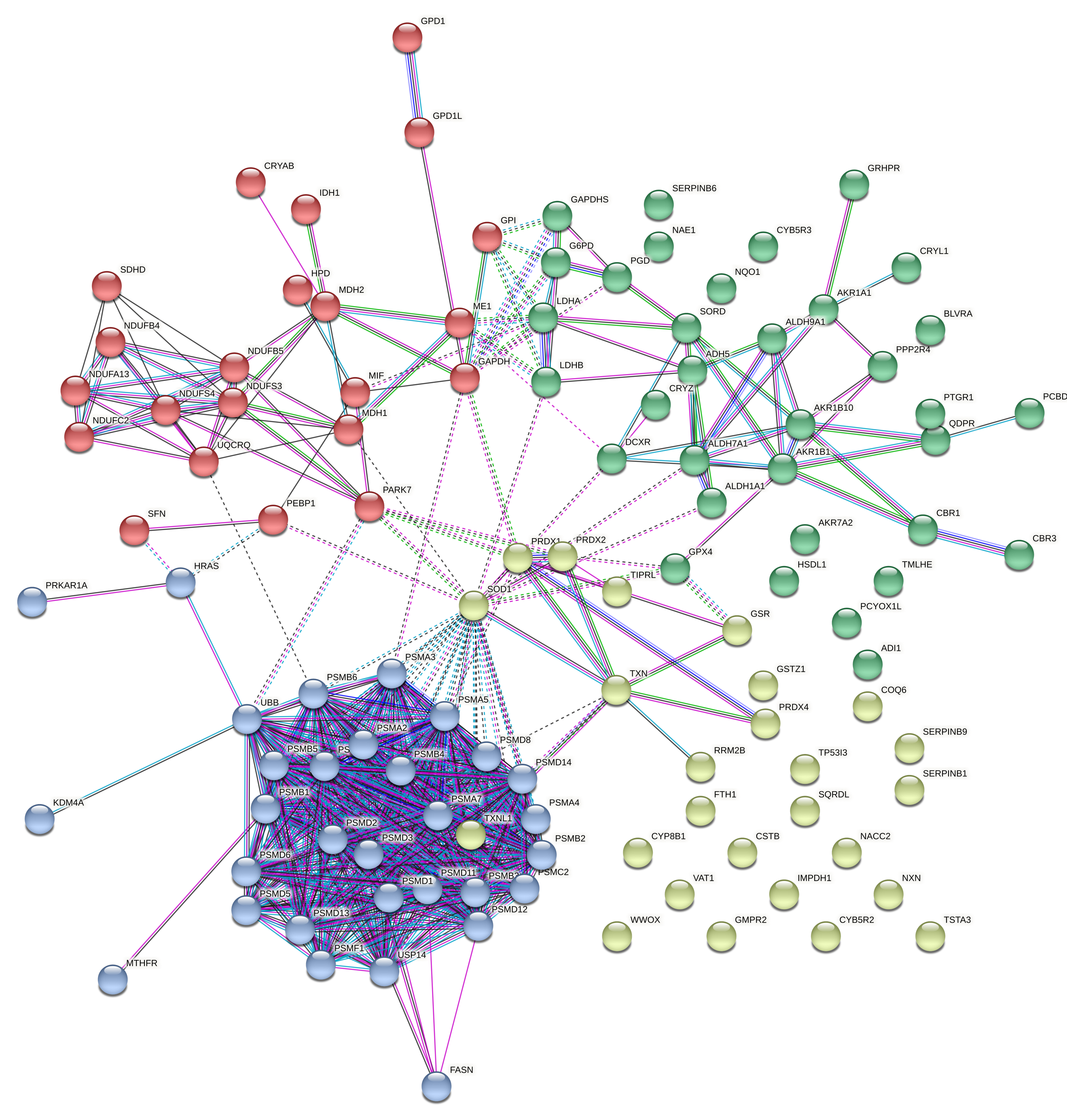


Supplemental Figure S18 - STRING Protein-Protein Interaction prediction network. Top) Proteins associated with young over old enriched ontologies in Figure 3, Bottom) Proteins associated with old over young enriched ontologies in Figure 4. Colored by K-Nearest Neighbors.


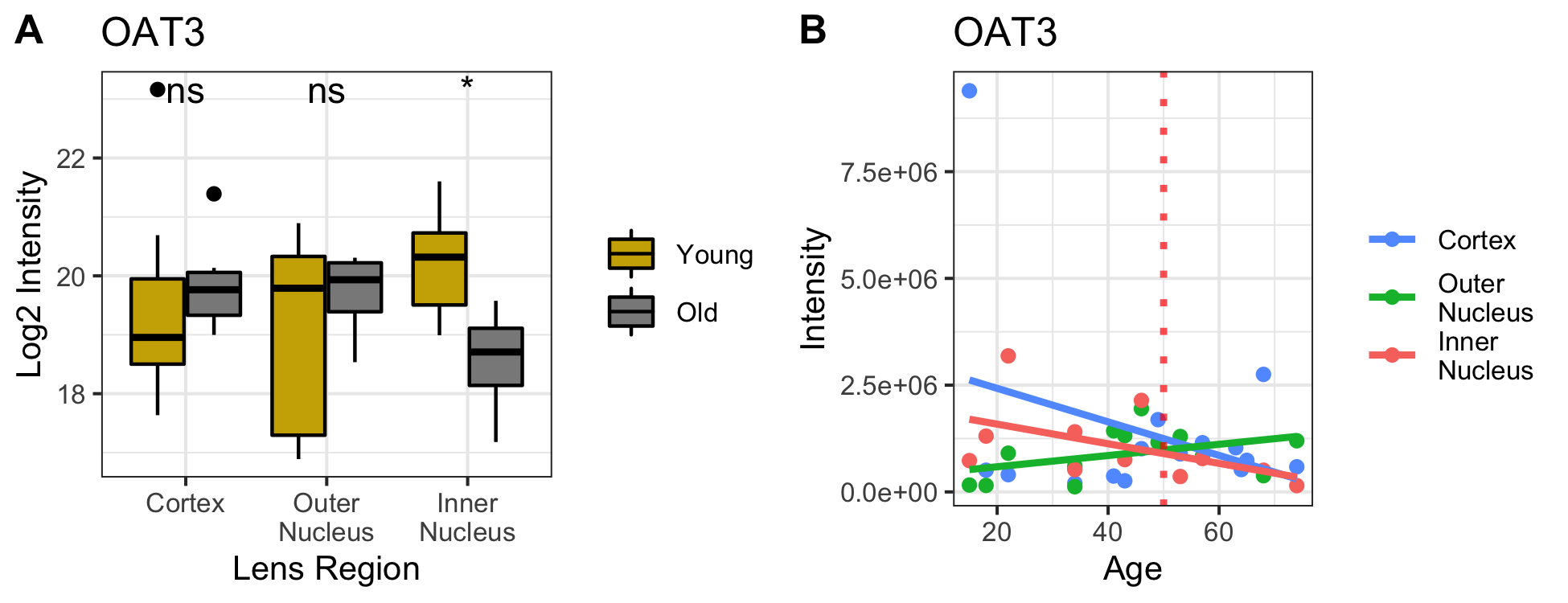

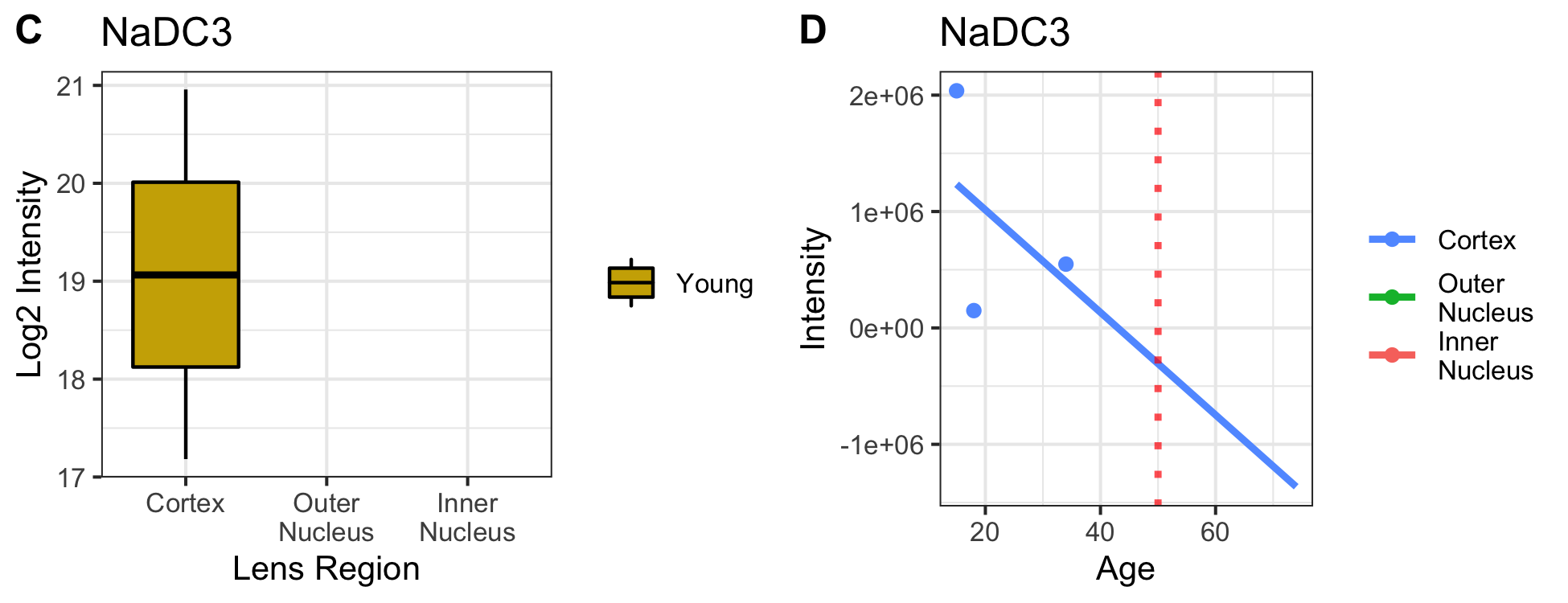

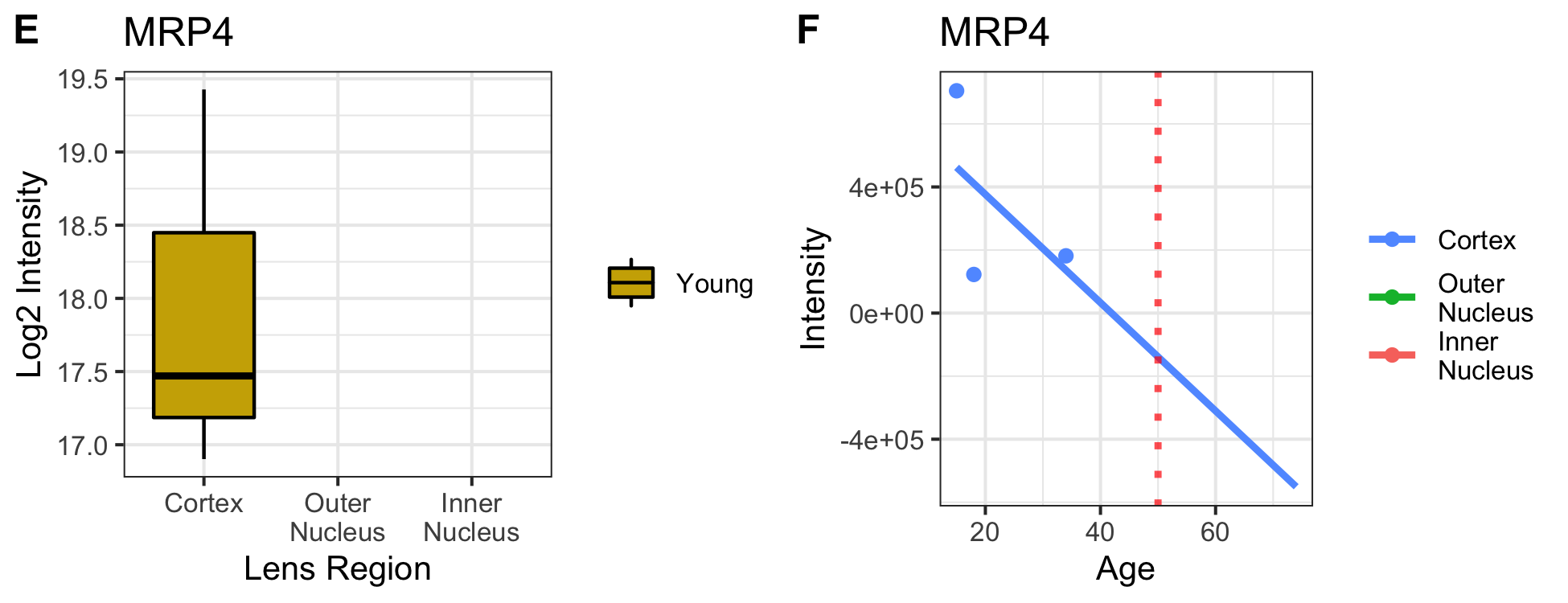

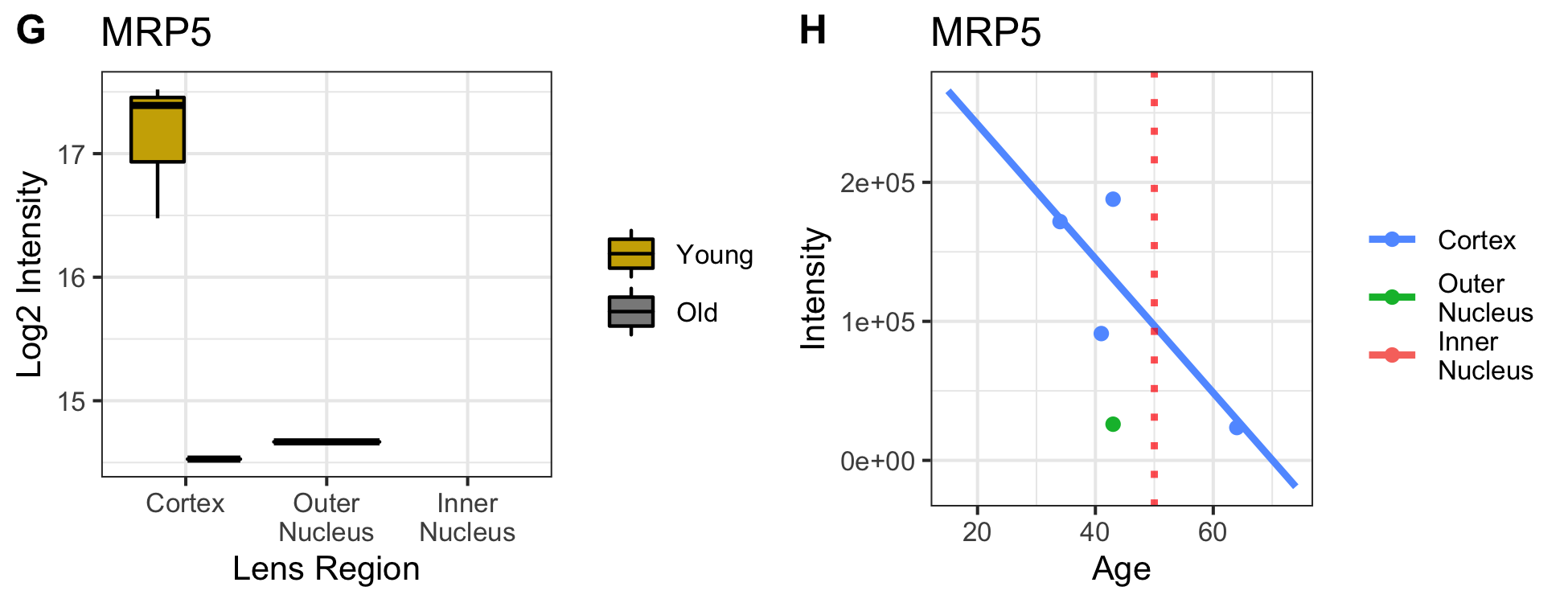


**C**

**E**

**F**

**G**

**H**

**K**

**J**


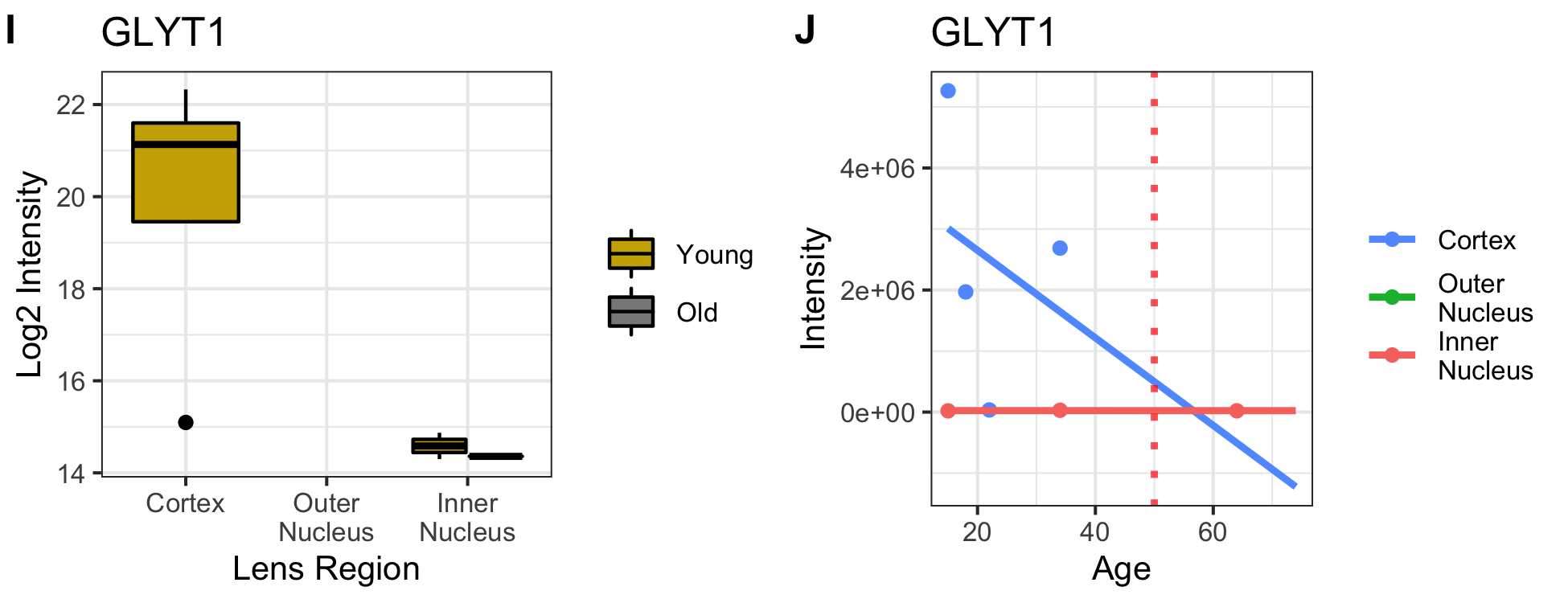

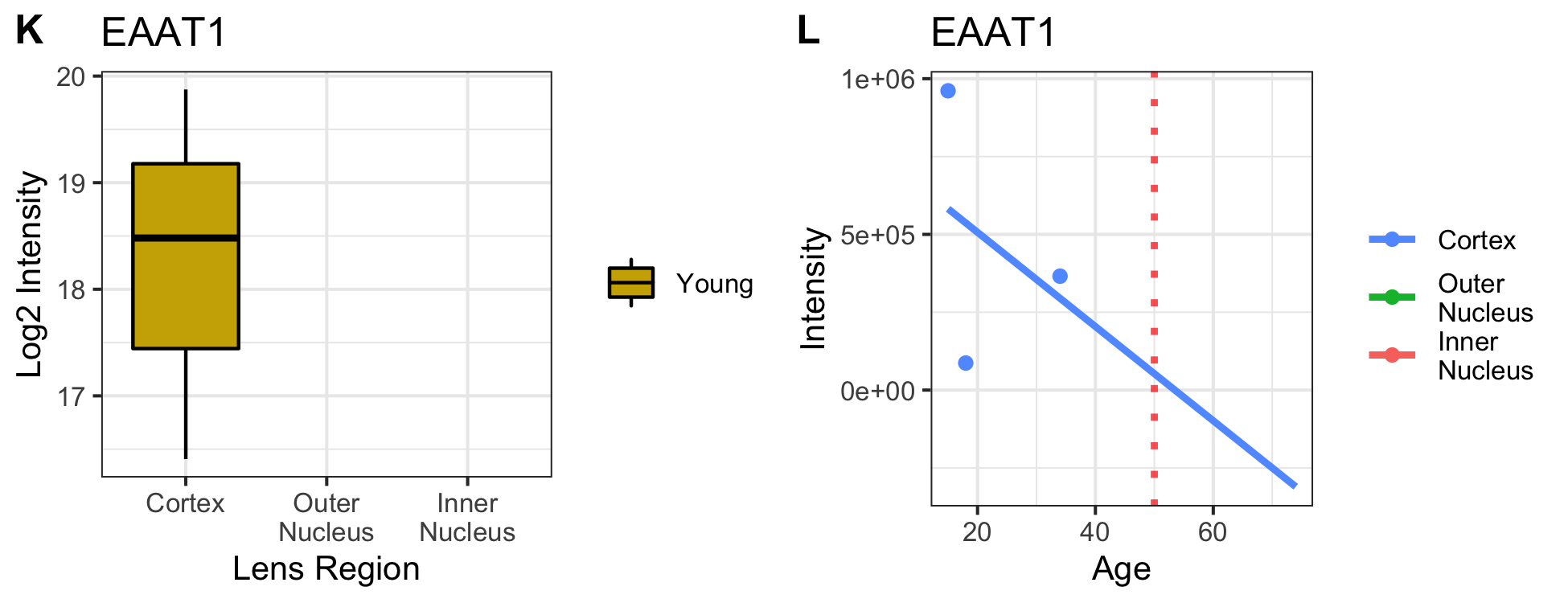

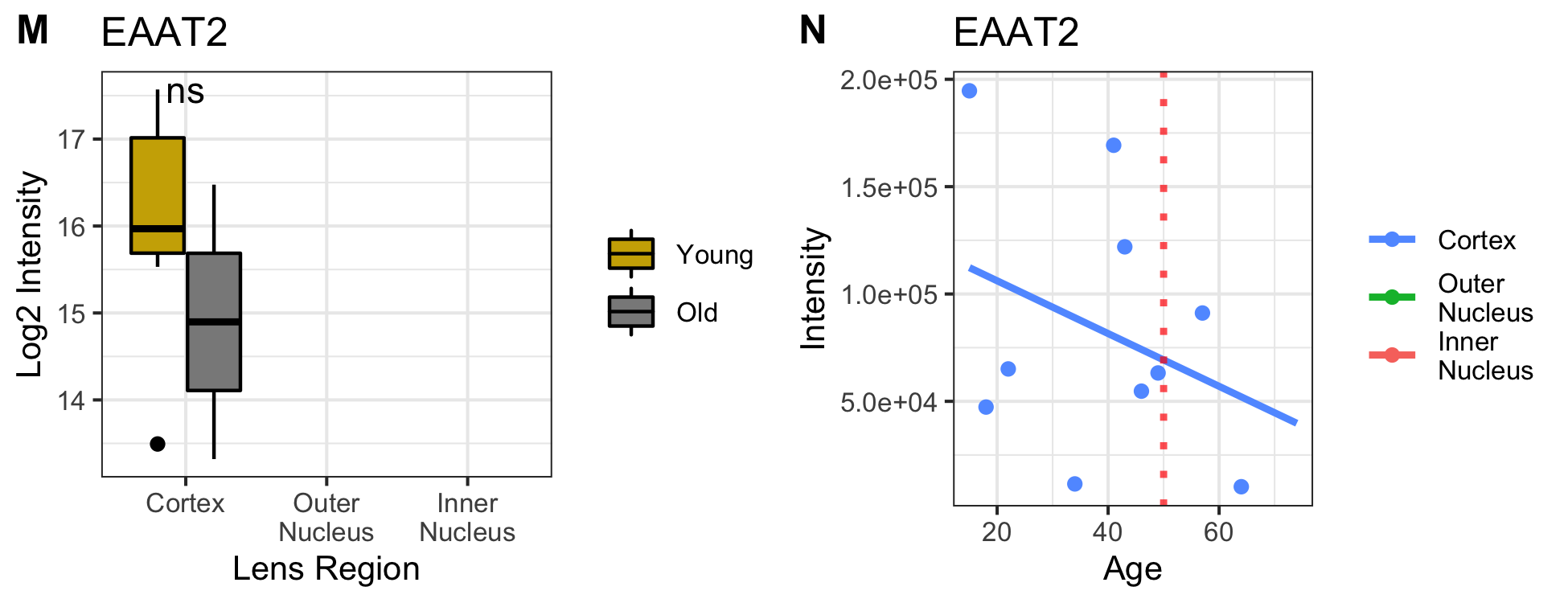

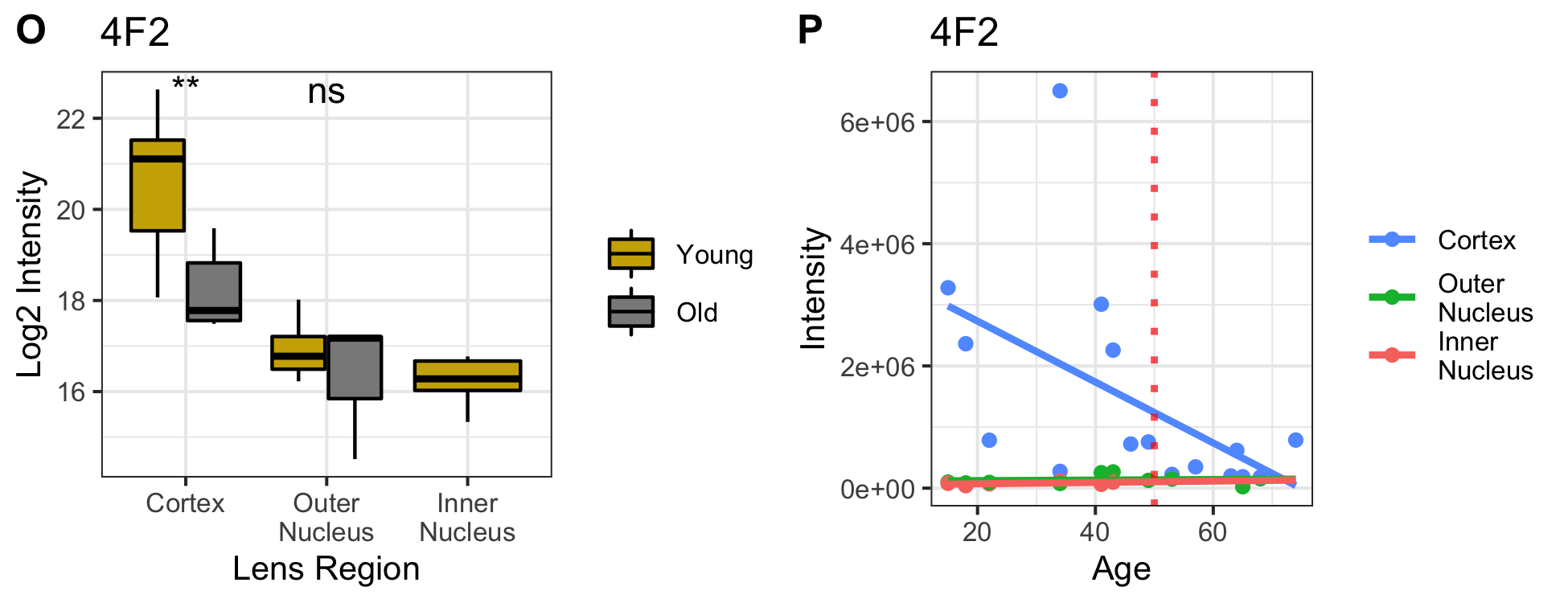

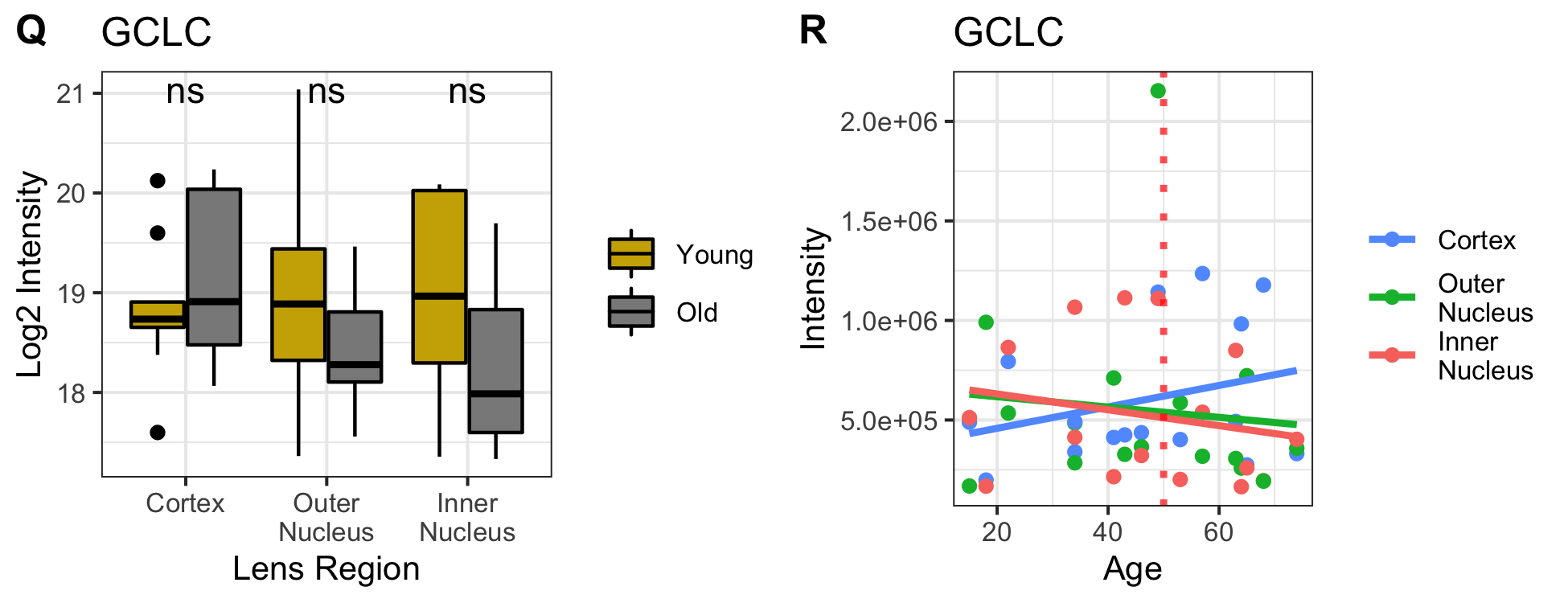

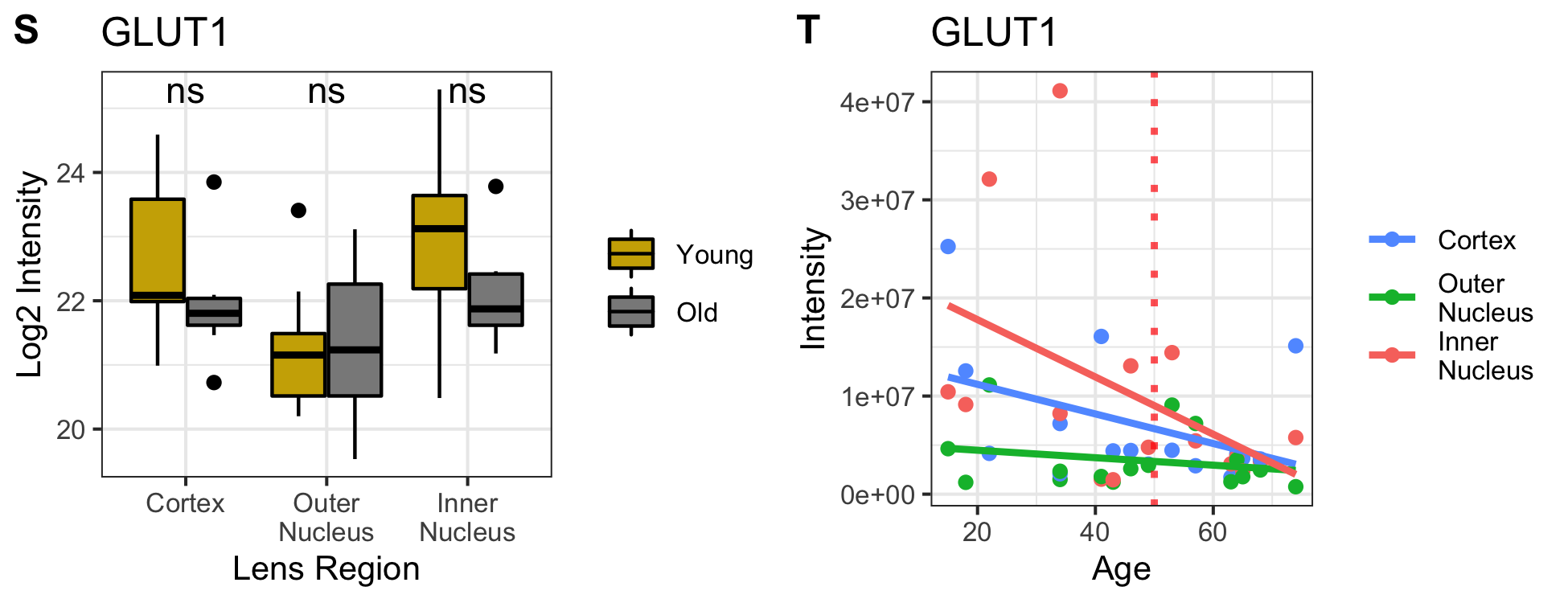

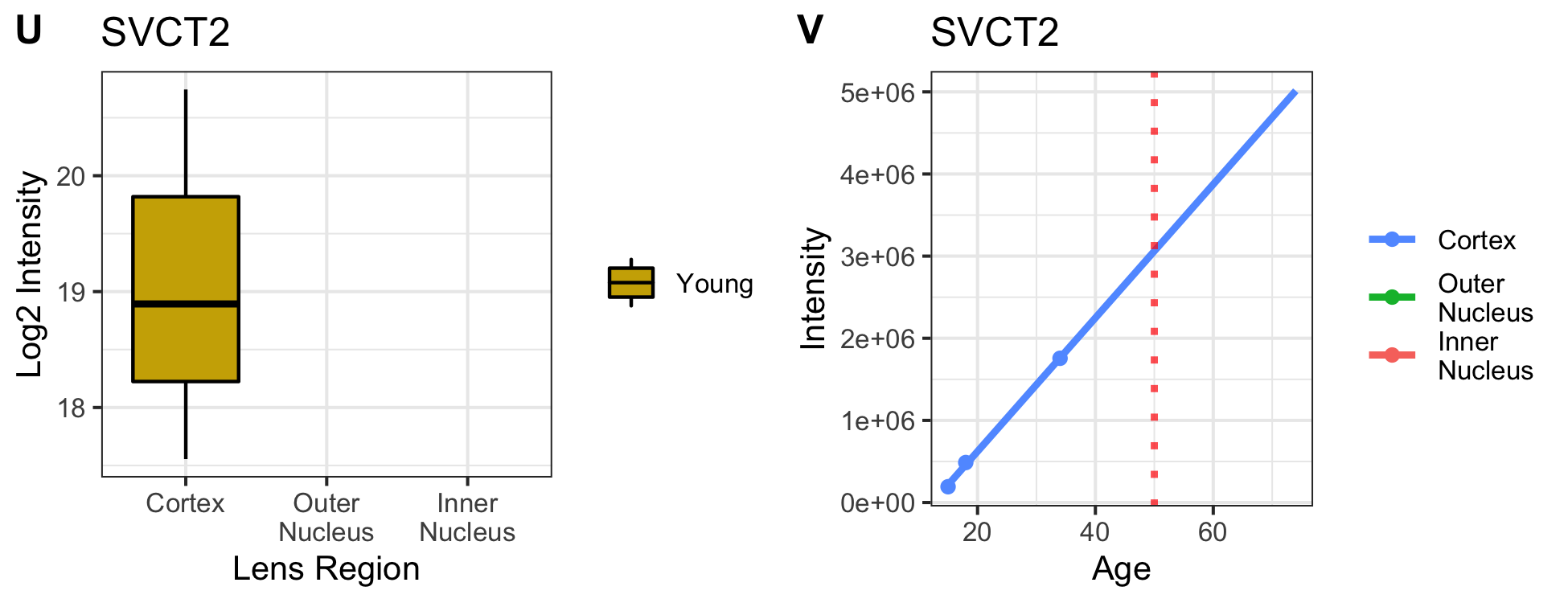


Supplemental Figure S19 - Age-related expression of each protein mentioned in the discussion as related to glutathione transport, glutathione synthesis or vitamin C transport. Proteins not measured are not plotted. T-test significance cutoffs were set at * = <0.05, ** = <0.01, *** = <0.001, **** =<0.0001


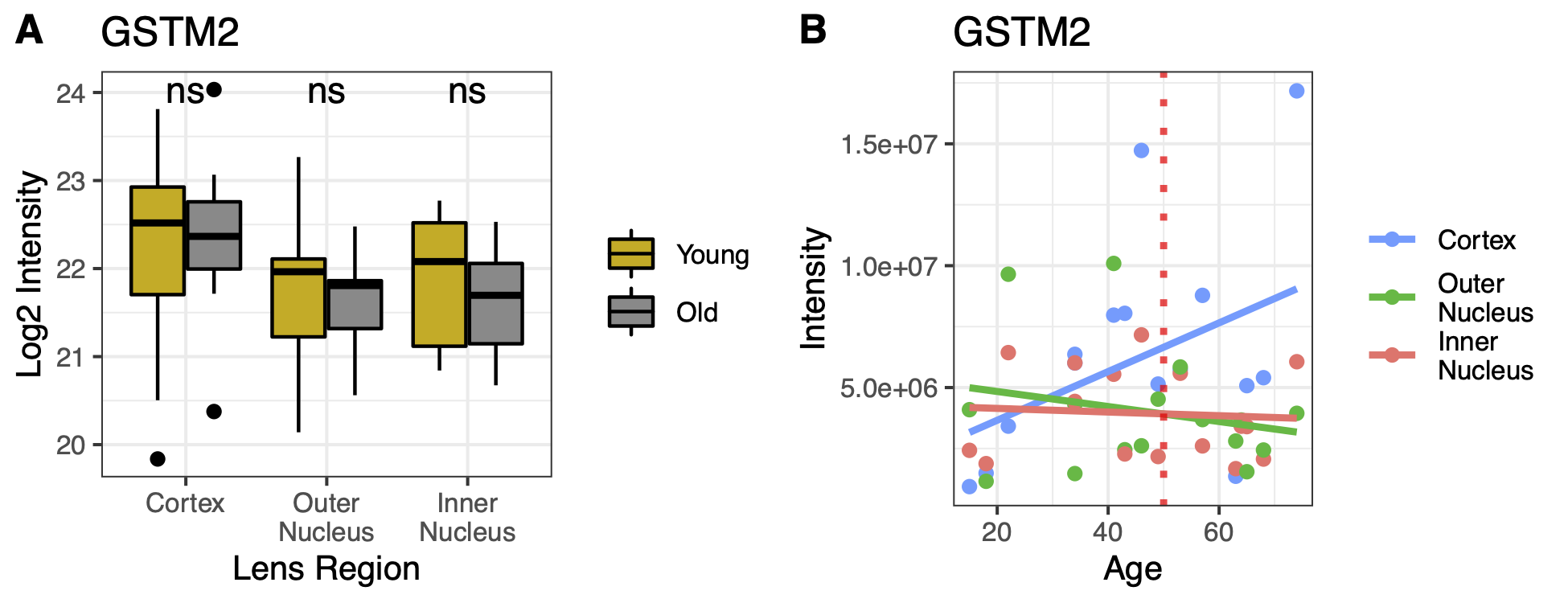

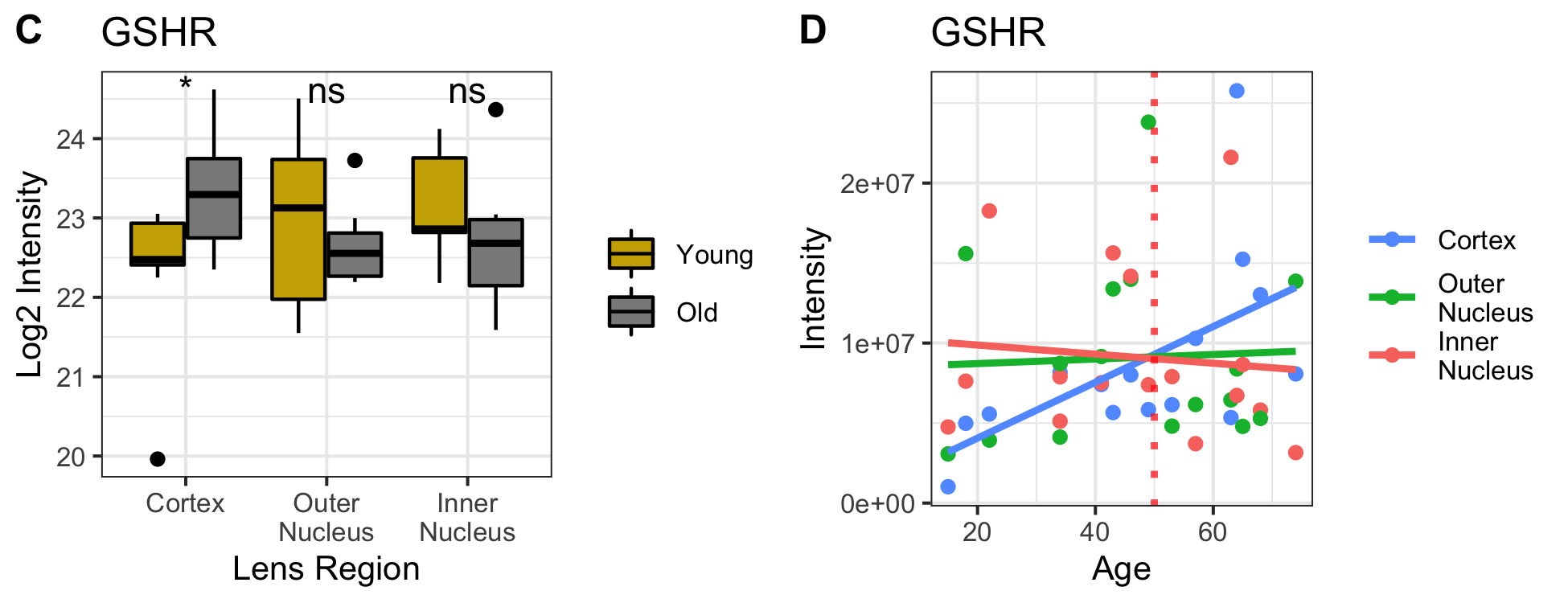


Supplemental Figure S20 – A,C) Age-grouped t-test (* = <0.05, ** = <0.01, *** = <0.001, **** =<0.0001) and B,D) age-related expression of A,B) glutathione-s-transferase mu 2 (GSTM2) and C,D) GSH reductase (GSHR). There is not a clear statistical change in the representation of either protein within the dataset and each protein is consistently measured throughout the lens. It is hypothesized that the accumulation of each cytosolic protein represents insolubilization by misfolding.


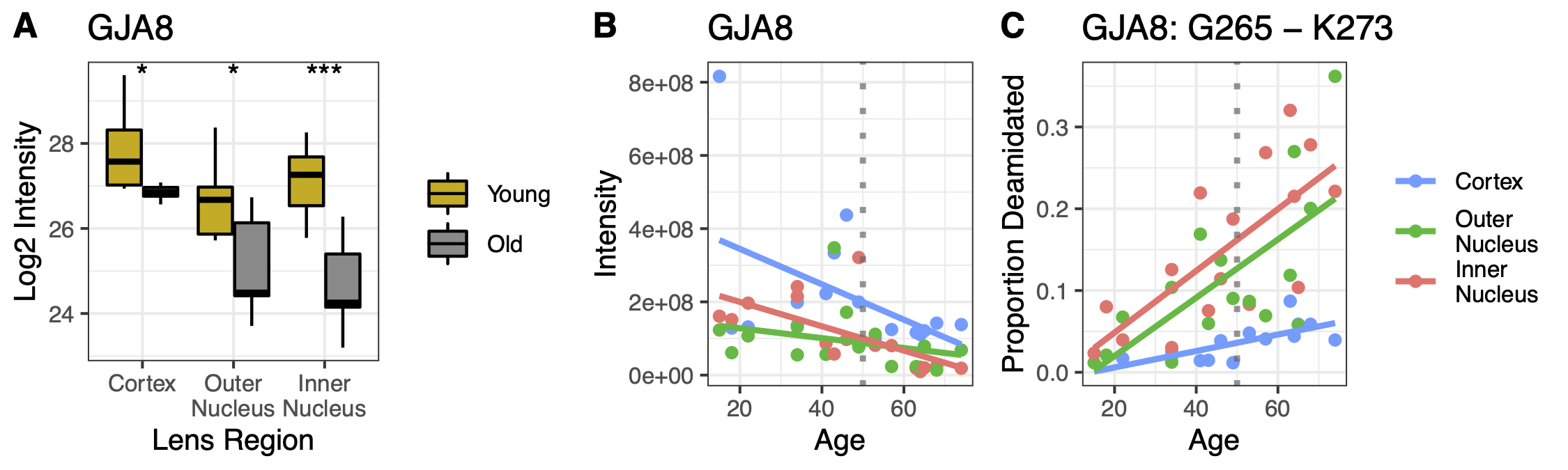


Supplemental Figure S21 - Demonstration of age-related abundance change of connexin 50 (GJA8) and A) An age-related decrease was appreciable for GJA8, especially in the inner nucleus, and B) decrease in protein expression cannot be solely attributed to PTMs, as shown by C) accumulation of deamidation on the G265-K273 peptide. D) Abundance of a cytoplasmic loop region peptide (E110-K139) for GJA8 demonstrates that cytoplasmic loop cleavage and functional deletion occurs in older nuclear lens regions after 50 years. T-test significances shown as * = <0.05, ** = <0.01, *** = <0.001, **** =<0.0001


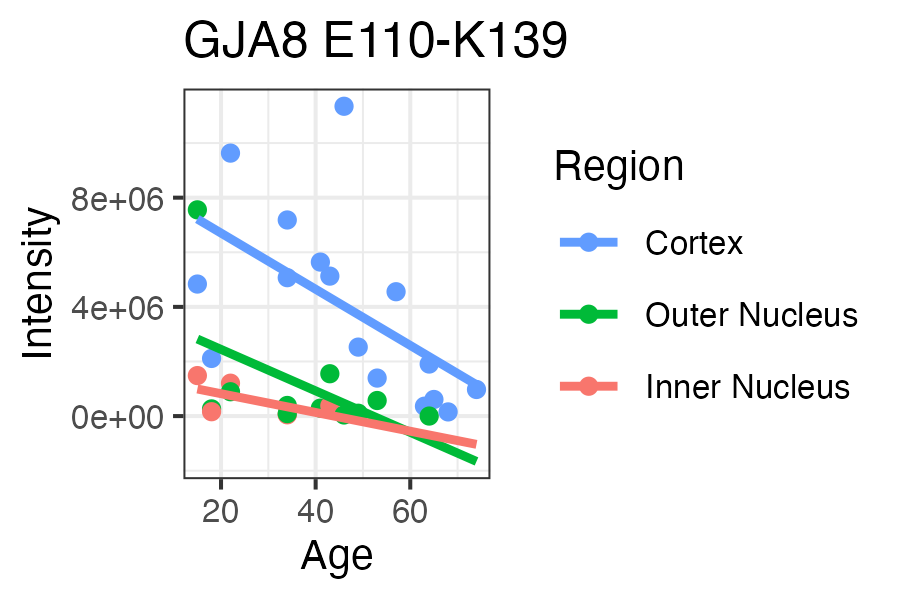


**D**


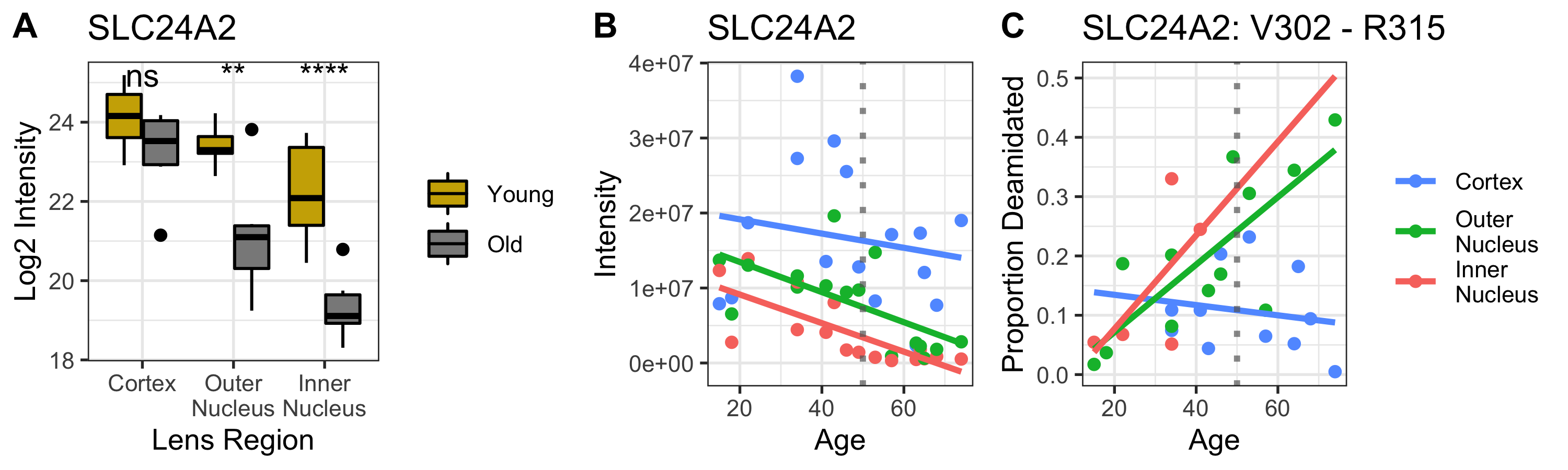


Supplemental Figure S22 - Demonstration of age-related abundance change of SLC24A2 (Sodium, Potassium, Calcium Exchange Protein 2). A) A proteome-remodeling related decrease in SLC24A2 abundance was appreciable in each nuclear region, especially the inner nucleus, and B) There is a steady linear decline of SCL24A2, however, there is a significant decrease in the abundance of measured SLC24A2 in the inner nucleus young region relative to old region, which is not consistent with linearity. C) Proportional deamidation of V302-R315 on SLC24A2 is demonstrated, showing some accumulation of deamidation with age, but that nuclear samples are less likely to be singly deamidated, suggesting further modification or decrease in abundance below the limit of detection and that PTMs alone are unlikely to explain the decrease in measured abundance. D, E) When t-test significance and linearity of protein abundance change was assessed with deamidation enabled, identical trends to those measured in the unmodified dataset were demonstrated. T-test significances shown as * = <0.05, ** = <0.01, *** = <0.001, **** =<0.0001


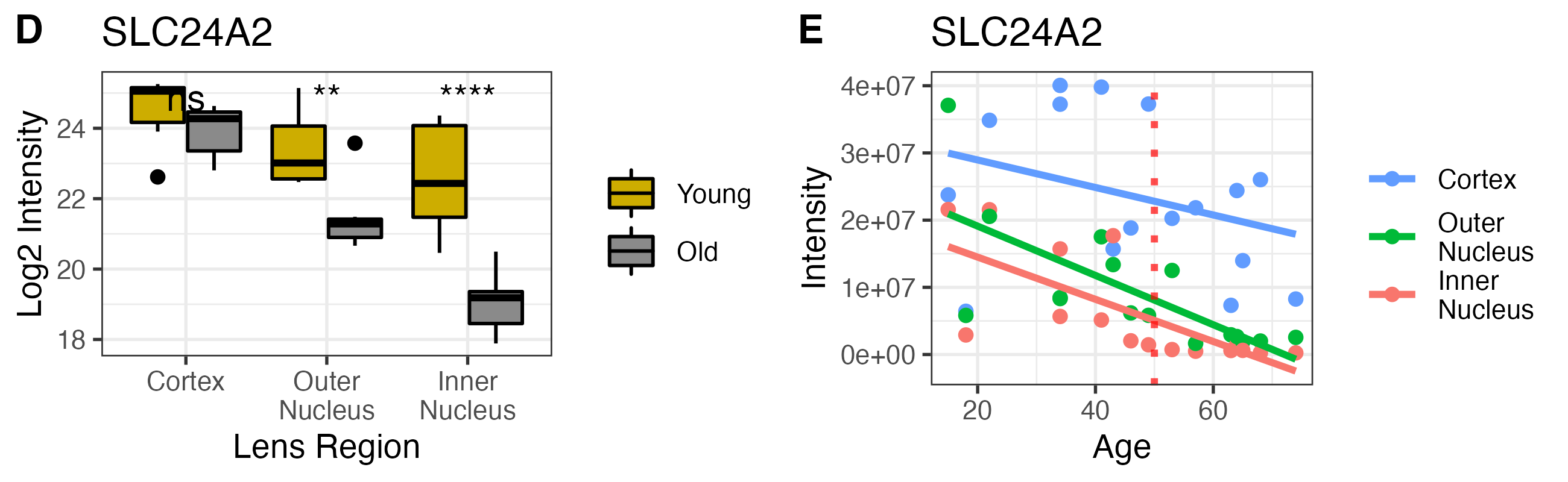

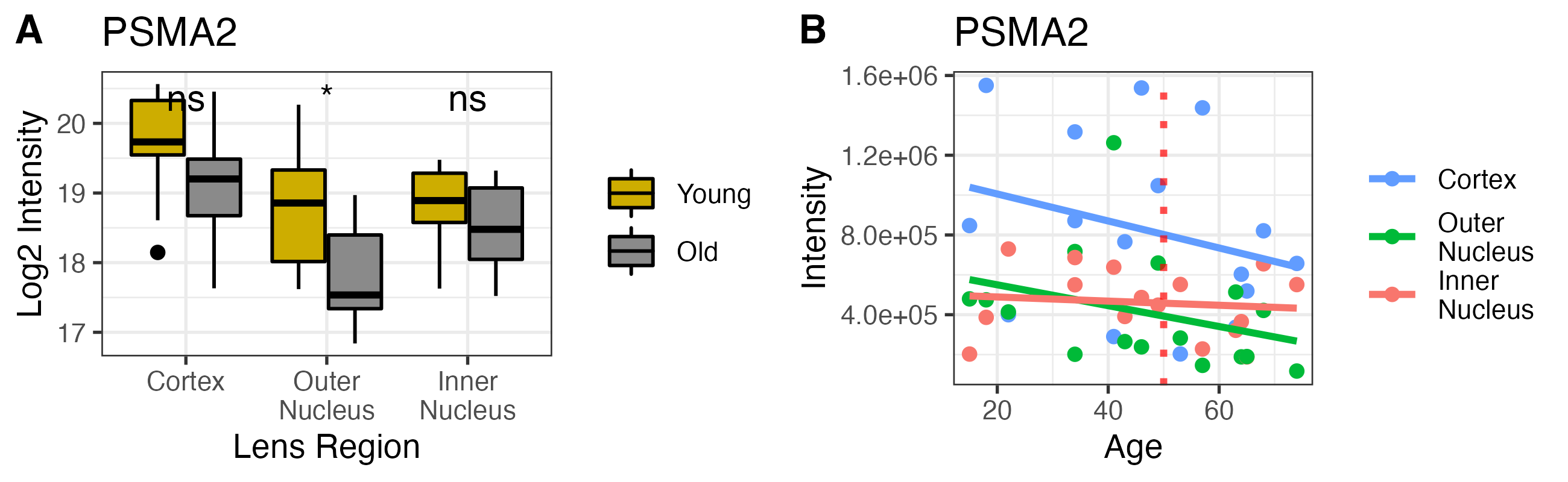

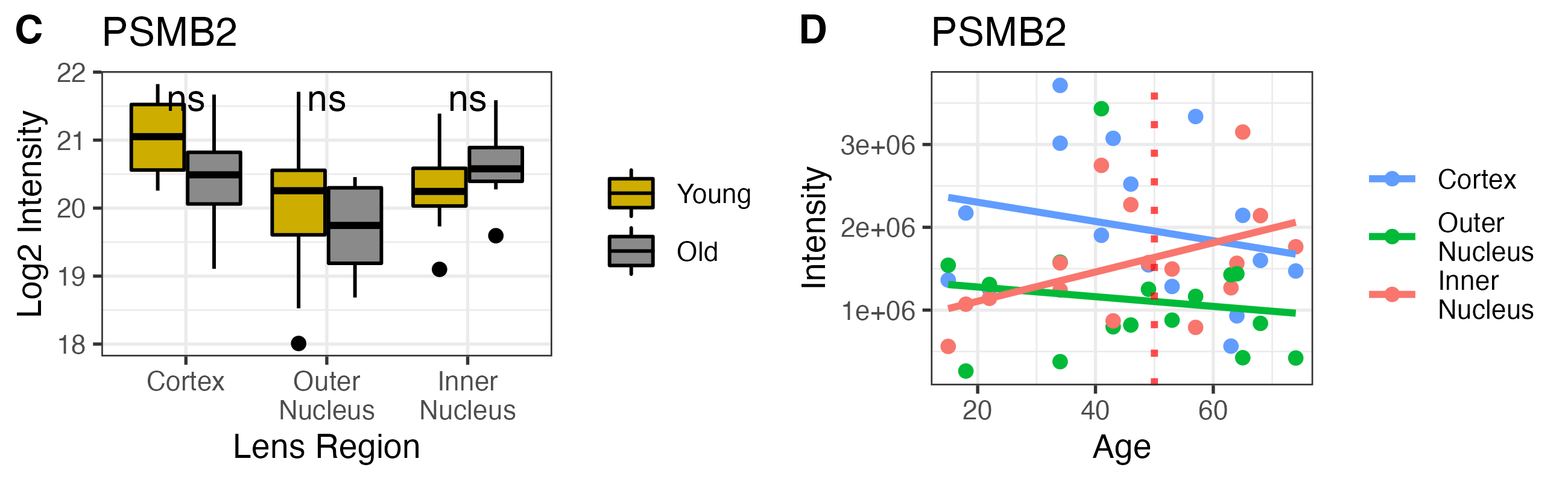

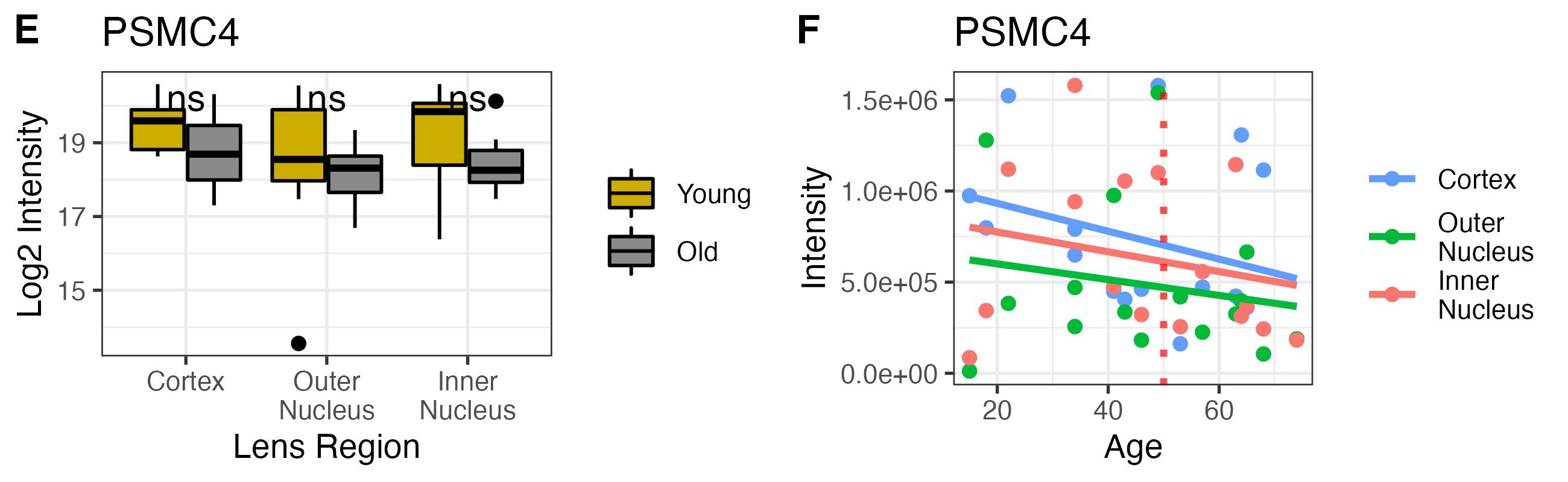

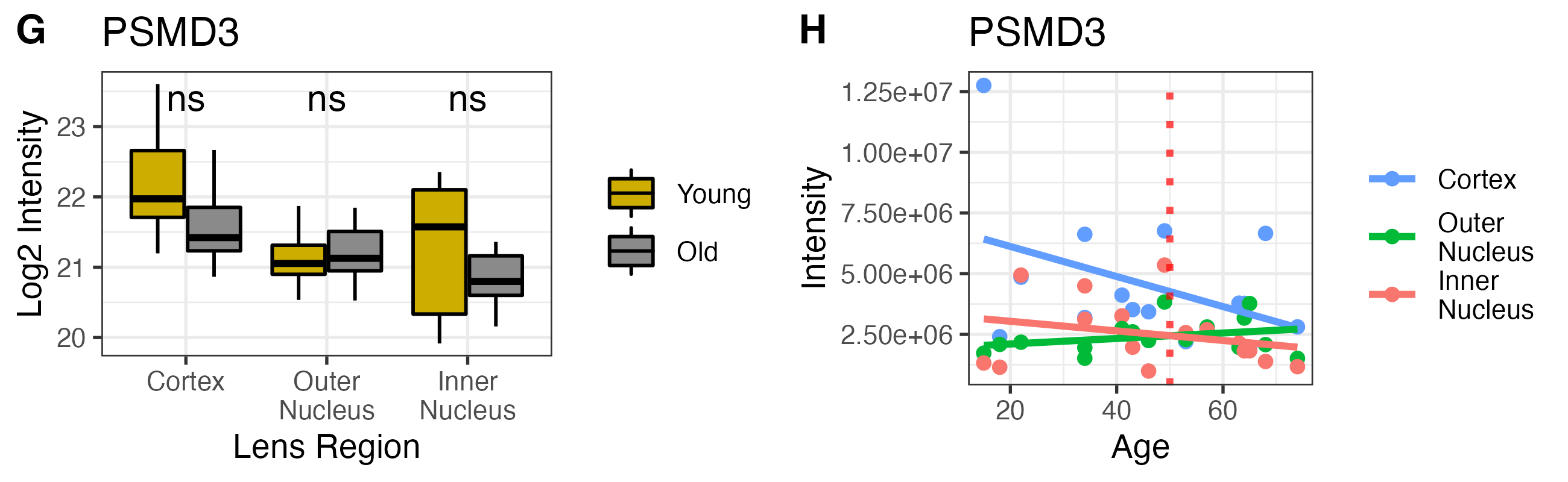

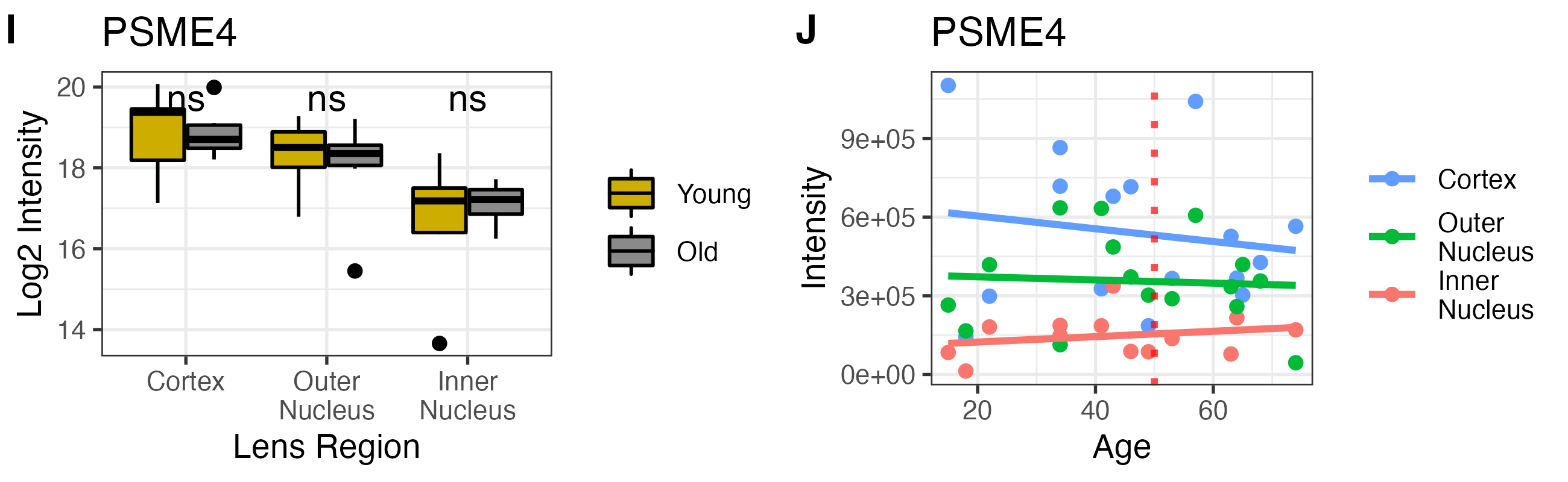


Supplemental Figure S23 – Evaluation of several proteasome components and their statistical change relative to proteome remodeling event. All samples demonstrate some fiber cell maturation stage related degradation, but there is no significant change in the abundance of each proteasome component with age. T-test significance cutoffs were set at * = <0.05, ** = <0.01, *** = <0.001, **** =<0.0001 for boxplot comparison.


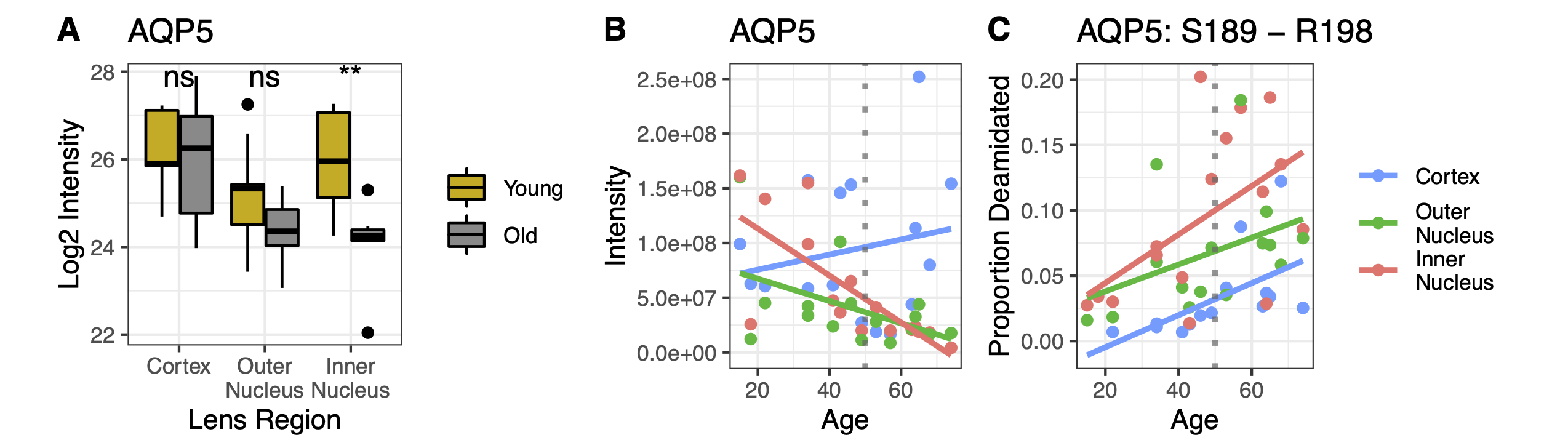


Supplemental Figure S24 – A) T-test comparisons (* = <0.05, ** = <0.01, *** = <0.001, **** =<0.0001) and distribution of AQP5 abundance relative to the proteome remodeling event and B) AQP5 distribution with age relation. Increase of cortical AQP5 is not necessarily indicative of increased cortical expression, but instead that AQP5 contributes proportionally to the lens proteome more significantly in several biological replicates. Distribution and t-testing demonstrates that this is statistically insignificant . C) The proportion of deamidation on C-terminal peptide G241-R253 demonstrates that the accumulation of deamidation alone is not responsible for the age-related decrease in AQP5 representation in the lens proteome.
